# Rapid *de novo* discovery of peptidomimetic affinity reagents for human angiotensin converting enzyme 2

**DOI:** 10.1038/s42004-022-00625-3

**Published:** 2022-01-19

**Authors:** Genwei Zhang, Joseph S. Brown, Anthony J. Quartararo, Chengxi Li, Xuyu Tan, Stephanie Hanna, Sarah Antilla, Amanda E. Cowfer, Andrei Loas, Bradley L. Pentelute

**Affiliations:** 1 Massachusetts Institute of Technology, Department of Chemistry, 77 Massachusetts Avenue, Cambridge, MA 02139 USA; 2 The Koch Institute for Integrative Cancer Research, Massachusetts Institute of Technology, 500 Main Street, Cambridge, MA 02142 USA; 3 Center for Environmental Health Sciences, Massachusetts Institute of Technology, 77 Massachusetts Avenue, Cambridge, MA 02139 USA; 4 Broad Institute of MIT and Harvard, 415 Main Street, Cambridge, MA 02142 USA; 5 Present Address: FogPharma, 30 Acorn Park Dr, Cambridge, MA 02140 USA

**Keywords:** Combinatorial libraries, Peptides, Screening

## Abstract

Rapid discovery and development of serum-stable, selective, and high affinity peptide-based binders to protein targets are challenging. Angiotensin converting enzyme 2 (ACE2) has recently been identified as a cardiovascular disease biomarker and the primary receptor utilized by the severe acute respiratory syndrome coronavirus 2. In this study, we report the discovery of high affinity peptidomimetic binders to ACE2 via affinity selection-mass spectrometry (AS-MS). Multiple high affinity ACE2-binding peptides (ABP) were identified by selection from canonical and noncanonical peptidomimetic libraries containing 200 million members (dissociation constant, *K*_D_ = 19–123 nM). The most potent noncanonical ACE2 peptide binder, ABP N1 (*K*_D_ = 19 nM), showed enhanced serum stability in comparison with the most potent canonical binder, ABP C7 (*K*_D_ = 26 nM). Picomolar to low nanomolar ACE2 concentrations in human serum were detected selectively using ABP N1 in an enzyme-linked immunosorbent assay. The discovery of serum-stable noncanonical peptidomimetics like ABP N1 from a single-pass selection demonstrates the utility of advanced AS-MS for accelerated development of affinity reagents to protein targets.

## Abstract (web summary)

Angiotensin converting enzyme 2 (ACE2) has been identified as a cardiovascular disease biomarker and the primary receptor utilized by SARS-CoV-2, but developing serum-stable, selective and high-affinity binders for this target is challenging. Here, the authors use affinity selection-mass spectrometry to identify multiple high affinity ACE2-binding peptides from canonical and noncanonical peptidomimetic libraries containing 200 million members.

## Introduction

The discovery of high affinity reagents is a critical initial step in drug discovery, diagnostic development, and proteome profiling^1–3^. High affinity ligands are under constant development for the modulation of activity or function of target proteins, including challenging protein–protein interactions (PPIs) and intracellular targets^4,5^. Diagnostics inform clinical decision making and require sensitive and selective detection within highly complex media often containing a broad range of other protein concentrations (e.g., plasma)^6^. Lastly, affinity reagents have been crucial for understanding the proteome, facilitating the compilation of a knowledge base for protein expression profiles and localization across normal and disease tissue (e.g., Human Protein Atlas)^2,7,8^. Yet, the vast majority of known proteins have no corresponding affinity reagent^3,9^. Overall, each of these fields relies on the rapid discovery and development of selective, high affinity binders to specific protein targets.

Current methods to discover high affinity reagents against protein targets vary in production speed and chemical diversity, ranging from antibody production to panning fully synthetic libraries. While they are the ‘gold standard,’ antibodies require a long production timeline, demonstrate low tissue penetration, and can exhibit batch variability^10,11^. Thus, several non-antibody systems including DNA-encoded libraries^12,13^, aptamers^14,15^, and peptide discovery platforms^16^, have been developed to discover specific, high affinity binders. Most ‘hit’ discovery techniques rapidly isolate and enrich binders from libraries based on their high affinity to the target protein, though high-throughput screening of individual compounds has also been used^17,18^. The design and diversity of these libraries greatly affect the rate of discovery against a novel target. Larger molecular scaffolds can bind broader portions of protein surfaces, enabling the efficient targeting of PPIs^3,5,15,19,20^. The chemical and structural diversity of the curated library can improve discovery success^17,18,21,22^. Thus, an ideal discovery platform should combine high chemical diversity and rapid responsiveness to new clinically relevant targets.

Much research has been devoted to the discovery and engineering of peptidomimetic binders because of their broad access to diverse chemical spaces, amenability to rapid synthesis or modification, and availability of multiple rapid discovery platforms^21,23^. The use of noncanonical amino acids, macrocyclization, and chemical stapling, in particular, have been proven to be useful in promoting cell uptake, proteolytic stability, and improved pharmacokinetics^16,21,24–27^. Discovery via affinity selection using genetically-encoded techniques including phage display^28,29^ and mRNA display^30^ samples vast libraries up to 10^13^ members, being amenable for *de novo* discovery of high affinity reagents. However, these techniques are not well suited to the incorporation of highly noncanonical library members, even in cell-free systems^31–34^. Thus, following the initial identification of high affinity peptides, further development is required via iterative synthetic cycles of derivatization and screening.

High affinity peptidomimetic binders can also be identified by affinity selection-mass spectrometry (AS-MS), enabling the straightforward use of entirely noncanonical synthetic libraries without expending rapid discovery^35–37^. AS-MS generally functions through the enrichment and identification of peptidomimetic binders to the target protein through a single enrichment step since it cannot be genetically amplified. Thus, discovery efforts with AS-MS have generally been limited to small combinatorial libraries (10^3^–10^6^ members), which were biased toward the target protein in a ‘focused’ or structure-based manner^38,39^. AS-MS of these focused libraries remains a reliable way to rapidly identify key binding ‘hot-spot’ residues and combinatorically introduce noncanonical amino acids^38,40,41^. Recent advancements made by our group in the MS/MS sequencing of complex peptidomimetic mixtures^42^ and optimized AS-MS selection conditions^43^ have enabled *de novo* discovery of high affinity binders from fully randomized peptidomimetic libraries up to 10^8^ members^43^. Thus, AS-MS can enable rapid discovery across highly diverse libraries^44,45^. With these methods, we set out to perform *de novo* discovery with synthetic highly noncanonical peptidomimetic libraries against recently identified clinically relevant targets.

Angiotensin converting enzyme 2 (ACE2) has been identified as an important plasma biomarker for cardiovascular disease-induced events and death in a global, population-based study^46,47^. Also, ACE2 is ubiquitously known as the receptor utilized for cell entry by the severe acute respiratory syndrome coronavirus 2 (SARS-CoV-2) beta-coronavirus^48,49^. Thus, high affinity reagents for the specific serum detection of ACE2 are increasingly important. Here we demonstrate rapid discovery of high affinity peptidomimetic binders to ACE2 through a single-pass AS-MS experiment utilizing fully randomized canonical and noncanonical libraries. By comparing selection results from the noncanonical library over a ‘standard’ canonical library quantitatively and qualitatively, we highlight that the noncanonical binders exhibit improved proteolytic serum stability. In further tests, our noncanonical peptidomimetic ACE2 binder, ABP N1, demonstrated ACE2 binding specificity in a serum pulldown experiment and as low as picomolar detection in an enzyme-linked immunosorbent assay (ELISA), highlighting the development as promising diagnostic tools.

## Results

### AS-MS identifies low-nanomolar affinity canonical and noncanonical peptide binders to ACE2

**Fig. 1:**
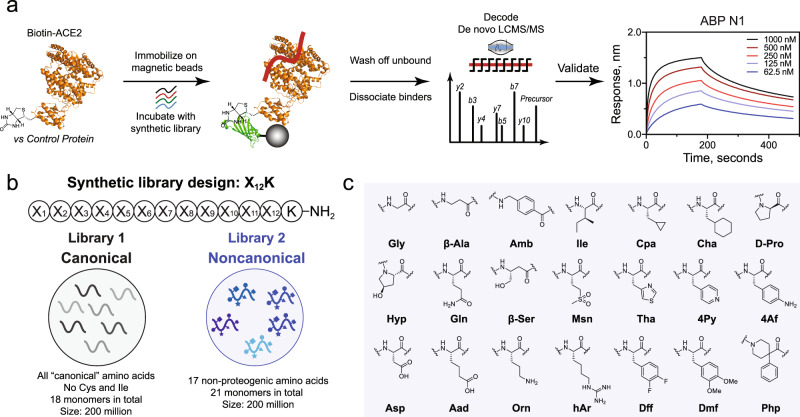
Magnetic bead-based affinity selection-mass spectrometry (AS-MS) enables rapid discovery of both canonical and noncanonical binders in a single experiment. **a** Schematic representation of the AS-MS workflow used in this study. In brief, the biotinylated protein (ACE2 represented in orange from PDB: 6M17 or control) was immobilized onto streptavidin (SA)-coated magnetic beads and then sampled in synthetic peptide libraries to enrich peptide binders. Subsequently, unbound peptides were washed away, and bound material was eluted and then sequenced by nLC-MS/MS. Individual hits were synthesized and validated at the final step. **b** The design for synthetic canonical and noncanonical libraries. **c** The monomer set used for the synthesis of the noncanonical library (Library 2).

We recently optimized in-solution affinity selection combined with nano-liquid chromatography-tandem mass spectrometry (nLC-MS/MS) sequencing to enable the identification of high affinity binders from fully randomized synthetic libraries with a diversity of up to 10^8^ members, a 100-fold increase over the standard practice^43^. Noncanonical amino acids can be extensively used in the preparation of the synthetic libraries utilized in AS-MS, provided there is no isobaric monomer mass overlap and sufficient tandem sequencing fidelity. Thus, we sought to compare the results of our selections against human ACE2 protein using a standard, canonical-L library, and a noncanonical-L library, each containing 200 million members (Fig. 1a).

The two libraries share the same design, 12 variable positions followed by an amidated C-terminal lysine to facilitate sequence identification and filtering^42^. The monomer set utilized in the canonical library (Library 1) is fully proteogenic, except Cys because it could form intra- or intermolecular disulfides and Ile because it is isobaric in mass with Leu, to give 18 amino acids total (Fig. 1b). For side-by-side comparison and exploration of noncanonical chemical space, another library (Library 2), composing 17 non-proteogenic monomers out of 21 amino acids total (81% noncanonical, significantly more than the available genetically encoded techniques) was synthesized (Fig. 1b, c)^31–34^. Library 2 was designed to sample an entirely new chemical space, while still capturing similar chemical properties of the natural amino acid set. While nearly all the canonical monomers were replaced with non-proteogenic analogs, the number of positively and negatively charged residues at physiological pH was kept constant at 2 each, respectively. Nonstandard backbones including β-amino acids and achiral linkers were included to diversify structural availability and improve proteolytic stability. The final monomers were selected based on considerations of balanced chemical diversity, mass uniqueness, library solubility, stereochemical purity, and compatibility with Fmoc solid-phase peptide synthesis (SPPS).

Following the workflow depicted in Fig. 1a, we performed the affinity selection using both the canonical and noncanonical library against human ACE2 in parallel with 12ca5 as an unrelated control protein, and the enriched peptides were eluted and analyzed by nLC-MS/MS. The sequences were decoded using the PEAKS software^50^ and filtered^42^ to isolate peptidomimetic sequences that matched the original library design. The peptidomimetics were sorted to reveal those that were unique to ACE2 in comparison to the off-target control protein and are reported in Supplementary Tables 2 and 3. From both the canonical and noncanonical selections, we observed a preferred N-terminal motif, indicating a potential new class of ACE2 binders (see Supplementary Note 1 and Supplementary Figs. 1 and 2). For the canonical L-peptides, leucine (L) and valine (V) were preferred at the N-terminus position followed by glutamine (Q) and asparagine (N) with some cationic or ionizable residues nearby (H, K, and R, see Supplementary Fig. 1). Similarly, the noncanonical L-peptidomimetics discovered preferred cyclopropylalanine (Cpa, C) and isoleucine (Ile, I) at the N-terminal position followed by 3-(4-thiazolyl)-alanine (Tha, T, see Supplementary Fig. 2). The observation of a preferred motif added confidence in the discovery of a class of peptidomimetic binders to ACE2.

An additional refinement step was taken to analyze the extracted ion chromatogram (EIC) of the observed ion from each MS-identified peptide. This EIC-based refinement provides an additional level of confirmation that each peptide was uniquely selected against ACE2 or if it binds nonspecifically to the selection matrix (e.g., magnetic beads or streptavidin). A comparable number of sequences was identified as EIC-selective between the two libraries (48 noncanonical sequences and 60 canonical sequences), indicating that sequencing and identification of noncanonical binders can robustly be achieved with a similar throughput to canonical peptides on our AS-MS platform using Orbitrap nLC-MS/MS. However, the noncanonical selection showed a higher primary MS baseline that obscured more peptidomimetics from being discovered (Supplementary Table 3; Supplementary Fig. 17). This ambiguity could be explained by a higher level of baseline binding from the noncanonical peptidomimetics library or poorer ionization of the eluted library members. Lastly, both selections showed a low rate of nonspecific binder recovery (Supplementary Fig. 18).

**Fig. 2:**
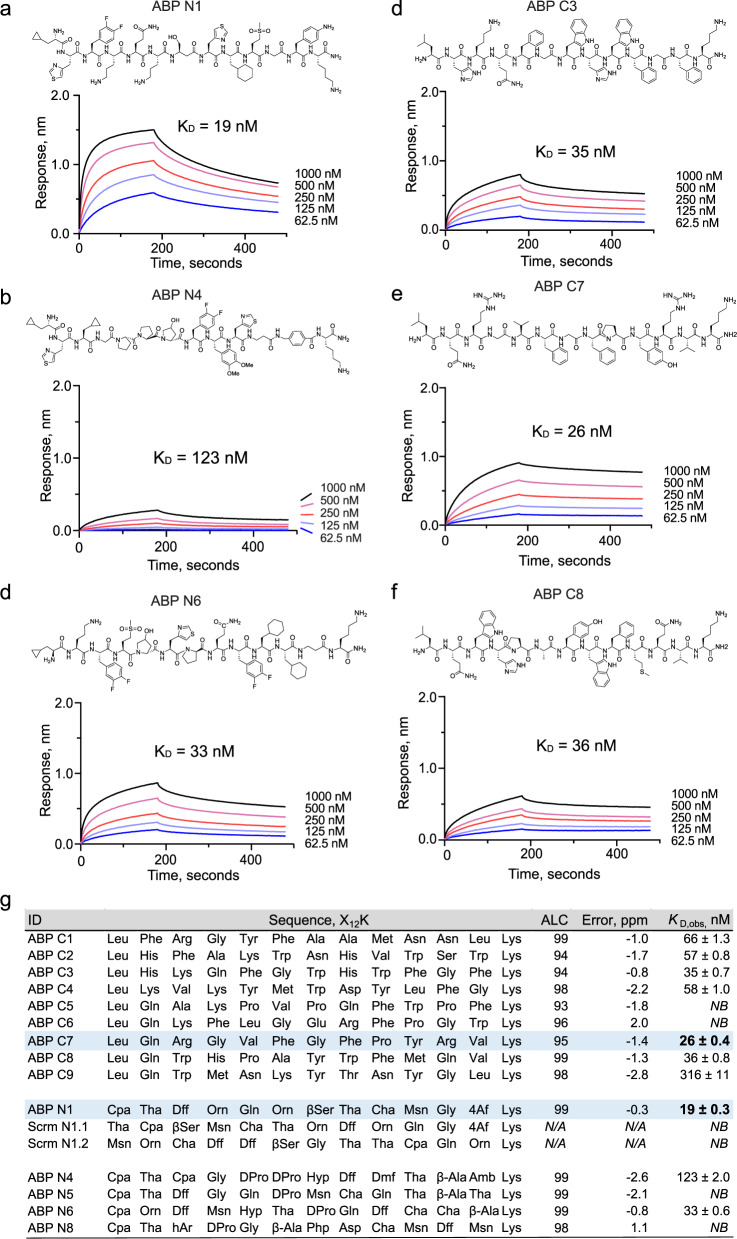
Nanomolar affinity binders were identified from both canonical and noncanonical libraries. **a**–**c** ACE2-binding traces, measured by bio-layer interferometry, for noncanonical binders ABP N1, ABP N4, and ABP N6, respectively. **d**–**f** ACE2-binding traces, measured by bio-layer interferometry, for canonical binders ABP C3, ABP C7, and ABP C8, respectively. **g** A summary of all individually synthesized peptides. Column headers: ‘ID’, the peptide identifiers; ‘Sequence, X_12_K’, the peptide sequences with a lysine at the C-terminus; ‘ALC’, the exported average local confidence score from sequence decoding; ‘Error, ppm’, the mass error (in ppm) between the precursor and assigned sequence; ‘*K*_D_, _obs_, nM’, the apparent dissociation constant, in nM, measured by bio-layer interferometry. Cyan highlights the canonical (ABP C7) and noncanonical (ABP N1) peptides with the highest binding affinity.

With the discovered sequences in hand, nine canonical and five noncanonical binders were chosen for synthesis and validation efforts based on their high average local confidence (ALC) scores. Each binder was re-synthesized and purified individually (see Supplementary Notes 2 and 3) and their binding affinities (dissociation constant, *K*_D_) to ACE2 were measured using bio-layer interferometry (BLI, see Supplementary Note 4). As a result, low-nanomolar ACE2-binding affinities were determined for the noncanonical binders ABP N1 (*K*_D_ = 19 nM, Fig. 2a), ABP N4 (*K*_D_ = 123 nM, Fig. 2b), and ABP N6 (*K*_D_ = 33 nM, Fig. 2c), and for the canonical binders ABP C3 (*K*_D_ = 35 nM, Fig. 2d), ABP C7 (*K*_D_ = 26 nM, Fig. 2e), and ABP C8 (*K*_D_ = 36 nM, Fig. 2f). All other peptides either presented lower affinity or no binding to ACE2 (Fig. 2g), indicating that approximately half of the ACE2-selective sequenced binders following AS-MS were non-binders by BLI.

To determine whether the observed binding is sequence-specific, two scrambled variants of ABP N1 (Scrm N1.1 and Scrm N1.2) were synthesized and their binding to ACE2 was tested by BLI. Under these conditions, no binding to ACE2 was observed (Fig. 2g; Supplementary Fig. 21). We also observed minimal binding to an unrelated protein (12ca5) for ABP N1, ABP N4, and ABP N6 (Supplementary Fig. 22). However, the binding of 500 nM of ACE2 to ABP N1 results in ~1.3 nm BLI response signal, compared to 0.25 nm signal from 500 nM of the off-target protein (12ca5), translating to ~ 5-fold higher signal and indicating selectivity toward ACE2.

Additionally, we investigated whether known binders to ACE2 inhibited the binding of the ABPs, including the native ACE2 substrate angiotensin 2 (AngII)^51^, an ACE2 inhibitor MLN-4760^52,53^, and the receptor binding domain (RBD) of SARS-CoV-2 (see Supplementary Notes 5 and 6)^48,49^. First, we performed competitive binding experiments on all binders discovered (ABP N1, N4, N6, C1, C2, C3, C4, C7, and C8) versus AngII and MLN-4760 (Supplementary Figs. 27–35). We observed that the native ACE2 substrate AngII did not inhibit the binding of any of our ABPs, indicating that the binding sites of ABPs and AngII likely do not overlap. However, MLN-4760 was able to partially block the binding of ABP N1 and C8 at the 10-fold excess of MLN-4760 mixed with ACE2, indicating that the binding sites likely overlap with the active site of the ACE2 enzyme. Secondly, we performed a competitive BLI experiment to determine if any of the discovered binders could disrupt the interaction between ACE2 and SARS-CoV-2 spike protein receptor binding domain (RBD). We did not observe any competition by our peptides on the binding of ACE2 to RBD (Supplementary Figs. 23–26) at 5- or 50-fold excess, indicating these ABPs do not bind at the RBD site of interaction.

### The noncanonical binder ABP N1 demonstrates enhanced serum stability

**Fig. 3:**
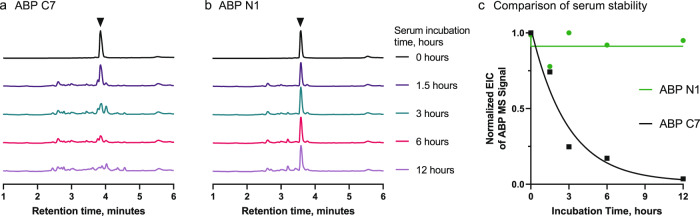
ABP N1 demonstrates enhanced serum stability relative to the canonical binder ABP C7. LC-MS chromatograms (total ion current) of ABP N1 (**a**) and ABP C7 (**b**) incubated at 37 °C in 1 × PBS (*t* = 0 h) or 5% normal human serum. Spectra were normalized to the intensity of the peptide at *t* = 0 h to observe degradation of the original amount of peptide. **c** Comparison of the serum stability of ABP N1 and ABP C7 by the integral of the extracted ion count (EIC) of the monoisotopic [M + 2H]^2+^ ion of the starting peptide. ABP C7 is quickly degraded and the starting peptide mass disappears over 12 h, with a calculated half-life of approximately 2 h (single phase decay model).

A significant limitation to the development of canonical L-peptides is their susceptibility to enzymatic degradation within physiological environments. Thus, the use of noncanonical amino acids, macrocyclization, and chemical stapling have each grown as approaches to improve their stability and pharmacokinetics^16,21,24–27^. To determine whether our noncanonical binders present improved stability over the canonical binders, we performed a serum stability assay on the two most potent variants: noncanonical ABP N1 and canonical ABP C7. After incubating both peptides in 5% normal human serum at 37 °C over time, serum proteins were precipitated by addition of trichloroacetic acid, and the binders retained in the supernatant were subjected to LC-MS analysis. As shown from the LC-MS traces, we observed significant degradation of ABP C7 over 12 hours when compared with the no-serum control (Fig. 3a), with an approximate half-life of 2 h (Fig. 3c). However, little-to-no degradation of ABP N1 was observed (Fig. 3b, c), even after 12 h of incubation. ABP N1 presented enhanced stability in human serum, and the side-by-side comparison with a canonical binder from a similar library selection demonstrated the immediate benefit of employing noncanonical libraries. Thus, we could rapidly proceed to determine the binding capability of ABP N1 in biological milieu.

### ABP N1 pulls down ACE2 from human serum selectively

**Fig. 4:**
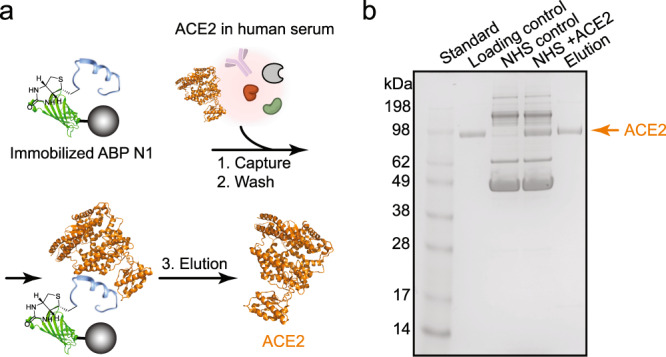
ABP N1 pulls down ACE2 from human serum selectively. **a** Schematic representation of the serum pull-down experiment. ABP N1 was immobilized onto streptavidin-coated magnetic beads and then incubated with ACE2 (represented in orange from PDB: 6M17) in human serum. Bound ACE2 was eluted for subsequent analysis. **b** The SDS-PAGE image with samples showing from left to right lanes: (1) molecular weight standard; (2) purified ACE2 protein (1.5 µg) as a loading control; (3) normal human serum as a control; (4) normal human serum mixed with ACE2 (1.5 µg); (5) elution of the bound fraction from the magnetic beads. NHS, Normal Human Serum (5%).

To further investigate the ACE2-binding capability of the identified high affinity reagents within a biological matrix and demonstrate binding selectivity, we performed a human serum pull-down experiment using the most potent noncanonical binder ABP N1. As depicted in Fig. 4a, the biotinylated ABP N1 was immobilized onto streptavidin-coated magnetic beads followed by incubation with a mixture of normal human serum and ACE2 protein. After removing the supernatant and washing off the unbound fraction, the bound material was eluted with a high concentration of urea and analyzed using the sodium dodecyl sulfate–polyacrylamide gel electrophoresis (SDS-PAGE). We observed a selective enrichment of ACE2 protein from the human serum complex, and no other proteins were pulled down by ABP N1 (Fig. 4b). The ability of selective binding and isolation of ACE2 from a complex biological matrix indicates a promising diagnostic application of ABP N1.

### Picomolar ACE2 can be detected with ABP N1 via ELISA

**Fig. 5:**
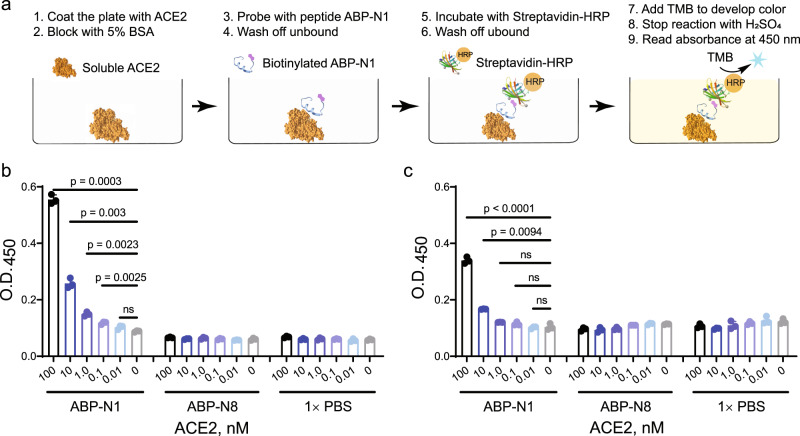
Picomolar ACE2 concentration was detected by ABP N1 by ELISA. **a** Schematic representation of the ELISA workflow. **b** ACE2 in 1 × PBS at different concentrations (100 nM–10 pM) was immobilized on an ELISA plate. **c** ACE2 in human serum at different concentrations (100 nM–10 pM) was immobilized on an ELISA plate. The plate was incubated with ABP N1 or control peptide, followed by streptavidin-HRP and TMB substrate. Absorbance was measured at 450 nm. The experiment was performed in technical triplicates (*n* = 3). Each data point, the mean signal as a bar, and error bars from experimental standard deviation, and statistical significance calculated with the unpaired Student’s *t*-test are shown.

Human ACE2 was recently identified as the top biomarker for cardiovascular disease and an elevated level of plasma ACE2 significantly associates with death, heart failure, stroke, and myocardial infarction^47^. To demonstrate the utility of our noncanonical ACE2 binders as detection probes of the plasma ACE2 level, we developed an ELISA-based detection assay (Fig. 5a). After immobilization on an ELISA plate, the picomolar concentration of ACE2 was detected by biotinylated ABP N1 in a dose-dependent manner (Fig. 5b). As a negative control, no binding was observed from biotinylated ABP N8 at the tested ACE2 concentrations (10 pM–100 nM in Fig. 5b), which is consistent with the non-binding observation as demonstrated in Fig. 2g. Interestingly, when a similar ELISA experiment was performed with the plate being coated with a mixture of normal human serum and exogenous ACE2, the dose-dependent ACE2 detection using ABP N1 was retained (Fig. 5c). However, we observed a decreased detection sensitivity presumably due to the decreased amount of ACE2 being immobilized on the plate with many other serum proteins present. As a summary, with an ELISA-based approach, we demonstrated that ABP N1 could selectively detect soluble ACE2 from the human serum at concentrations ranging from picomolar to low nanomolar levels.

## Discussion

In this study, we report rapid discovery of high affinity peptidomimetic binders to ACE2 via AS-MS. After screening two ultra-large synthetic libraries, a series of ACE2-binding peptides with low-nanomolar affinities (*K*_D_ from 19 to 123 nM) were identified. We showed that the most potent noncanonical peptide binder, ABP N1, demonstrated enhanced serum stability in comparison with the most potent canonical binder, ABP C7. Furthermore, ABP N1 demonstrated a high selectivity to ACE2 over human serum proteins, indicated by a serum pull-down experiment. Lastly, in an ELISA-based format, picomolar to low nanomolar ACE2 concentrations in human serum could be detected using ABP N1. The *de novo* discovered high affinity reagents offer specific serum detection of ACE2 and represent a promising diagnostic tool of related diseases.

We chemically synthesized both canonical and noncanonical peptide libraries (each containing 200 million peptides) and subjected them to a single-pass affinity selection experiment as a side-by-side comparison. Of note, a comparable number of putative binders was observed from both canonical and noncanonical library selections, with a similar portion of binders measured by BLI to show no apparent binding to ACE2. Therefore, even though the binders were identified as ACE2-selective with high sequencing confidence, individual synthesis and validation of the putative hits are necessary to confirm ACE2-specific interaction. We observed an N-terminal motif from both the canonical and noncanonical selections, indicating a potentially preferred binder class to ACE2 (Supplementary Figs. 1 and 2). However, after individual validation, binders containing such a motif do not warrant ACE2-binding capability (Fig. 2g), indicating that the other amino acids within the sequence also play an important role in binding. Notably, all of the discovered binders are linear, whereas previous phage display efforts to discover linear ACE2 binding peptides were unsuccessful and only discovered macrocyclic binders^54^.

Even though the canonical or noncanonical binders discovered here are linear, they do not have a clear similarity to other known binders of ACE2, including the substrate angiotensin 2 (AngII, Sequence: DRVYIHPF) or the ACE2 inhibitor MLN-4760. The design of both libraries is similar in size to AngII. Yet, the lack of peptidomimetics discovered that are similar to AngII could be due to the libraries’ carboxamide C-terminus interfering with the substrate binding site or because of ACE2 enzymatic activity cleaving peptides closely bound to the AngII active site. Cleaved peptides would be filtered out during analysis^42^. Moreover, ACE2 receptor blockers (ARBs) also do not appear similar to the discovered binders here, though peptidomimetic ARBs have been a source of inspiration for their small-molecule design^53^. With the use of the ARB MLN-4760, competitive BLI binding experiments were able to identify that ABP N1 and C8 are partially inhibited by MLN-4760, suggesting N1 and C8 bind near the active site and could be further developed.

Natively, ACE2 is a counter-regulator of the renin-angiotensin hormonal cascade. However, elevated levels of ACE2 in plasma have been found to be the highest-ranked predictor of death, heart failure, stroke, and myocardial infarction compared to several established risk factors^47,55–59^. While this relationship is poorly understood, ACE2 plasma concentrations are a measurable indicator of renin-angiotensin system dysregulation^47^. As the target of our work here, we sought to design serum-stable, high affinity peptidomimetic ACE2 binders as the response to such a demand.

This work highlights that optimized AS-MS methods^43^ can be used for the discovery of high affinity peptidomimetics with enhanced stability from a single rapid experiment. It is challenging to use existing approaches including phage-display, one-bead-one-compound (OBOC), or mRNA-display, to achieve selections at high chemical diversity and speed simultaneously. Multiple putative ACE2 binders were discovered from a single affinity selection experiment and several of the top hits were validated to bind to ACE2 with low-nanomolar affinities. Moreover, the immediate discovery of serum-stable noncanonical peptidomimetics like ABP N1 demonstrates the potential toward acceleration of the development timeline that our AS-MS platform can provide. We envision that the reported ACE2 binders in this study may be further developed as reliable and sensitive diagnostics to detect plasma ACE2 concentrations, as well as peptide conjugates for tissue-targeting or directed delivery of therapeutics.

## Methods

### Materials

H-Rink Amide-ChemMatrix resin was obtained from PCAS BioMatrix. Tentagel M NH_2_ resin was obtained from Rapp Polymere. 4-[(*R*,*S*)-α-[1-(9*H*-Fluoren-9-yl)-methoxyformamido]-2,4-dimethoxybenzyl]-phenoxyacetic acid (Fmoc-Rink amide linker) was obtained from Chem-Impex International. Biotin-PEG4-propionic acid and Biotin-PEG4-NHS were both purchased from ChemPep Inc. *N*,*N*-Diisopropylethylamine (DIEA) was obtained from a Seca Solvent Purification System by Pure Process Technology. Peptide synthesis-grade *N*,*N*-dimethylformamide (DMF), dichloromethane (DCM), diethyl ether, and HPLC-grade acetonitrile were obtained from VWR International. 1-[Bis(dimethylamino)methylene]-1*H*-1,2,3-triazolo[4,5-b]pyridinium-3-oxid-hexafluorophosphate (HATU) was purchased from P3 BioSystems. Trifluoroacetic acid (TFA; for HPLC, ≥ 99%), piperidine (ReagentPlus; 99%), triisopropylsilane (98%), 1,2-ethanedithiol (≥ 98%), phenylsilane (97%) and tetrakis(triphenylphosphine)palladium(0) (99%), were purchased from MilliporeSigma. Biotechnology grade bovine serum albumin (BSA) was obtained from VWR International. Ultrapure water was obtained by filtering deionized water with a Milli-Q water purification system (Millipore). Canonical amino acid monomers Fmoc-Ala-OH, Fmoc-Arg(Pbf)-OH, Fmoc-Asn(Trt)-OH, Fmoc-Asp(*t*Bu)-OH, Fmoc-Gln(Trt)-OH, Fmoc-Glu(*t*Bu)-OH, Fmoc-Gly-OH, Fmoc-His(Trt)-OH, Fmoc-Leu-OH, Fmoc-Lys(Boc)-OH, Fmoc-Met-OH, Fmoc-Phe-OH, Fmoc-Pro-OH, Fmoc-Ser(*t*Bu)-OH, Fmoc-Thr(*t*Bu)-OH, Fmoc-Trp(Boc)-OH, Fmoc-Tyr(*t*Bu)-OH, and Fmoc-Val-OH were purchased from Advanced ChemTech (Louisville, KY). Noncanonical monomers Fmoc-3,4-difluoro-L-phenylalanine, Fmoc-β-cyclopropyl-L-alanine, Fmoc-D-4-thiazolyl-alanine, Fmoc-(4-aminomethyl) benzoic acid, Fmoc-β-cyclohexyl-L-alanine, Fmoc-L-α-aminoadipic acid δ-*tert*-butyl ester, Fmoc-D-proline, Fmoc-L-methionine sulfone, Fmoc-β-alanine-OH, Fmoc-4-phenylpiperidine-4-carboxylic acid, Fmoc-L-ornithine(Boc), Fmoc-4-(Boc-amino)-L-phenylalanine, Fmoc-3-(4′-pyridyl)-L-alanine, Fmoc-L-homoarginine(Pbf)-OH, Fmoc-hydroxyproline(*t*Bu)-OH, Fmoc-3,4-dimethoxy-L-phenylalanine, and Fmoc- β-Serine(*t*Bu)-OH were purchased from Chem-Impex International.

### Automated fast-flow synthesis of canonical peptides

Canonical peptide binders were synthesized on an automated fast-flow synthesizer^60,61^ on a scale of 0.075 mmol using H-Rink Amide-ChemMatrix resin (loading capacity 0.49 mmol/g). The reactor temperature was set at 90 °C and the monomer activation was achieved by HATU. Amide bond formation was effected in 8 s, and removal of the Fmoc groups was performed in 8 s with 40% (v/v) piperidine and 2% (v/v) formic acid in DMF. Overall cycle times were about 90 s. After completion of synthesis, resins were retrieved from the synthesizer and washed with DCM (5 × 10 mL), and dried in a desiccator under reduced pressure.

### Manual solid-phase synthesis of noncanonical peptides

Noncanonical amino-acid-containing peptides were synthesized manually in batch by standard solid-phase protocols. The synthesis was carried out at a 0.05 mmol scale on H-Rink Amide-ChemMatrix resin within Torviq syringes (size 6 mL). In brief, resins were swelled with DMF for 10–15 min before the peptide sequence assembly. Coupling steps were performed at room temperature for 30 min with Fmoc-protected amino acids (0.5 mmol, 10 equivalents) dissolved in 1.25 mL of HATU (0.38 M solution in DMF, 9.5 equivalents) and 250 μL of DIEA for activation (3 equivalents). The resin was stirred multiple times during coupling and then washed (5 × 10 mL) with DMF before deprotection with 20% (v/v) piperidine in DMF (2 × 10 mL with 5 min each time). As a final step, the resin was washed again (5 × 10 mL) with DMF to remove piperidine residuals and finish the cycle. After completing all monomer couplings, the resin was washed sequentially with DMF (5 × 10 mL) and DCM (5 × 10 mL) and then dried under reduced pressure before peptide cleavage.

### Peptide cleavage

Peptide cleavages from the resin and all side-chain deprotections were performed simultaneously with 2.5% (v/v) 1,2-ethanedithiol (EDT), 2.5% (v/v) water, and 1% (v/v) triisopropylsilane in neat TFA for 2.0 h at room temperature. Five mL of cleavage cocktail was used for 0.1 mmol peptides. The resulting cleaved solution was washed with dry ice-cold diethyl ether and followed by centrifugation at 4000 rpm for 3 min to precipitate the crude peptides. The obtained solids were briefly dried and dissolved in water/acetonitrile (50:50, v/v) before freezing and lyophilization.

### Peptide purification

Peptides with crude purity >85% were purified using a Biotage Selekt^©^ flash purification system and peptides with crude purity <85% were purified using reverse-phase high-performance liquid chromatography (HPLC). 1) *Reverse-Phase HPLC*. Crude peptides with purity below 85% were dissolved in water with 0.1% TFA and then purified by semipreparative reverse-phase HPLC using an Agilent 1260 HPLC system (Agilent Zorbax SB-C3 column: 9.4 × 250 mm, 5 μm; or Agilent Zorbax SB-C18 column: 9.4 × 250 mm, 5 μm). The gradient used was 10 to 61% acetonitrile over 60 min with a 4.0 mL/min flow rate. HPLC fractions were analyzed by LC-MS and pure fractions were combined and lyophilized. 2) *Flash chromatography purification*. Synthetic peptides with the estimated LC-MS crude purity above 85% were purified with a Biotage Selekt^©^ instrument using Biotage Sfär C18 D column (Duo 100 Å 30 μm, 12 g) under a 5–60% acetonitrile gradient over 12 column volumes, and the flow rate was set to 12 mL/min. Fractions were collected based on absorbance at 214 nm and then subjected to LC-MS analysis before combining the pure fractions and lyophilization.

### Analytical high-performance liquid chromatography (HPLC)

Analytical HPLC was performed on an Agilent 1200 series system with UV detection at 280 nm. The column used was Phenomenex Kinetex (100 × 2.1 mm, 2.6 μm, 100 Å silica) with a flow rate of 0.375 mL/min. Solvents used are acetonitrile with 0.08% TFA additive (solvent B) and water with 0.1% TFA additive (solvent A). A linear gradient: 2 min hold 2% B, 2–32% B gradient from 2 to 17 min, 32–65% B gradient from 17 to 17.5 min, hold 65% B from 17.5 to 19 min. A final 6 min hold was performed with 2% B. The total method time was 25 min. HPLC purities were determined by manual integration of all signals in the area of 2–13 min.

### LC-MS analysis

Crude synthetic peptides or peptide fractions from the purification steps were analyzed by LC-MS (Agilent 6545 or 6550 ESI Q-TOF) using an Agilent Zorbax 300SB-C3 or Phenomenex Luna C18 column. Total ion chromatograms and integrated MS over the main peak are provided in the supplementary information.

#### Condition 1

Analysis was performed on an Agilent 1290 Infinity HPLC coupled to an Agilent 6545 ESI Q-TOF mass spectrometer. MS was run in positive ionization mode, extended dynamic range (2 GHz), and standard mass range (m/z in the range of 300–3000 a.m.u.). The solvent mixtures used for LC-MS chromatography were: *A* = water + 0.1% formic acid (LC-MS grade), B = acetonitrile + 0.1% formic acid (LC-MS grade). The following conditions were used for peptide analysis. Column: Zorbax 300-SB C3 (5 µm, 150 × 2.1 mm, 300 Å silica); Flow Rate: 0.8 mL/min; Gradient: 1% B 0–2 min, linearly ramp from 1% B to 61% B 2 to 11 min, 61% B to 95% B 11 to 12 min. Post time is 1% B for 3 min. MS data were acquired from 4 to 11 min.

#### Condition 2

Analysis was performed on an Agilent 1290 Infinity HPLC coupled to an Agilent 6550 Q-TOF with Dual Jet Stream ESI ionization and iFunnel. MS was run in positive ionization mode, extended dynamic range (2 GHz), and low mass range (m/z in the range of 100–1700 a.m.u.). The solvent mixtures used were as above. Column: Phenomenex Luna C18 (3 µm, 150 × 1 mm, 100 Å silica); Flow Rate: 0.5 mL/min; Gradient: 1% B 0–2 min, linearly ramp from 1% B to 61% B 2 to 14 min, 61% B. Post time is 1% B for 3 min. MS data were acquired from 4 to 14 min.

### Split-and-pool synthesis of peptide libraries

Split-and-pool synthesis was carried out on a 30 μm TentaGel resin (0.26 mmol/g) for a 10^8^-member library. Each coupling step was carried out as follows: solutions of Fmoc-protected amino acids, HATU (0.38 M in DMF; 0.9 eq. relative to an amino acid), and DIEA (1.1 eq. for histidine; 3 eq. for all other amino acids) were each added to individual portions of resin. Couplings were allowed to proceed for 20 min, and resin portions were recombined and washed with DMF for deprotection. Fmoc removal step was carried out by treatment of the resin with 20% piperidine in DMF (1 × flow wash; 2 × 5 min batch treatments). The resin was washed again with DMF before splitting. For the canonical library preparation, splitting was performed by suspending the resin in DMF and evenly dividing among 18 plastic fritted syringes on a manifold (18 canonical amino acids except for isoleucine and cysteine). For the noncanonical library preparation, 21 plastic fritted syringes were used (4 canonical amino acids and 17 non-proteogenic amino acids). The cycle was then continued with the next coupling until the completion of all random positions.

### Affinity selection from both canonical and noncanonical libraries

HEK293 cell-expressed biotinylated ACE2 was obtained from AcroBiosystems (Catalog: AC2-H82E6). ACE2 protein was immobilized onto streptavidin-coated magnetic beads (Thermo Fisher, Catalog number 65001) in the presence of 10% FBS in 1 × PBS on a rotating mixer for 30 min at 4 °C. Subsequently, functionalized beads were washed with 1 × PBS (3 × 1 mL) and then incubated with peptide library (typically, the concentration was at 10–20 pM/member and the reaction volume was 1.0 mL 1 × PBS containing 10% FBS) on a rotating mixer for 1.0 h at 4 °C. The beads were then washed with PBS (3 × 1 mL) on a magnetic separation rack. Bound peptides were eluted with 200 μL of 6 M guanidine hydrochloride, 200 mM phosphate, pH 6.8. Elution volume was concentrated using C18 ZipTip^®^ pipette tips. After lyophilization, powders were resuspended in water (0.1% formic acid) and submitted for nLC-MS/MS analysis.

### Nano LC-MS/MS (nLC-MS/MS) sequencing

Peptide sequence analysis was performed on an EASY-nLC 1200 (Thermo Fisher Scientific) nano-liquid chromatography handling system with an Orbitrap Fusion Lumos Tribrid Mass Spectrometer (Thermo Fisher Scientific) as previously described^43^. Samples prepared from the library selection steps were run on a PepMap RSLC C18 column (2 μm particle size, 15 cm × 50 μm ID; Thermo Fisher Scientific, P/N ES801). A nanoViper Trap Column (C18, 3 μm particle size, 100 Å pore size, 20 mm × 75 μm ID; Thermo Fisher Scientific, P/N 164946) was used for desalting. The standard nano-LC method was run at 40 °C and a flow rate of 300 nL/min with the following gradient: 1% solvent B in solvent A ramping linearly to 61% B in A over 60 or 90 min, where solvent A = water (0.1% FA), and solvent B = 80% acetonitrile, 20% water (0.1% FA). Positive ion spray voltage was set to 2200 V. Orbitrap detection was used for primary MS, with the following parameters: resolution = 120,000; quadrupole isolation; scan range = 200–1400 m/z; RF lens = 30%; AGC target = 1 × 10^6^; maximum injection time = 100 ms; 1 microscan. Secondary MS spectra acquisition was done in a data-dependent manner: dynamic exclusion was employed such that a precursor was excluded for 30 s if it was detected four or more times within 30 s (mass tolerance: 10.00 ppm); monoisotopic precursor selection used to select for peptides; intensity threshold was set to 5 × 10^4^; charge states 2–10 were selected; and precursor selection range was set to 200–1400 m/z. The top 15 most intense precursors that met the preceding criteria were subjected to subsequent fragmentation. Three fragmentation modes collision-induced dissociation (CID), higher-energy collisional dissociation (HCD), and electron-transfer/higher-energy collisional dissociation (EThcD) were used for the acquisition of secondary MS spectra. Only precursors with charge states 3 and above were subjected to all three fragmentation modes; precursors with charge states of 2 were subjected to CID and HCD only. For all three modes, the detection was performed in the Orbitrap (resolution = 30,000; quadrupole isolation; isolation window = 1.3 m/z; AGC target = 2 × 10^4^; maximum injection time = 100 ms; 1 microscan). For CID and HCD, a collision energy of 30 and 25% was used, respectively. For EthcD, a supplemental activation collision energy of 25% was used.

### Bio-layer interferometry

Peptide binding validation was carried out using the ForteBio Octet RED96 system. The chamber temperature was kept constant at 30 °C with a plate agitation speed at 1000 rpm. Briefly, streptavidin (SA)-coated biosensor tips were dipped into 200 μL of 1.0–2.0 μM biotinylated peptide solution (in a kinetic buffer (K.B.): 1 × PBS with 0.1% BSA and 0.02% Tween-20) for peptides immobilization. Once loaded with peptides, the tips were then moved into solutions containing various concentrations (1000, 500, 250, 125, and 62.5 nM) of recombinant ACE2 protein (purchased from Sino Biological, Catalog number 10108-H08H) in the K.B. to obtain the association curve. After the 180 s association step, the tips were moved back into the K.B. to obtain the dissociation curve. Buffer-only and protein-only conditions (concentration at 1000 nM) were used as references for background subtraction. The association and dissociation curves were fitted with the ForteBio Biosystems Data Analysis Software under 5 experimental conditions (*n* = 5, global fitting algorithm, binding model 1:1) to calculate the apparent dissociation constant (*K*_D_).

### Serum stability study

Following our previous protocol^62^, the normal human serum (NHS) (Sigma Catalog number H4522) was thawed, centrifuged at 14,000 rpm for 10 min and the supernatant was pre-warmed in a 37 °C water bath. 500 µL of tested peptides were placed in a 1.7 mL Eppendorf tube. Five hundred µL of either 10% NHS (diluted in 1 × PBS) or 1 × PBS for the control, was added into the tube (5% NHS after dilution), and immediately the tubes were vortexed. Assay tubes were placed at 37 °C water bath for 3.0 h, and 200 µL were transferred to a fresh microfuge tube with 40 µL of 15% trichloroacetic acid (TCA). Tubes were then placed on ice for 15 min and centrifuged at 14,000 rpm for 10 min. Supernatant from each tube was collected and subjected to LC-MS analysis.

### ACE2 pulldown from human serum

Following a previous protocol^44^, streptavidin-coated magnetic beads (100 µL, at 10 mg/mL) were pre-washed with blocking buffer (1 × PBS with 0.05% Tween-20, pH 7.4) and captured on a magnetic separation rack. The beads were then re-suspended in 1.0 mL of the same blocking buffer. Biotinylated ABP N1 peptide (25 µL, 0.1 mM) was then added to the beads. After 30 min of incubation at 4 °C, the supernatant was removed and the beads were washed with 1 × PBS (3 × 1.0 mL) before incubation with the protein mixture. Soluble ACE2 (5 µL, 0.62 mg/mL) was mixed with 50 µL normal human serum in 1 × PBS (pH 7.4) to a final volume of 1.0 mL. The magnetic beads displaying immobilized ABP N1 were then added into the protein complex. After 1.0 h incubation at 4 °C with gentle rotation, the supernatant was removed and the beads were washed with 1 × PBS (3 × 1.0 mL) and captured on a magnetic separation rack. The captured protein was eluted with 20 µL of 6 M urea solution, followed by SDS-PAGE gel analysis. A precast polyacrylamide gel, Bolt^TM^ Bis-Tris Plus gel (Invitrogen, Catalog number NW04120BOX), was used for an optimal separation under denaturing conditions with a running voltage of 165 V for 36 min. The SeeBlue^TM^ Plus2 pre-stained protein standard (Thermo Fisher, Catalog number LC5925) was used as the molecular weight reference, and Bolt^TM^ LDS Sample Buffer (Thermo Fisher, Catalog number B0007) was used to prepare and load the protein samples.

### Enzyme-linked immunosorbent assay (ELISA)

A serial dilution of soluble ACE2 was coated onto an ELISA plate (96-well format) overnight at 4 °C. The next day, the plate was blocked with 5% BSA in 1 × PBS supplement with 0.05% Tween-20 (PBST, pH 7.4) for 2–3 h at room temperature. After a brief wash, 100 µL solution of biotinylated peptide ABP N1 (100 nM in PBST) was added to the wells and incubated for 1.0 h at room temperature. Control groups are biotinylated peptide ABP N8 and PBST alone. The supernatant was then removed, and the wells were washed with PBST (3× 200 µL). Streptavidin-HRP conjugate in PBST (0.25 µg/mL, 100 µL) was added to each well and incubated for 30 min at room temperature. The plate was PBST-washed again (3 × 200 µL) before color development with 100 µL 1-Step™ Ultra TMB-ELISA Substrate Solution (Thermo Fisher, Catalog number 34028). After 10 min, the reaction was quenched with 2.0 M sulfuric acid (100 µL). Finally, the absorbance at 450 nm was measured on a microplate reader (BioTek) for all treated wells. The same procedure was repeated when the ELISA plate was coated with a mixture of ACE2 and normal human serum. Three replicates were used throughout.

### Reporting summary

Further information on research design is available in the Nature Research Reporting Summary linked to this article.

## Supplementary information

Supplementary Information

Reporting Summary

Supplementary Data 1

## Data Availability

All data generated during this study are available either in the main text or supplementary materials. The raw data underlying Figs. 2a–f, 3a–d, 5b, c and Supplementary Figs. 21 and 22 are provided as source data in the Supplementary Data 1 file.
