# Supplementary material for: Rapid de novo discovery of peptidomimetic affinity reagents for human angiotensin converting enzyme 2
Source: Commun Chem. 2022 Jan 19;5:8. doi: 10.1038/s42004-022-00625-3 (PMC9814530; doi:10.1038/s42004-022-00625-3)
Supplement: Supplementary file 1 — Supplementary Information [file 42004_2022_625_MOESM1_ESM.pdf]

Supplementary Information for

Rapid *de novo* discovery of peptidomimetic affinity reagents for human angiotensin converting enzyme 2

Authors: Genwei Zhang[1†], Joseph S. Brown[1†], Anthony J. Quartararo[1#], Chengxi Li[1], Xuyu Tan[1], Stephanie Hanna[1], Sarah Antilla[1], Amanda E. Cowfer[1], Andrei Loas[1], Bradley L. Pentelute[1-4,*]

Affiliations:

[1]Massachusetts Institute of Technology, Department of Chemistry, 77 Massachusetts Avenue, Cambridge, MA 02139, USA

[2]The Koch Institute for Integrative Cancer Research, Massachusetts Institute of Technology, 500 Main Street, Cambridge, MA 02142, USA

[3]Center for Environmental Health Sciences, Massachusetts Institute of Technology, 77 Massachusetts Avenue, Cambridge, MA 02139, USA

[4]Broad Institute of MIT and Harvard, 415 Main Street, Cambridge, MA 02142, USA

[†]These authors contributed equally to this work.

[#]Current address: FogPharma, 30 Acorn Park Dr, Cambridge, MA 02140, USA.

*Correspondence to: blp@mit.edu

CONTENTS

SUPPLEMENTARY NOTE 1: LIBRARY SELECTION USING AS-MS 3

1.1 SUMMARY OF THE DISCOVERED HITS 3

1.2 EXTRACTED ION COUNTS OF REPRESENTATIVE PEPTIDES. 8

SUPPLEMENTARY NOTE 2: RAW LC-MS DATA 17

2.1 LC-MS TRACES OF PURIFIED NONCANONICAL PEPTIDES 17

2.2 LC-MS TRACES OF PURIFIED CANONICAL PEPTIDES 24

SUPPLEMENTARY NOTE 3: ANALYTICAL HPLC DATA 40

3.1 HPLC TRACE OF NONCANONICAL PEPTIDES (BIOTINYLATED) 40

3.2 HPLC TRACE OF NONCANONICAL PEPTIDES (NONBIOTINYLATED) 42

3.3 HPLC TRACE OF CANONICAL PEPTIDES (BIOTINYLATED) 43

3.4 HPLC TRACE OF CANONICAL PEPTIDES (NON-BIOTINYLATED) 48

SUPPLEMENTARY NOTE 4: BINDING AFFINITY MEASUREMENTS 52

4.1 BLI RAW BINDING DATA OF NONCANONICAL PEPTIDES 52

4.2 BLI RAW BINDING DATA OF CANONICAL PEPTIDES 53

4.3 THE BINDING OF SCRAMBLED ABP N1 PEPTIDES TO ACE2 54

4.4 THE BINDING OF ABP NONCANONICAL PEPTIDES TO AN UNRELATED PROTEIN 54

SUPPLEMENTARY NOTE 5: BINDING COMPETITION OF SARS-COV-2 RBD-ACE2 INTERACTION 55

SUPPLEMENTARY NOTE 6: BINDING COMPETITION OF KNOWN ACE2 INHIBITORS TO ABPS-ACE2 INTERACTION 58

Supplementary Note 1: Library selection using AS-MS

1.1 Summary of the discovered hits

Supplementary Table 1. The summary of identified canonical and noncanonical library selection hits.

Library	ACE2 outcome
Noncanonical L-library (**Library 2**)<br>2 x 10^8, $X_{12}K$	**From BLI:** 3 of 5 binders synthetized were true binders<br>**From AS-MS:** 48 EIC selective binders,<br>6 obscured by MS baseline,<br>2 nonspecific binders
Canonical L-library (**Library 1**)<br>2 x 10^8, $X_{12}K$	**From BLI:** 7 of 9 binders synthetized were true binders<br>**From AS-MS:** 60 EIC selective binders,<br>2 nonspecific binders

Supplementary Table 2. All AS-MS discovered canonical L-peptides from **Library 1**.

Name	Sequence	ALC	m/z	z	RT, min	Calc Mass, Da	Error, ppm	EIC Specific?
ABP C1	L F R G Y F A A M N N L K	99	515.2775	3	55.7	1542.8130	-1.6	Yes
ABP C8	L Q W H P A Y W F M Q V K	99	578.2968	3	68.9	1731.8708	-1.3	Yes
	L V K G D Q Y G W V W G K	99	512.2767	3	49.7	1533.8093	-0.7	Yes
	L V K S L N S Y F F V F K	99	530.9725	3	67.4	1589.8970	-0.9	Yes
	V P K N F S W N L W R P K	99	418.4868	4	49.6	1669.9204	-1.3	Yes
	W T Y D L F S M N F G R K	99	555.2733	3	68.2	1662.7976	0.2	Yes
	Y N K G P F K N G H L F K	99	387.9659	4	24.6	1547.8362	-1.2	Yes
ABP C9	L Q W M N K Y T N Y G L K	98	553.2873	3	50.4	1656.8447	-2.8	Yes
	L W T V H W F Q T Y T P K	98	569.2995	3	63.0	1704.8777	-0.6	Yes
	N P W W P L P Y H G M Y K	98	563.2769	3	64.1	1686.8130	-2.4	Yes
	Y N V G L Y T S M E K Y K	98	532.2693	3	46.5	1593.7861	-0.1	Yes
ABP C4	L K V K Y M W D Y L F G K	98	563.9764	3	67.6	1688.9111	-2.2	Yes
	L N T T R S V L W Y W P K	96	554.9756	3	70.3	1661.9043	0.4	Yes
ABP C6	L Q K F L G E R F P G W K	96	535.6412	3	72.5	1603.8987	2.0	Yes
	L H F S R Q W Q W N V R K	95	446.7453	4	33.4	1782.9543	-1.3	Yes
ABP C7	L Q R G V F G F P Y R V K	95	392.2314	4	42.8	1564.8989	-1.4	Yes
	L T F A N H T T Q V R P K	95	378.7159	4	25.0	1510.8369	-1.2	Yes
	T H V V A H A R D A Y R K	95	381.4610	4	27.3	1521.8276	-8.5	Yes
	V Q R Q P P N Y W N G F K	95	544.9509	3	40.8	1631.8320	-0.8	Yes
	V T Y Y G S G P Q W F W K	95	809.3956	2	68.0	1616.7776	-0.6	Yes
ABP C2	L H F A K W N H V W S W K	94	435.2328	4	49.0	1736.9053	-1.7	Yes
	N K F K N W N T W V G G K	94	526.6152	3	56.6	1576.8262	-1.5	Yes
ABP C3	L H K Q F G W H W F G F K	94	429.9771	4	49.8	1715.8838	-2.7	Yes
	L P F H S A Y W Y H M E K	93	427.7078	4	45.5	1706.8027	-0.5	Yes
ABP C5	L Q A K P V P Q F W P F K	93	528.9722	3	59.7	1583.8977	-1.8	Yes
	L Q R A S L Y F P W K V K	93	409.4927	4	48.4	1633.9456	-2.3	Yes
	L A F H N K P E W Y W P K	92	429.4764	4	48.3	1713.8779	-0.9	Yes
	L R F Q Q V S F Y P W R K	92	439.2460	4	51.8	1752.9575	-1.5	Yes
	L E L S T N W V W N P Y K	91	550.2910	3	47.8	1647.8408	6.3	Yes
	T R A L S N F D F F R P K	91	400.2198	4	46.8	1596.8525	-1.5	Yes
	V Q W N M L G F Y N P W K	91	561.2819	3	62.9	1680.8235	0.3	Yes
	K A L T V R L M F Y V W K	90	418.2440	4	49.9	1668.9539	-4.2	Yes
	L Q F W N G P V H E E H K	90	540.6081	3	56.6	1618.8005	1.2	Yes
	L Q Y K P A Q M L N W P K	90	539.2975	3	51.6	1614.8704	0.3	Yes
	L V K W T Y T L N M D Q K	90	546.9651	3	64.4	1637.8599	8.4	Yes
	L V Y A W G S R D Y P W K	90	820.4226	2	77.1	1638.8306	0.1	Yes
	Y A Y Q D D L W G V F P K	90	800.8931	2	76.4	1599.7722	-0.4	Yes
	D N R G P D V S Y Y K W K	89	407.4561	4	37.7	1625.7949	0.1	Yes
	L Q R T M L L W T R P F K	89	422.9993	4	49.0	1687.9707	-1.6	Yes
	V Q W H S Y P N Y L Q Y K	89	575.6227	3	51.1	1723.8472	-0.4	Yes
	Q L F S W W T T Q Y A Y K	88	860.9265	2	77.9	1719.8408	-1.4	Yes
	V K K Y L L L N A N Y P K	88	521.6507	3	38.0	1561.9343	-2.6	Yes
	K A N N R F K N F Y E R K	86	571.9835	3	61.1	1712.9224	3.7	Yes
	T T R H T A R V H W V F K	86	410.2341	4	40.9	1636.9062	0.6	Yes
	L A F Q H G S T A P S W K	84	476.9171	3	39.2	1427.7310	-0.9	Yes
	L H F M N W A M N R G G K	84	520.9266	3	42.1	1559.7603	-1.4	Yes
	W F R L N W A T N F S R K	84	431.9831	4	56.5	1723.9060	-1.7	Yes
	W H L S T A P S F N P W K	84	523.9368	3	57.1	1568.7888	-0.2	Yes
	L A N F G E Q N E W F W K	84	556.6021	3	71.3	1666.7893	-2.9	Yes
	K W A Q D Y Y Q N M A W K	83	582.9432	3	65.3	1745.7983	5.4	Yes
	V S N R P P Y F W T R F K	83	424.9823	4	43.9	1695.8997	0.3	Yes
	W N V D P Y Y F M Q A W K	83	582.9409	3	65.5	1745.8025	-1.0	Yes
	G R F R W H A N D H H T K	82	554.2863	3	46.5	1659.8242	7.8	Yes
	L H Q Y N H K R P W G W K	82	437.9839	4	60.1	1747.9172	-6.1	Yes
	L Q F E K K M Q Q H H D K	82	571.2938	3	55.7	1710.8623	-1.6	Yes
	L V E F E Y R W R M F V K	82	451.2451	4	55.9	1800.9497	1.0	Yes
	L D F K Q M S G W L H S K	81	394.7071	4	46.2	1574.8027	-2.3	Yes
	W H R A V V S D L L F E K	81	533.6312	3	53.3	1597.8728	-0.7	Yes
	W L N N Y P W W W S T P K	80	592.9593	3	62.9	1775.8572	-0.6	Yes
	K N E L V G Y G W V W G K	74	512.2767	3	49.8	1533.8093	-0.7	Yes
	W N V G V Y H K W F R V K	96	430.2407	4	43.2	1716.9365	-1.7	No
	F W W S N P Y L R Q G D K	87	565.9498	3	65.1	1694.8318	-2.4	No

Graphical representation of sequence alignment from AS-MS with Library 1

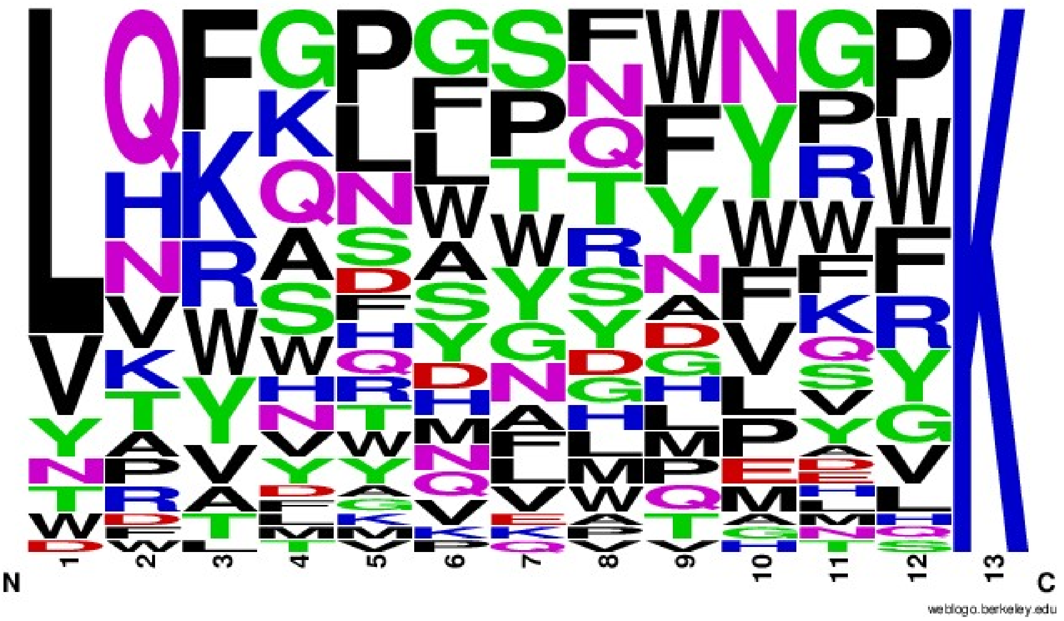

Supplementary Fig. 1. Weblogo plot of the binders discovered via AS-MS against ACE2 from the canonical L-library (2 x 10^8 members, X12K) demonstrating the presence of a modest N-terminal motif of '(L/V)(Q/N)'.

Supplementary Table 3. All AS-MS discovered noncanonical-L peptides from **Library 2**.

Name	Peptide sequence, 3 letter code													ALC (%)	m/z	z	RT, min	Calc Mass, Da	Error, ppm	EIC Selectivity
	Cpa	Tha	4Py	Aad	Cha	Cpa	Gly	bAla	hArg	Ile	Cpa	bAla	Lys	87	520.6335	3	40.9	1558.8801	-0.8	Yes
	Cpa	Tha	4Py	Cpa	Ile	Orn	Msn	D-Pro	Cpa	bAla	Ile	Tha	Lys	99	536.2723	3	39.7	1605.7976	-1.5	Yes
	Cpa	Gln	Dff	Orn	bAla	Aad	Cha	D-Pro	Gln	Dff	Gly	Cpa	Lys	96	542.6180	3	58.0	1624.8384	-3.7	Yes
	Aad	Tha	Ile	Amb	Amb	Cpa	Tha	Gly	4Af	Cha	bSer	bAla	Lys	98	544.5994	3	62.2	1630.7786	-1.4	Yes
ABP N4	Cpa	Tha	Cpa	Gly	D-Pro	D-Pro	Hyp	Dff	Dmf	Tha	bAla	Amb	Lys	99	545.5731	3	60.1	1633.7017	-2.6	Yes
	Ile	Tha	Ile	bAla	Dmf	Gly	Tha	bSer	hArg	Ile	Dff	bAla	Lys	84	551.9450	3	54.2	1652.8130	0.1	Yes
	Cpa	Gln	Gly	4Py	Cpa	Tha	D-Pro	Cha	Asp	hArg	Cpa	Cha	Lys	95	552.3120	3	59.3	1653.9172	-1.9	Yes
	Cha	Tha	Gly	Php	D-Pro	bSer	Ile	bAla	hArg	Gly	4Af	Php	Lys	84	552.6462	3	73.0	1654.9170	-0.1	Yes
	Cpa	Gln	Dff	bSer	Gly	Tha	Orn	Ile	Dmf	Gln	Gly	4Af	Lys	99	554.6065	3	38.6	1660.7993	-1.0	Yes
	Cpa	Cpa	Tha	4Py	Asp	D-Pro	Msn	Gly	Hyp	Ile	Dff	Php	Lys	87	555.5832	3	53.7	1663.7334	-3.4	Yes
	Cpa	Tha	Dff	Aad	Gly	Tha	bAla	Gly	Amb	Dff	hArg	4Py	Lys	85	559.5663	3	51.7	1675.6797	-1.6	Yes
	Cpa	Tha	Dff	Hyp	Cpa	Orn	Dff	Dff	bAla	Cpa	bAla	Amb	Lys	97	562.2561	3	65.3	1683.7488	-1.4	Yes
	Hyp	4Af	Msn	Ile	D-Pro	Amb	Amb	Amb	Amb	Php	bSer	bAla	Lys	97	562.6097	3	59.7	1684.8110	-2.3	Yes
ABP N5	Cpa	Tha	Dff	Gly	Gln	D-Pro	Msn	Cha	Gln	Tha	bAla	Tha	Lys	99	567.2306	3	63.7	1698.6736	-2.1	Yes
	Php	Orn	Gly	Cpa	Tha	D-Pro	Ile	Orn	Tha	Asp	Php	Tha	Lys	80	568.6047	3	56.5	1702.7930	-0.5	Yes
	Cpa	Tha	Dff	Aad	Cpa	Hyp	Gly	bAla	Hyp	Dff	hArg	Tha	Lys	90	570.5817	3	52.1	1708.7258	-1.6	Yes
	Cpa	Gln	4Py	Msn	Cha	bSer	Php	bSer	Ile	bSer	4Af	D-Pro	Lys	84	571.3110	3	44.4	1710.9165	-3.2	Yes
	Cpa	Tha	Dmf	bSer	bSer	Ile	Ile	bSer	Asp	Cha	Orn	Dff	Lys	99	571.6335	3	70.5	1711.8816	-1.7	Yes
	Ile	Dff	D-Pro	Asp	Aad	Amb	Dff	Gly	Gly	Amb	hArg	Php	Lys	98	573.2747	3	64.3	1716.8074	-3.0	Yes
ABP N1	Cpa	Tha	Dff	Orn	Gln	Orn	bSer	Tha	Cha	Msn	Gly	4Af	Lys	99	435.9548	4	28.7	1739.7905	-0.3	Yes
	Cpa	Gln	Dff	bAla	Gly	Ile	4Py	Dmf	Dmf	Cpa	Dff	Cpa	Lys	82	581.6208	3	58.3	1741.8486	-4.5	Yes
	Cha	Dff	bAla	Ile	Aad	Asp	4Py	Gly	Php	Cha	Ile	Msn	Lys	92	582.6428	3	80.2	1744.9070	-0.2	Yes
	Asp	D-Pro	Msn	4Af	Amb	Amb	Amb	Dff	Ile	Gly	Gln	Php	Lys	98	584.2676	3	66.0	1749.7825	-0.9	Yes
	Php	4Py	Amb	Msn	bSer	Cha	Aad	Asp	Aad	Msn	Gly	Amb	Lys	83	584.5899	3	58.3	1750.7581	-5.8	Yes
	Php	Gly	D-Pro	4Af	Tha	Gln	Dff	Cha	Gln	Ile	Gly	Php	Lys	80	584.9670	3	76.6	1751.8818	-1.6	Yes
	Cpa	Tha	Ile	Amb	Tha	Ile	Gly	Dff	bAla	Dmf	Gln	Dff	Lys	98	585.2582	3	83.6	1752.7563	-2.1	Yes
	Cpa	Tha	4Py	Orn	hArg	Asp	Php	Gly	Asp	Amb	Cha	Cha	Lys	84	586.3202	3	47.9	1755.9280	6.1	Yes
	Cpa	Tha	Dff	Gln	Cpa	Orn	Ile	bSer	Amb	4Af	Orn	Php	Lys	86	440.2345	4	46.3	1756.9084	0.3	Yes
	Ile	Tha	Ile	Ile	D-Pro	Amb	D-Pro	Dff	bSer	Dmf	Orn	Php	Lys	90	586.9777	3	76.8	1757.9177	-3.7	Yes
ABP N6	Cpa	Orn	Dff	Msn	Hyp	Tha	D-Pro	Gln	Dff	Cha	Cha	bAla	Lys	99	590.6218	3	73.1	1768.8450	-0.8	Yes
ABP N8	Cpa	Tha	hArg	Dpro	Gly	bAla	Php	Asp	Cha	Msn	Dff	Msn	Lys	98	590.9379	3	60.7	1769.7900	1.1	Yes
	Cpa	Aad	Dff	Dff	Amb	Gly	Dff	Hyp	Gln	hArg	Gly	Dff	Lys	95	597.5940	3	65.2	1789.7646	-2.5	Yes
	Asp	Msn	Gly	Msn	Amb	Dmf	bSer	D-Pro	4Af	Amb	Php	Amb	Lys	94	599.9252	3	55.5	1796.7578	-2.2	Yes
	4Af	Tha	Cpa	Amb	Dff	Amb	Cpa	D-Pro	Dmf	Amb	4Py	D-Pro	Lys	82	605.9469	3	62.4	1814.8240	-2.8	Yes
	Tha	Dff	hArg	Amb	D-Pro	Tha	Asp	Msn	Php	bAla	Php	Gly	Lys	99	606.5896	3	57.1	1816.7485	-0.9	Yes
	Amb	4Py	Asp	Tha	Aad	Amb	Dff	Msn	D-Pro	bAla	Php	4Py	Lys	86	456.1885	4	44.6	1820.7288	-2.0	Yes
	Ile	Tha	Dff	Gly	bAla	Amb	4Af	Tha	Dff	bSer	Dmf	Msn	Lys	99	609.9074	3	63.2	1826.7026	-1.2	Yes
	Php	Aad	Aad	Tha	4Af	Ile	Gly	Gln	Php	Msn	Asp	Amb	Lys	92	916.4117	2	68.2	1830.8108	-1.0	Yes
	Ile	Tha	hArg	bAla	Amb	Cha	hArg	Gly	Dff	Dmf	Amb	Tha	Lys	99	461.9810	4	58.5	1843.8977	-1.6	Yes
	Cpa	Tha	Tha	Msn	Ile	Gly	bAla	Dmf	Gln	hArg	Dmf	Php	Lys	81	623.6255	3	54.6	1867.8569	-1.2	Yes
	Cpa	Tha	Tha	Msn	Ile	Gln	Dmf	bAla	Gly	hArg	Dmf	Php	Lys	81	623.6255	3	54.7	1867.8569	-1.2	Yes
	Cha	Tha	Amb	D-Pro	Hyp	Tha	Tha	Php	Asp	4Py	Dff	Php	Lys	97	630.9304	3	74.6	1889.7688	0.2	Yes
	Dmf	Tha	hArg	Php	Aad	hArg	bAla	Ile	4Af	4Af	Msn	Gly	Lys	99	381.9974	5	28.7	1904.9541	-1.9	Yes
	Cha	Tha	Dff	bAla	Msn	Hyp	Tha	4Af	Dff	Msn	bSer	hArg	Lys	97	479.9479	4	51.6	1915.7649	-1.2	Yes
	Tha	Tha	hArg	bAla	Dmf	Php	Hyp	Php	Dff	Ile	Gly	Php	Lys	99	643.9741	3	72.3	1928.9067	-3.2	Yes
	Cha	Tha	Amb	hArg	Dff	Gly	Tha	hArg	Php	Amb	Amb	hArg	Lys	99	389.5978	5	50.4	1942.9563	-1.9	Yes
	Cpa	Tha	Dff	Msn	Tha	Orn	Dff	Gln	hArg	Amb	Amb	Php	Lys	99	490.7114	4	58.2	1958.8191	-1.3	Yes
	Php	bAla	Dff	Amb	Msn	Tha	Aad	Amb	Dmf	Asp	Dmf	Amb	Lys	83	659.2672	3	69.7	1974.7805	-0.4	Yes
	4Py	Tha	Cpa	Gly	D-Pro	bAla	Php	Orn	Cpa	Amb	D-Pro	Tha	Lys	88	516.2507	3	52.4	1545.7371	-4.4	Obscured in baseline
	Php	Gly	bAla	Hyp	Dff	bAla	Amb	Amb	4Py	Orn	Amb	Php	Lys	88	419.9605	4	47.6	1675.8149	-1.1	Obscured in baseline
	Cpa	4Py	hArg	4Af	Aad	4Py	Cpa	Cpa	4Af	4Af	Asp	Amb	Lys	86	439.4809	4	48.7	1753.9048	-5.9	Obscured in baseline
	Ile	4Af	Aad	Aad	Amb	Dff	Amb	Amb	Php	Gly	bSer	Amb	Lys	99	589.9507	3	63.6	1766.8306	-0.1	Obscured in baseline
	Tha	Tha	Cpa	Cha	bAla	4Py	Cha	Dmf	Gly	Dff	Php	D-Pro	Lys	97	607.9648	3	88.3	1820.8740	-0.7	Obscured in baseline
	Aad	Tha	Dff	bAla	Dmf	Cpa	Asp	Php	D-Pro	4Af	Ile	Tha	Lys	89	615.2768	3	70.5	1842.8069	0.9	Obscured in baseline
	Cpa	Gln	Dff	Orn	Ile	Amb	Gly	Amb	Gly	Cha	Ile	hArg	Lys	96	403.7390	4	56.6	1610.9258	0.7	No
	Cpa	Tha	4Py	Hyp	Orn	4Af	Dmf	Dmf	Cha	Cpa	bAla	Ile	Lys	80	604.3242	3	47.2	1809.9634	-7.0	No

Graphical representation of sequence alignment from AS-MS with Library 2

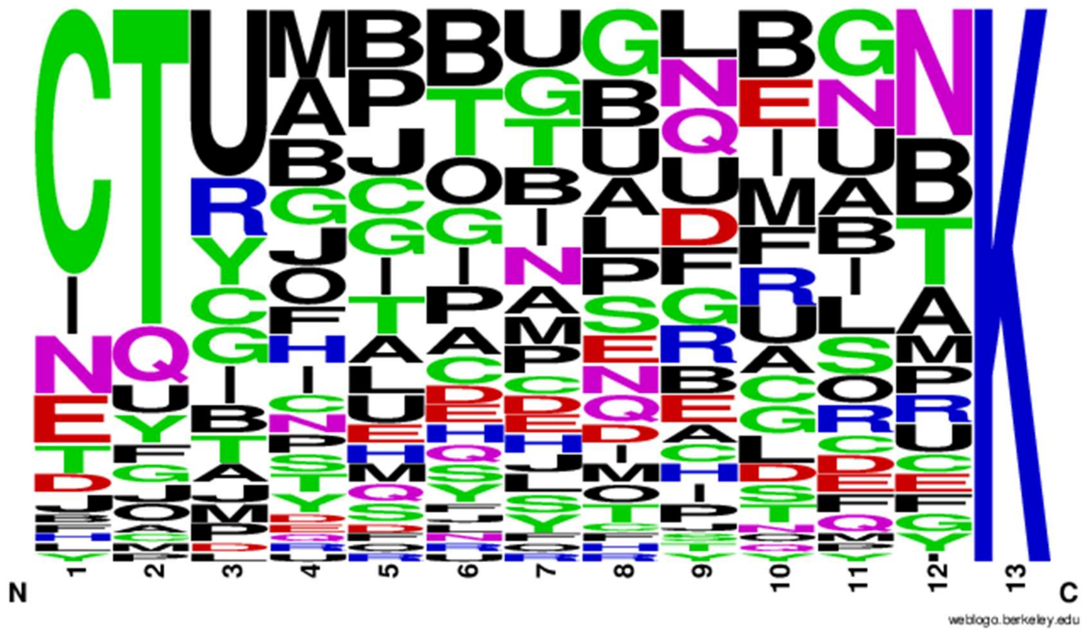

Supplementary Fig. 2. Weblogo plot of the binders discovered via AS-MS against ACE2 from the noncanonical library (2 x 10^8 members, X12K) demonstrating the presence of a modest N-terminal motif of '(C/I)(T/Q)U'. The amino acid single letter code correspondence: Gly-G; bAla-A; Amb-B; Ile-I; Cpa-C; Cha-E; D-Pro-P; Hyp-H; Gln-Q; bSer-S; Msn-M; Tha-T; 4Py-Y; 4AF-F; Asp-D; Aad-J; Orn-O; hAr-R; Dff-U; Dmf-L; Php-N; Lys-K.

1.2 Extracted ion counts of representative peptides.

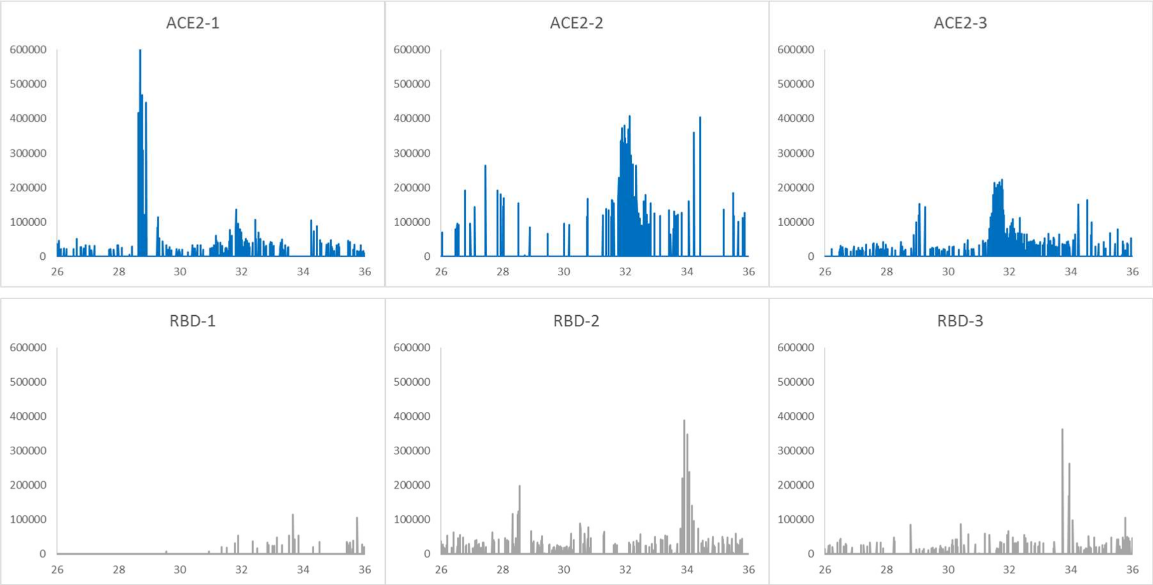

Supplementary Fig. 3. Extracted ion count (EIC) of ABP N1. ABP N1 was sequenced with an ALC of 99 from the observed 435.955 m/z, z = 4 ion, and retention time of 29 minutes for a calculated parent mass of 1739.791, meaning the observed error was -0.3 ppm. An EIC was performed from 435.95 - 435.96 m/z and demonstrates that ABP N1 showed ACE2 specificity in the library selection

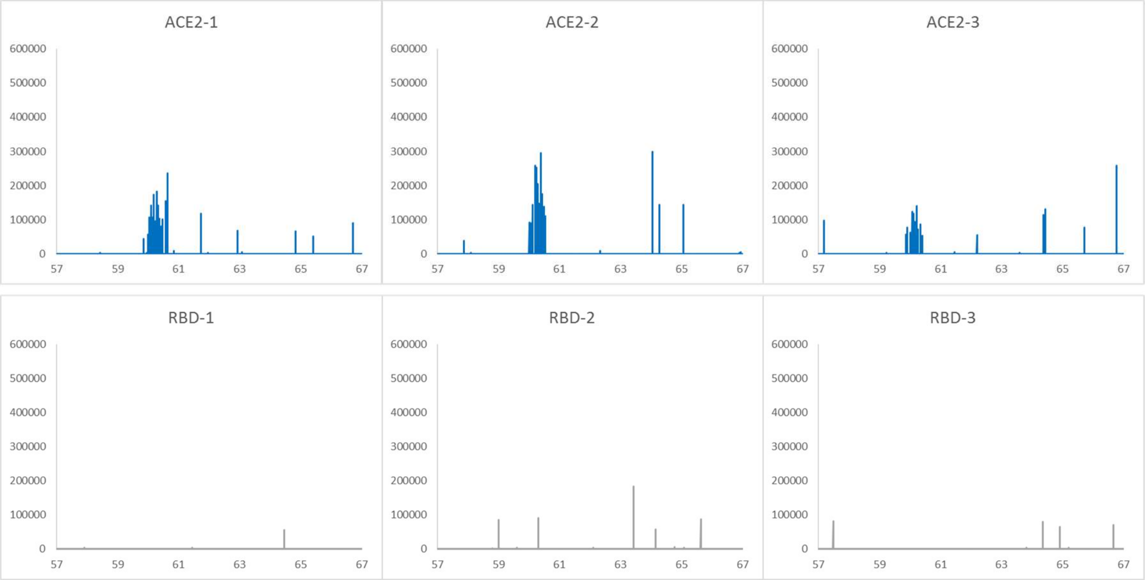

Supplementary Fig. 4. EIC of ABP N4. ABP N4 was sequenced with an ALC of 99 from the observed 545.574 m/z, z = 3 ion, and retention time of 60 minutes for a calculated parent mass of 1633.7017, meaning the observed error was -1.9

ppm. An EIC was performed from 545.57 - 545.58 m/z and demonstrates that ABP N4 showed ACE2 specificity in the library selection.

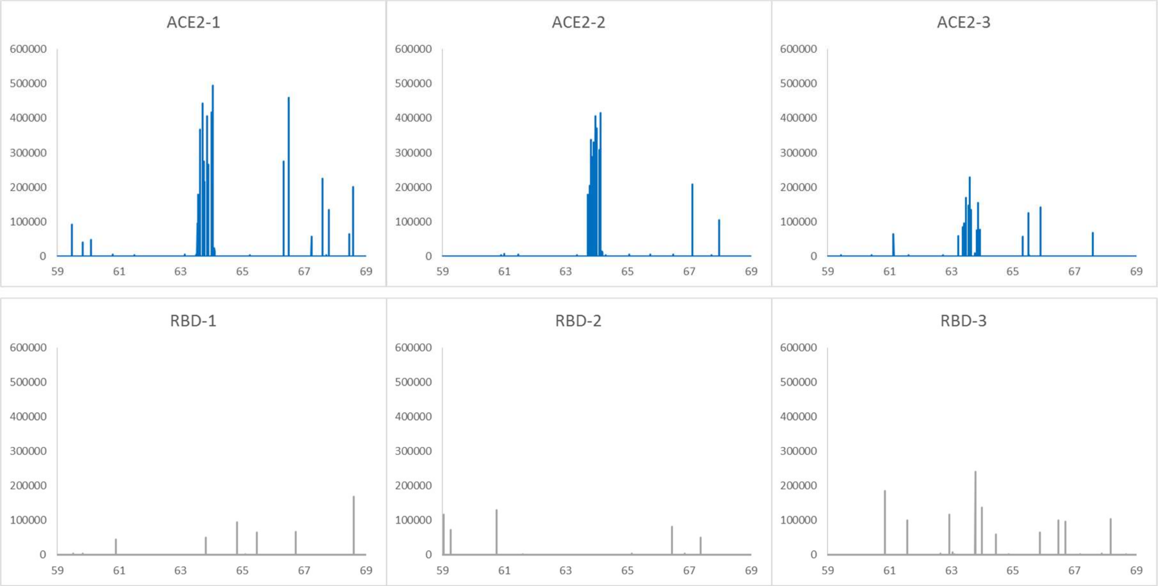

Supplementary Fig. 5. EIC of ABP N5. ABP N5 was sequenced with an ALC of 99 from the observed 567.231 m/z, z = 3 ion, and retention time of 64 minutes for a calculated parent mass of 1698.6740, meaning the observed error was -2.1 ppm. An EIC was performed from 567.23 – 567.24 m/z and demonstrates that ABP N5 showed ACE2 specificity in the library selection.

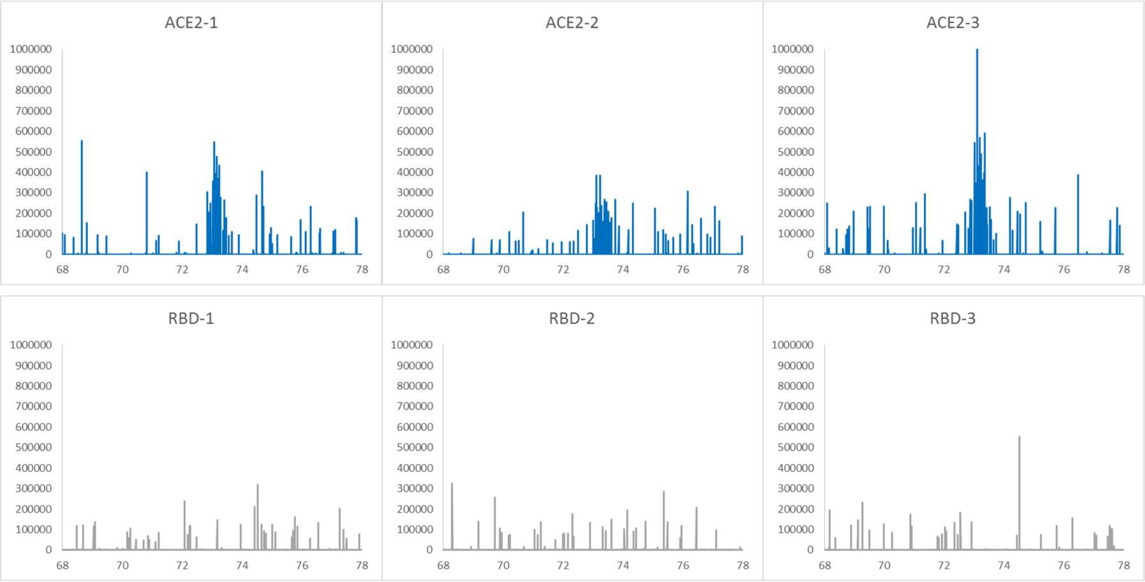

Supplementary Fig. 6. EIC of ABP N6. ABP N6 was sequenced with an ALC of 99 from the observed 590.622 m/z, z = 3 ion, and retention time of 73 minutes for a calculated parent mass of 1768.845, meaning the observed error was -0.8 ppm. An EIC was performed from 590.62 - 590.63 m/z and demonstrates that ABP N6 showed ACE2 specificity in the library selection.

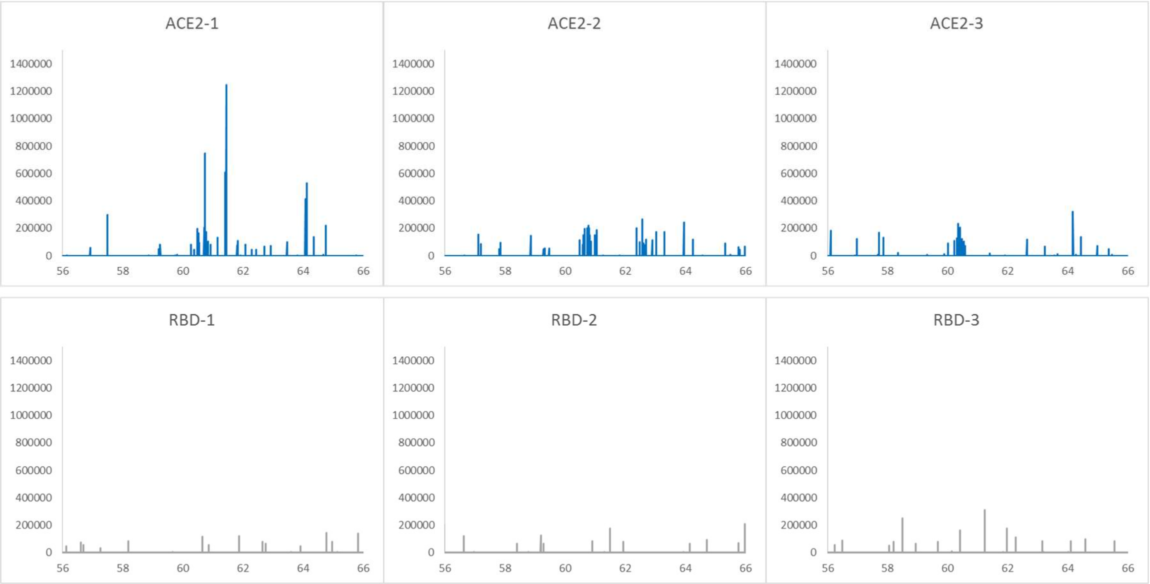

Supplementary Fig. 7. EIC of ABP N8. ABP N8 was sequenced with an ALC of 98 from the observed 590.9379 m/z, z = 3 ion, and retention time of 61 minutes for a calculated parent mass of 1769.7900, meaning the observed error was 1.1 ppm. An EIC was performed from 590.93 - 590.94 m/z and demonstrates that ABP N8 showed ACE2 specificity in the library selection.

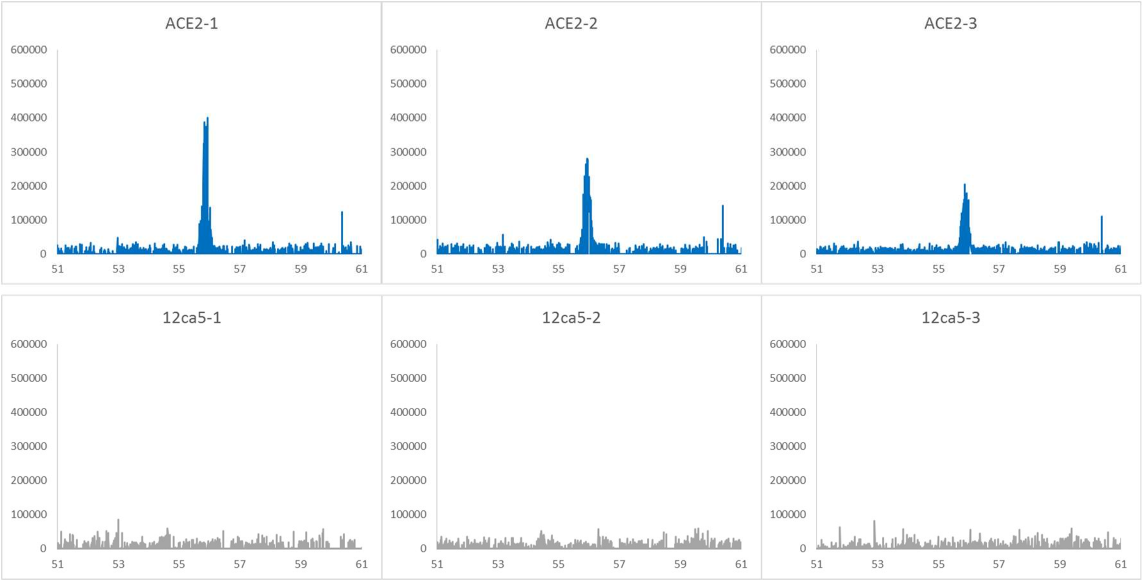

Supplementary Fig. 8. EIC of ABP C1. ABP C1 was sequenced with an ALC of 99 from the observed 515.2775 m/z, z = 3 ion, and retention time of 56 minutes for a calculated parent mass of 1542.8130, meaning the observed error was -1.6 ppm. An EIC was performed from 515.27 - 515.28 m/z and demonstrates that ABP C1 showed ACE2 specificity in the library selection.

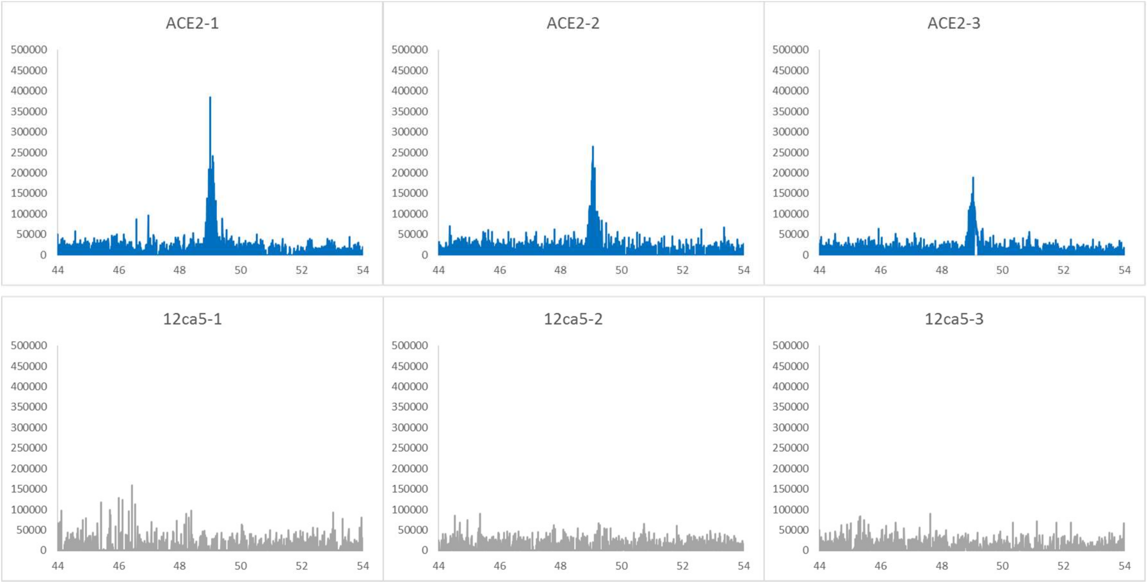

Supplementary Fig. 9. EIC of ABP C2. ABP C2 was sequenced with an ALC of 94 from the observed 435.2328 m/z, z = 4 ion, and retention time of 49 minutes for a calculated parent mass of 1736.9053, meaning the observed error was -1.7 ppm. An EIC was performed from 435.23 - 435.24 m/z and demonstrates that ABP C2 showed ACE2 specificity in the library selection.

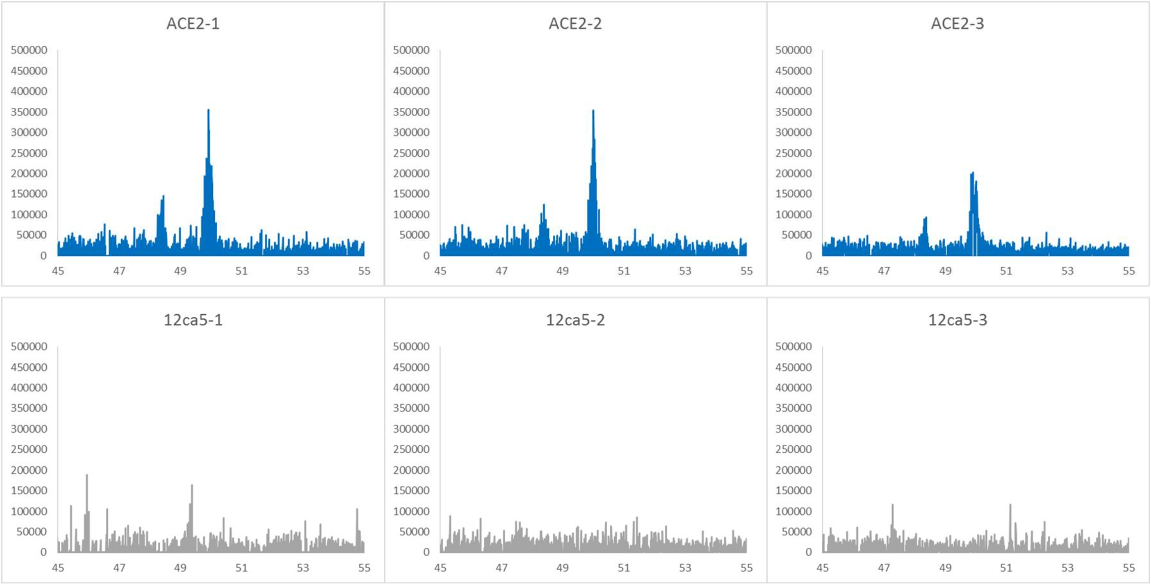

Supplementary Fig. 10. EIC of ABP C3. ABP C3 was sequenced with an ALC of 94 from the observed 429.9771 m/z, z = 4 ion, and retention time of 50 minutes for a calculated parent mass of 1715.8838, meaning the observed error was -2.7 ppm. An EIC was performed from 429.97 - 429.98 m/z and demonstrates that ABP C3 showed ACE2 specificity in the library selection.

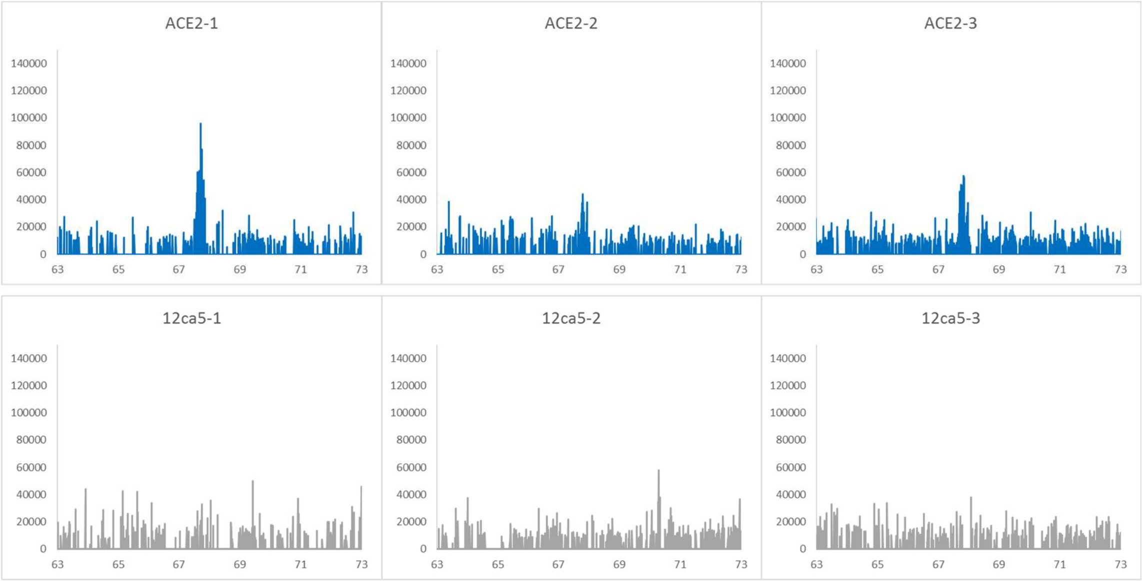

Supplementary Fig. 11. EIC of ABP C4. ABP C4 was sequenced with an ALC of 94 from the observed 563.9764 m/z, z = 3 ion, and retention time of 68 minutes for a calculated parent mass of 1688.9111, meaning the observed error was -2.2 ppm. An EIC was performed from 563.97 - 563.98 m/z and demonstrates that ABP C4 showed ACE2 specificity in the library selection.

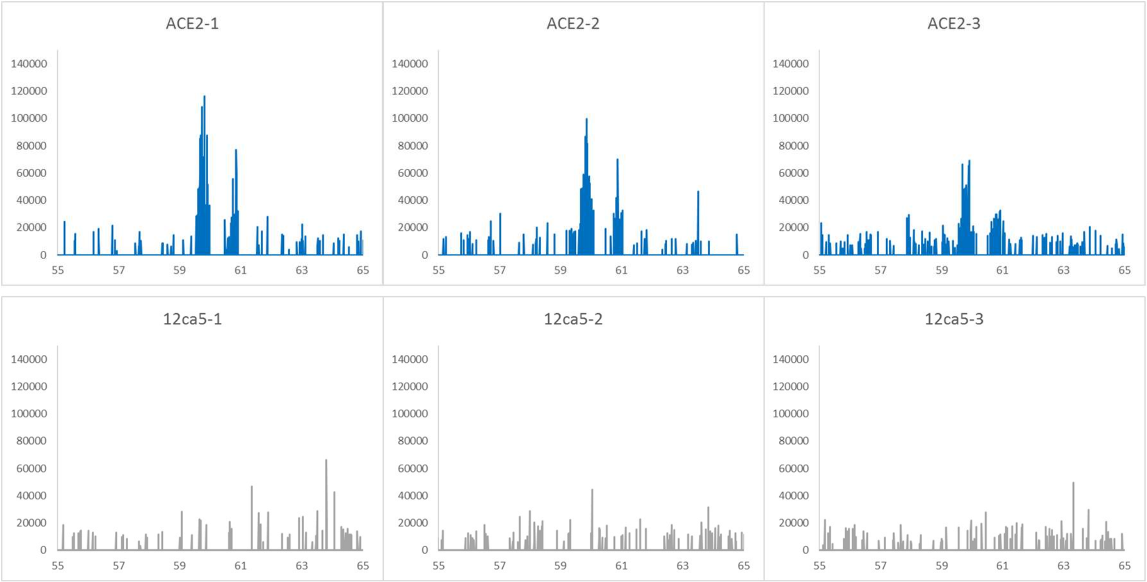

Supplementary Fig. 12. EIC of ABP C5. ABP C5 was sequenced with an ALC of 93 from the observed 528.9722 m/z, z = 3 ion, and retention time of 60 minutes for a calculated parent mass of 1583.8977, meaning the observed error was -1.8 ppm. An EIC was performed from 528.97 - 528.98 m/z and demonstrates that ABP C5 showed ACE2 specificity in the library selection.

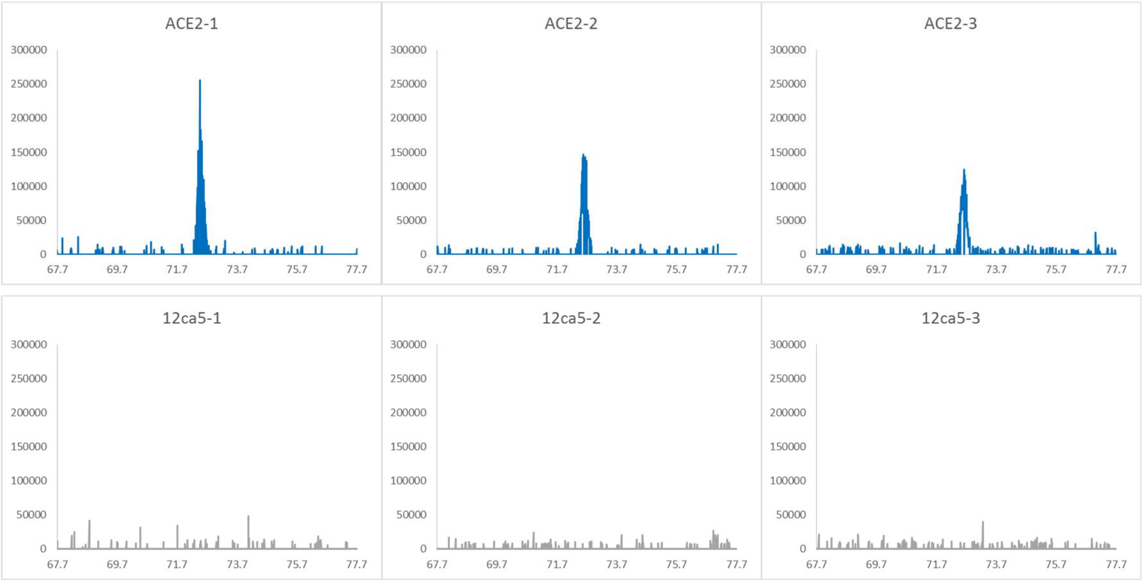

Supplementary Fig. 13. EIC of ABP C6. ABP C6 was sequenced with an ALC of 96 from the observed 535.6412 m/z, z = 3 ion, and retention time of 73 minutes for a calculated parent mass of 1603.8987, meaning the observed error was 2.0 ppm. An EIC was performed from 535.64 - 535.65 m/z and demonstrates that ABP C6 showed ACE2 specificity in the library selection.

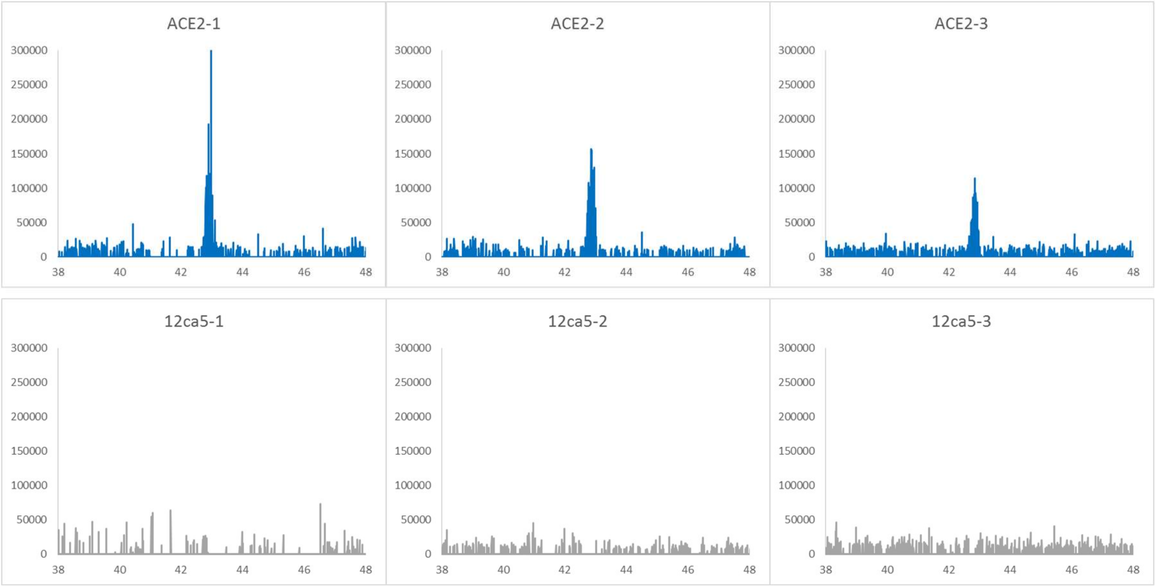

Supplementary Fig. 14. EIC of ABP C7. ABP C7 was sequenced with an ALC of 95 from the observed 392.2314 m/z, z = 4 ion, and retention time of 43 minutes for a calculated parent mass of 1564.8989, meaning the observed error was -1.4 ppm. An EIC was performed from 392.23 - 392.24 m/z and demonstrates that ABP C7 showed ACE2 specificity in the library selection.

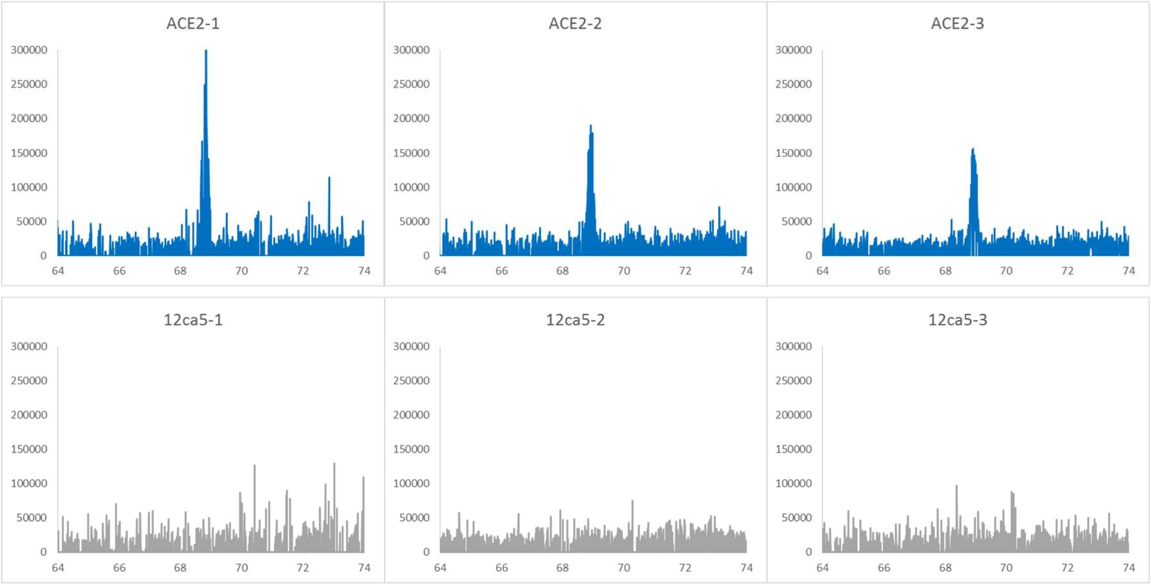

Supplementary Fig. 15. EIC of ABP C8. ABP C8 was sequenced with an ALC of 99 from the observed 578.2968 m/z, z = 3 ion, and retention time of 69 minutes for a calculated parent mass of 1731.8708, meaning the observed error was -1.3 ppm. An EIC was performed from 578.29 - 578.30 m/z and demonstrates that ABP C8 showed ACE2 specificity in the library selection.

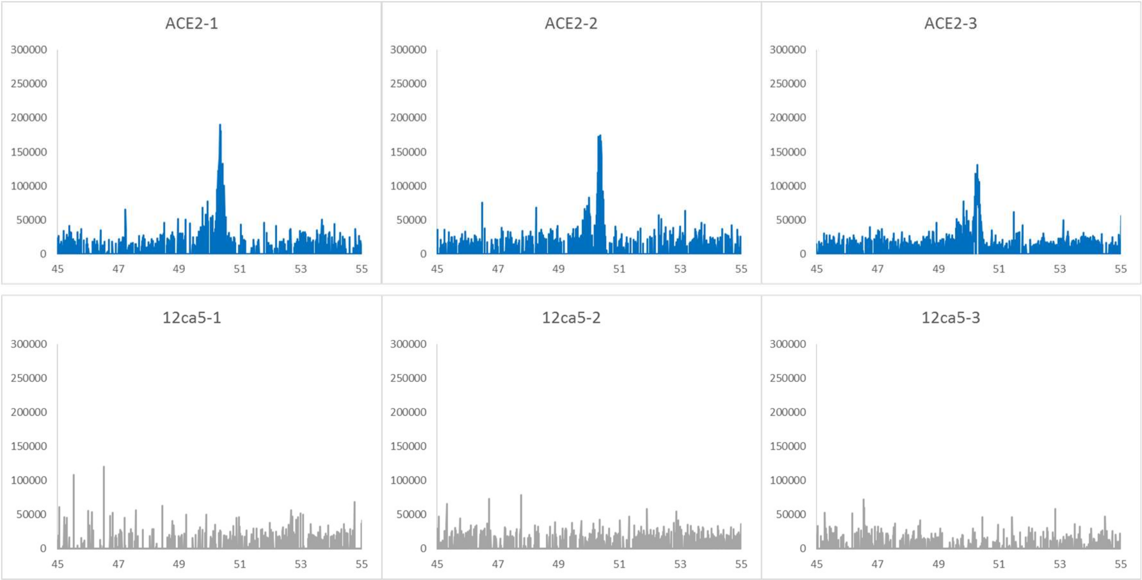

Supplementary Fig. 16. EIC of ABP C9. ABP C9 was sequenced with an ALC of 98 from the observed 553.2873 m/z, z = 3 ion, and retention time of 50 minutes for a calculated parent mass of 1656.8447, meaning the observed error was -1.3 ppm. An EIC was performed from 553.28 - 553.29 m/z and demonstrates that ABP C9 showed ACE2 specificity in the library selection.

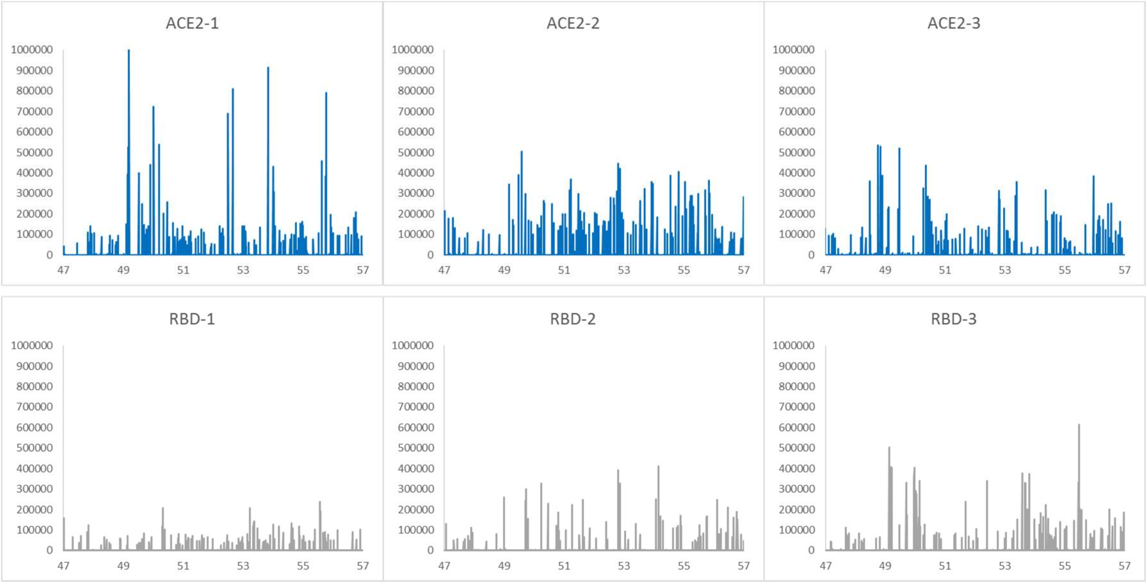

Supplementary Fig. 17. EIC of ‘4Py, Tha, Cpa, Gly, D-Pro, bAla, Php, Orn, Cpa, Amb, D-Pro, Tha, Lys’ that was sequenced, identified by PEAKS, and filtered through the standard library selection protocol. However, the EIC of this noncanonical peptide does not reveal convincing or distinct peaks that match its observed retention time of 52 minutes for an observed 516.2507 m/z and z = 3

ion. Thus, this noncanonical peptide serves as a representative example considered to be ‘obscured’ in the baseline of the mass chromatogram.

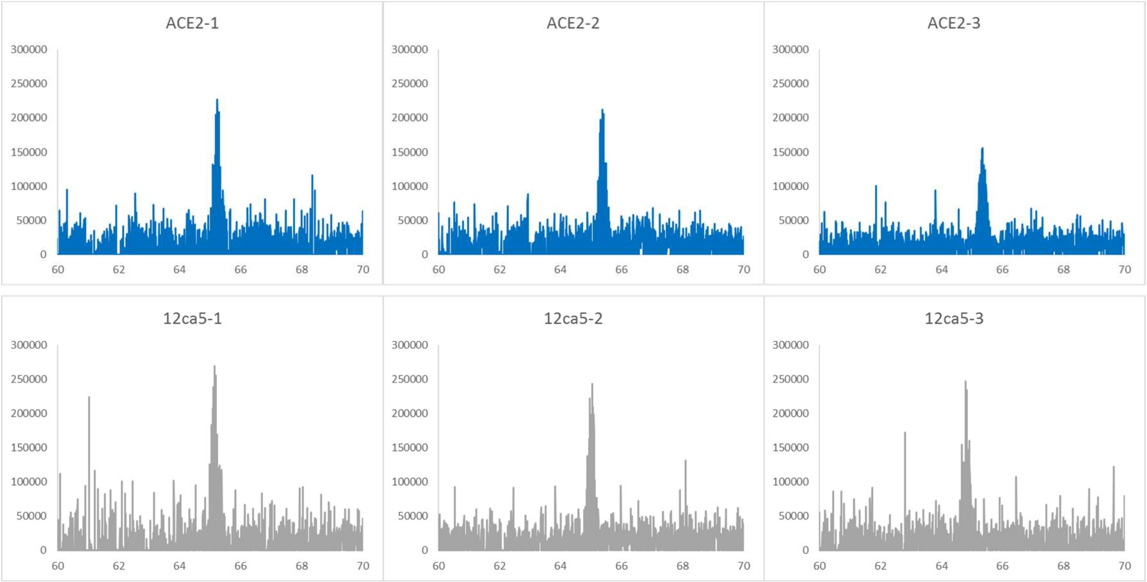

Supplementary Fig. 18. EIC of FWWSNPYLRQGDK that was sequenced, assigned in PEAKS, and filtered through the standard library selection protocol. This peptide was sequenced with an ALC of 87, observed as 565.9498 m/z, z = 3 at a retention time of 65 for a calculated mass of 1694.8318 Da, meaning the observed error was -2.4 ppm. However, the EIC of this peptide revealed that it nonspecifically binds to the anti-hemagglutinin monoclonal antibody clone 12ca5 in similar abundance as it does to ACE2. Therefore, this peptide is considered to be ‘nonspecific’.

Supplementary Note 2: Raw LC-MS data

Note: After synthesis and purification, peptide purity was assessed under either **condition 1** or **condition 2** specified in the Methods section. The total ion chromatograph (TIC) was analyzed using software Agilent MassHunter Qualitative Analysis v10.0 and the MS spectrum was extracted over the main product peaks.

2.1 LC-MS traces of purified noncanonical peptides

2.1.1 Biotin-ABP N1

Observed: 2237.95 Da; Calculated: 2238.02 Da

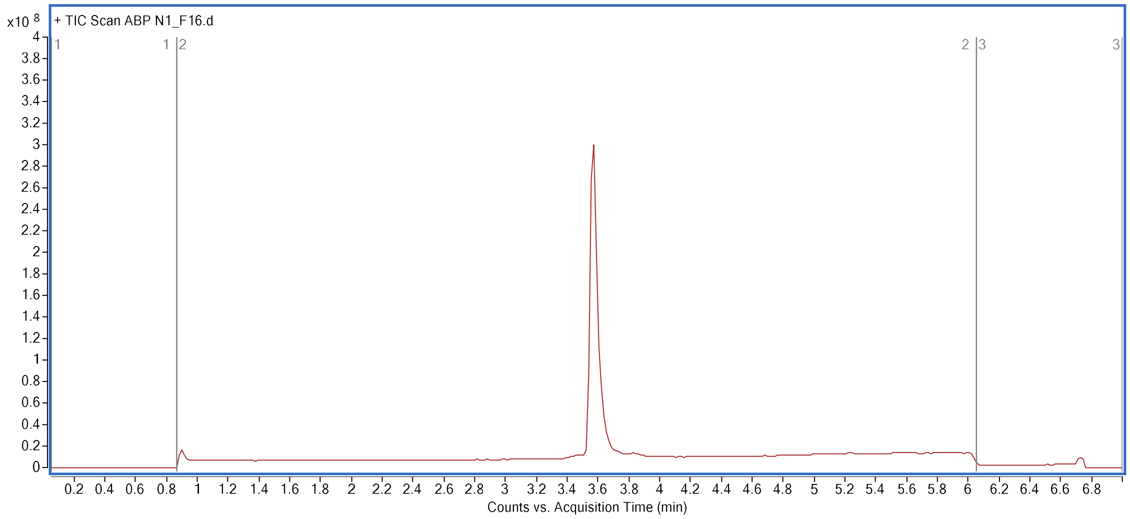

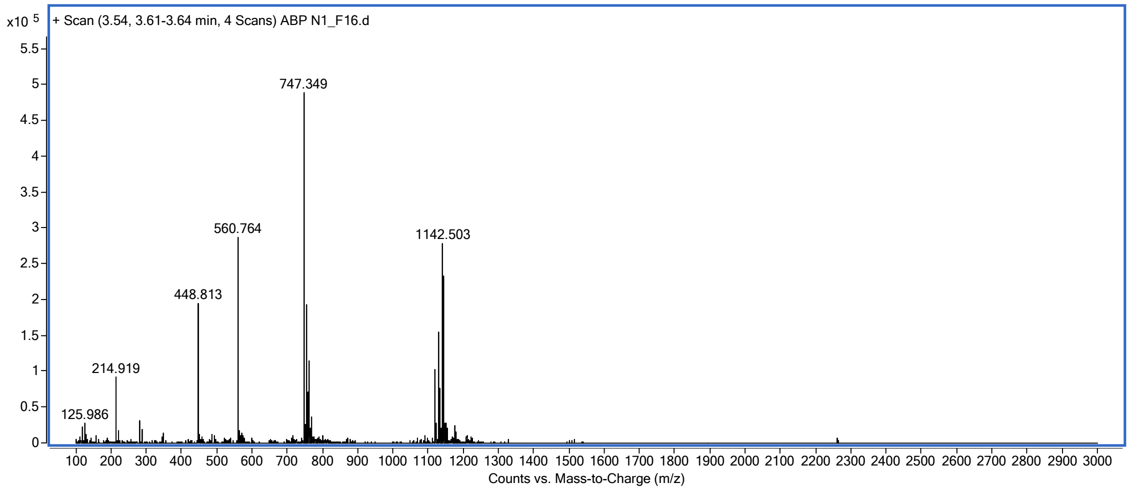

2.1.2 Biotin-ABP N4

Observed: 2131.88 Da; Calculated: 2131.93 Da

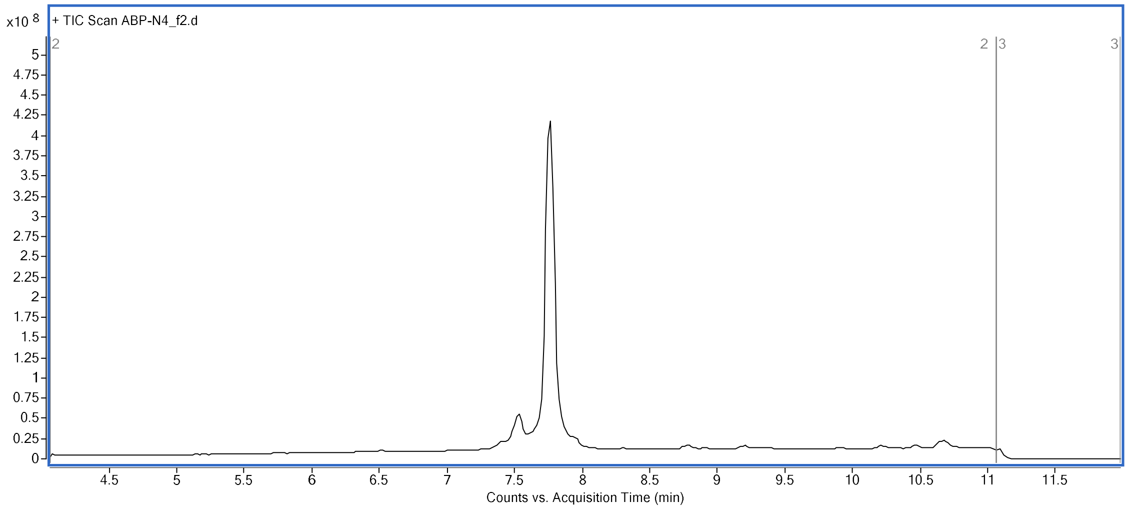

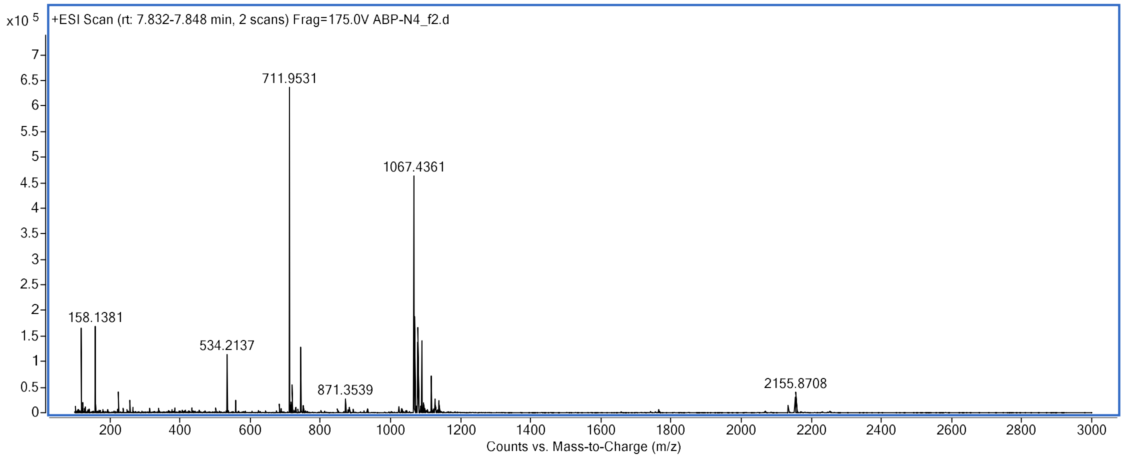

2.1.3 Biotin-ABP N5

Observed: 2196.86 Da; Calculated: 2196.90 Da

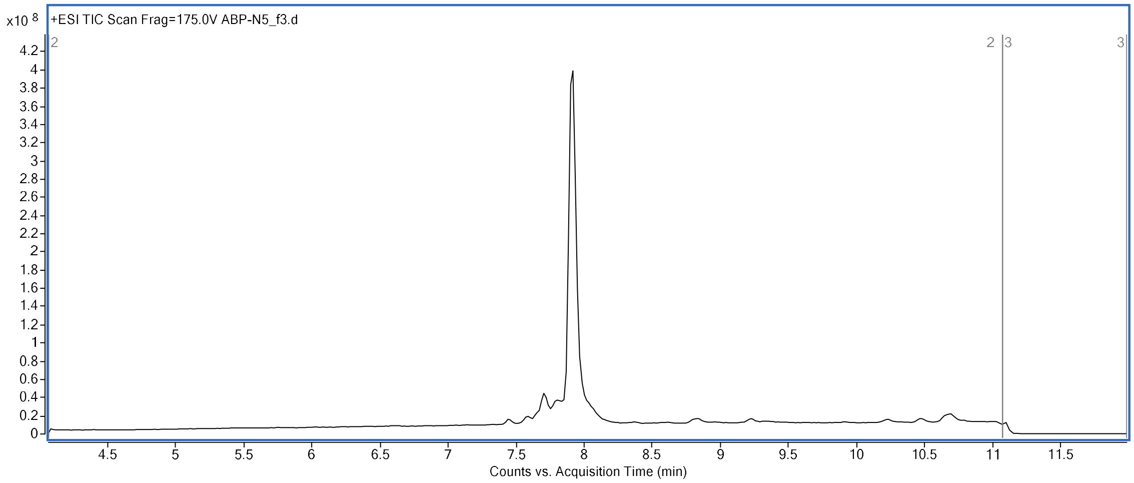

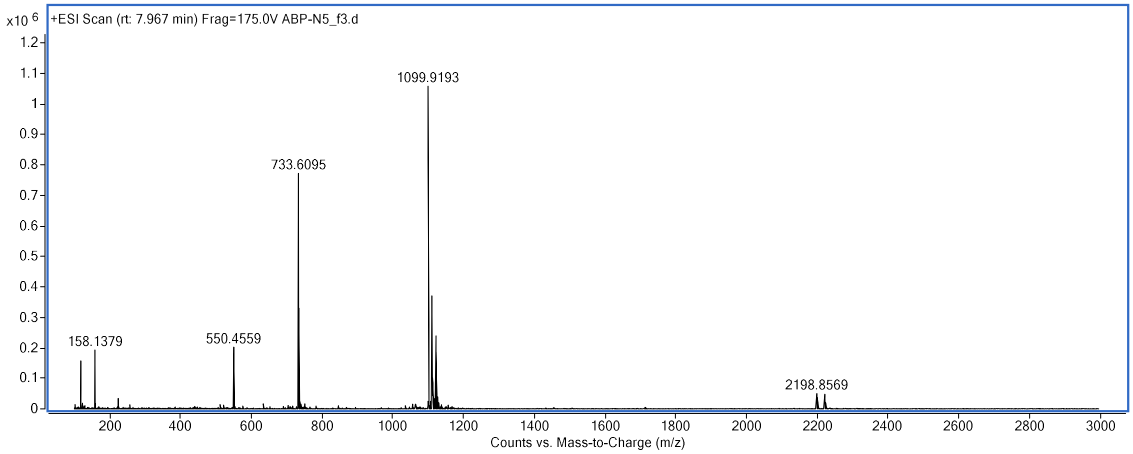

2.1.4 Biotin-ABP N6

Observed: 2267.03 Da; Calculated: 2267.07 Da

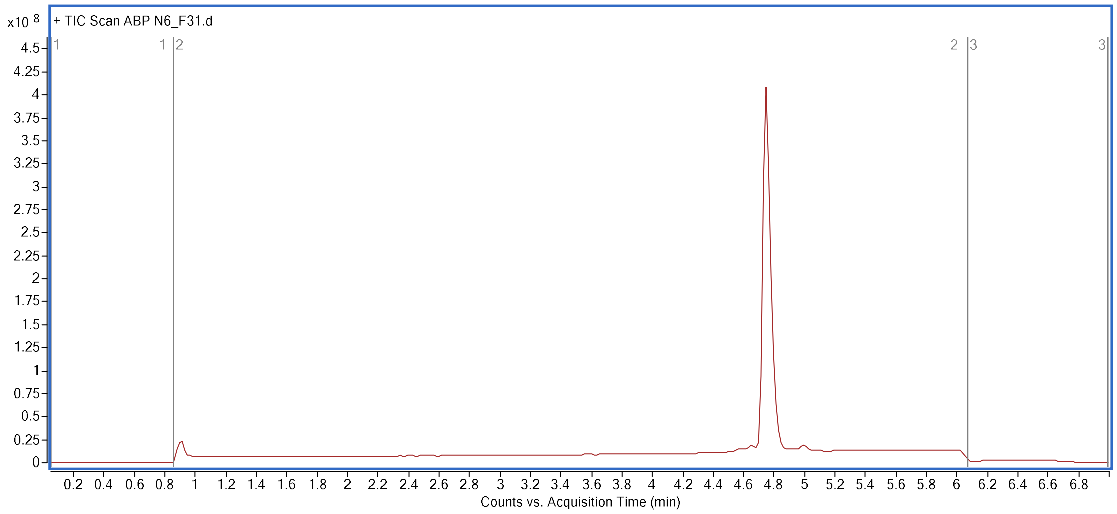

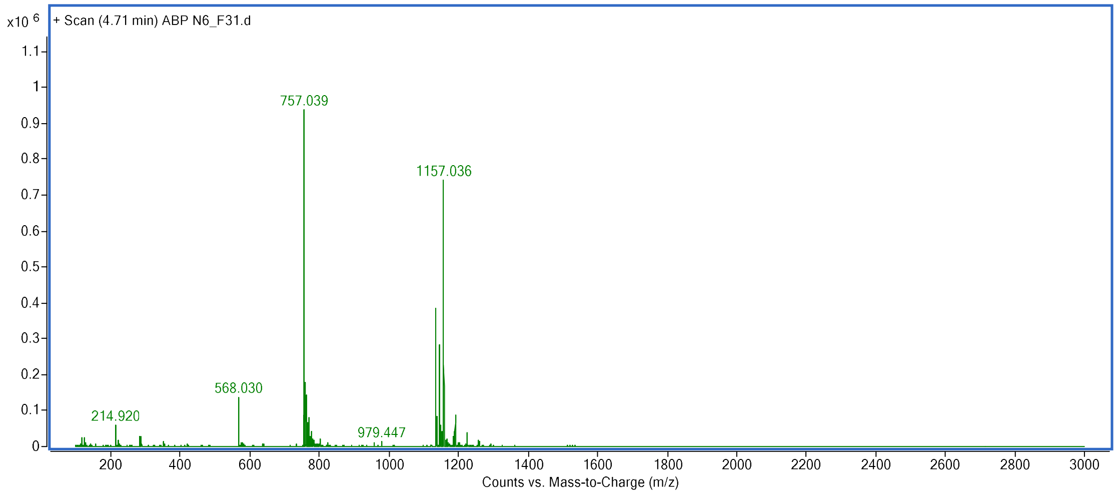

2.1.5 Biotin-ABP N8

Observed: 2267.97 Da; Calculated: 2268.02 Da

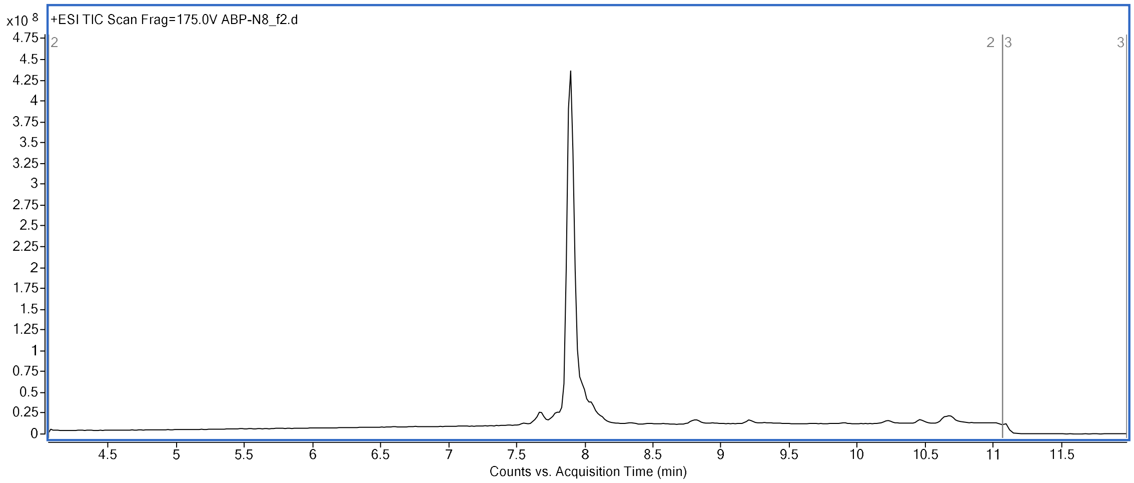

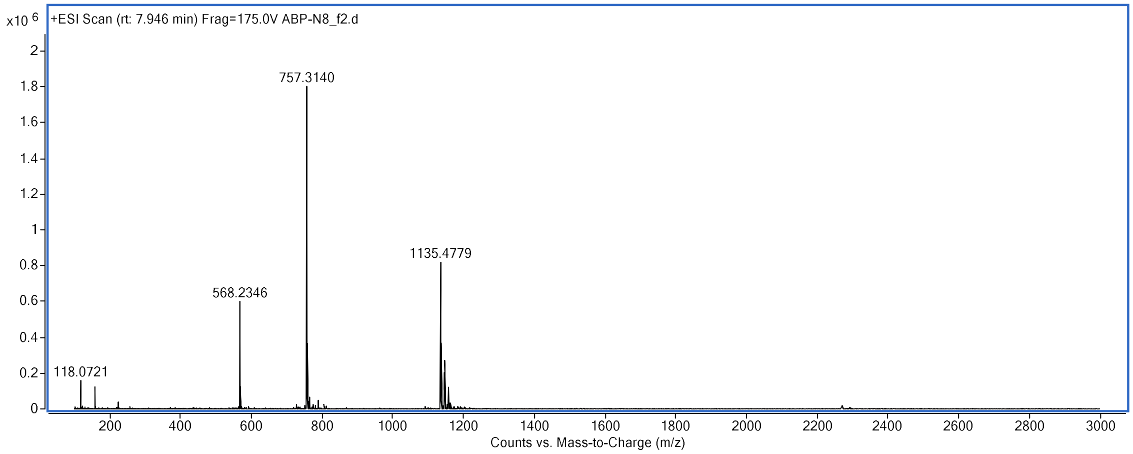

2.1.6 ABP N4

Observed: 1633.68 Da; Calculated: 1633.70 Da

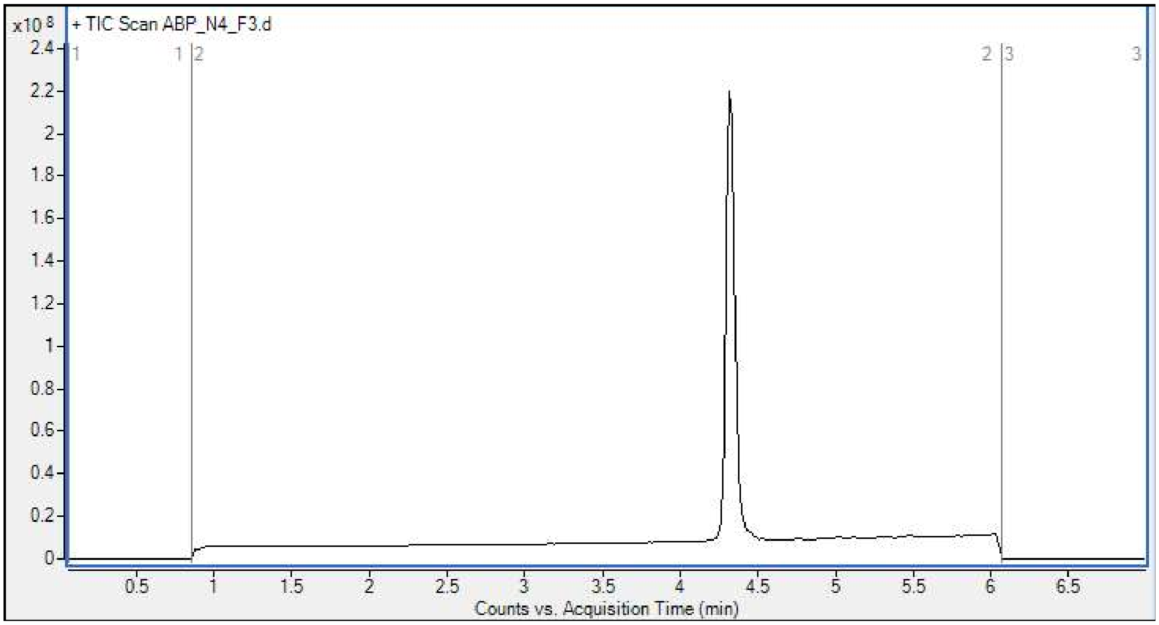

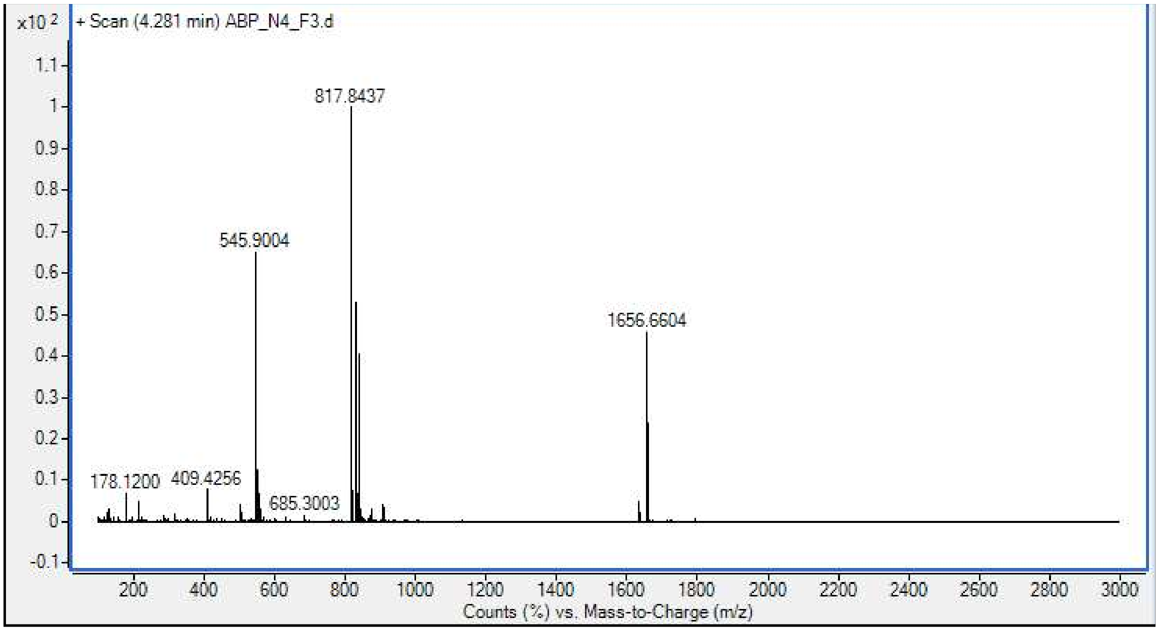

2.1.7 ABP N6

Observed: 1768.97 Da; Calculated: 1768.80 Da

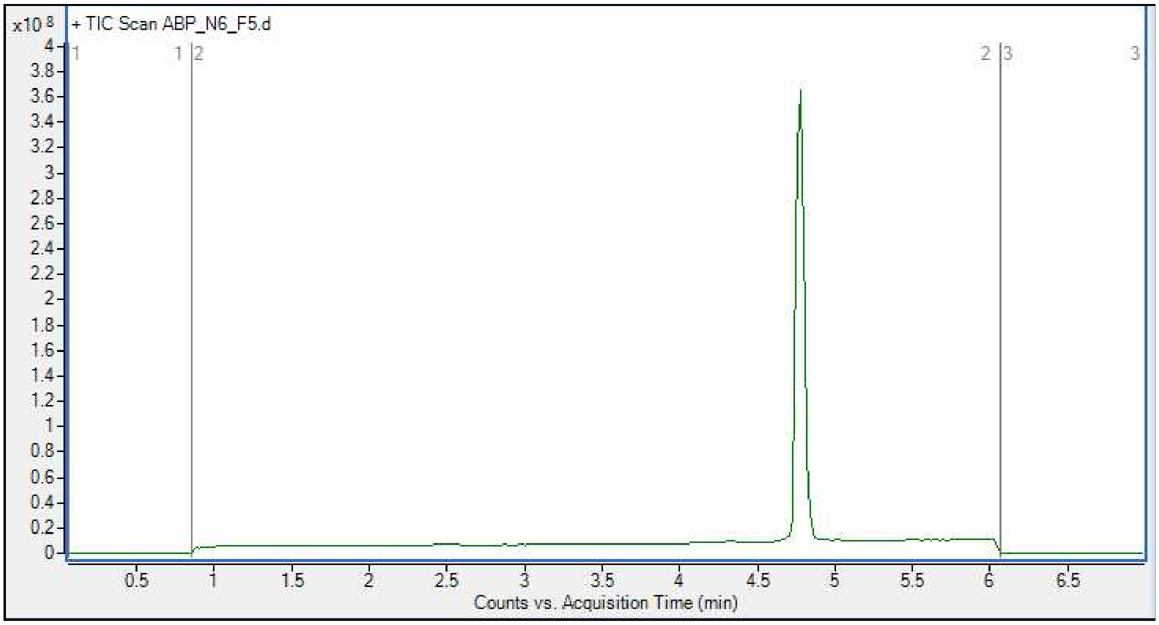

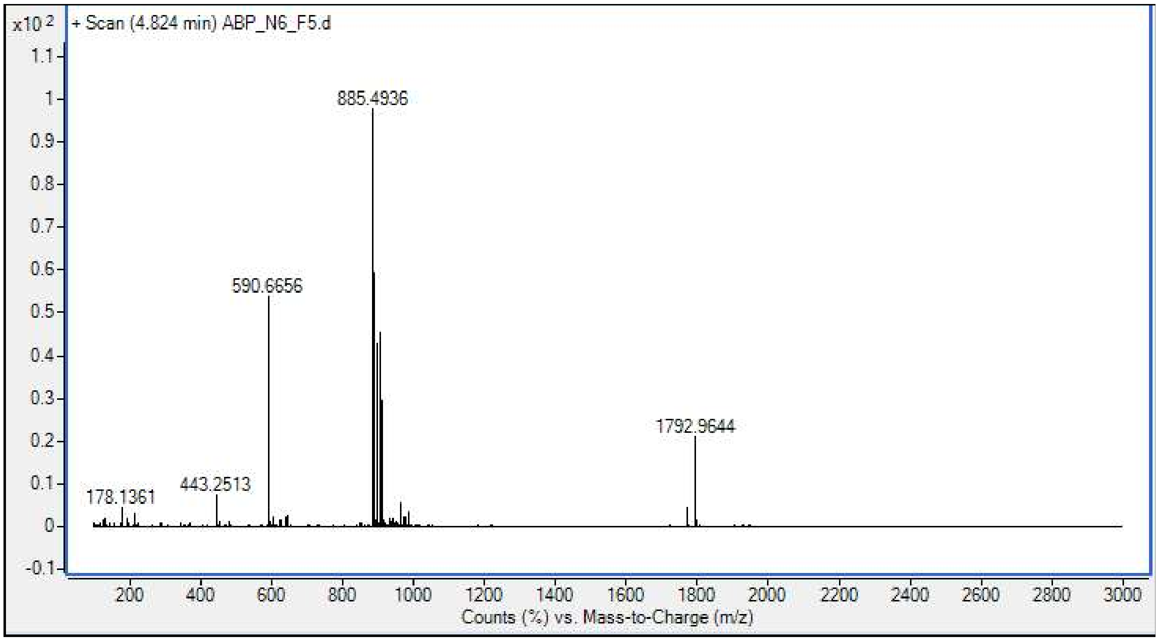

2.2 LC-MS traces of purified canonical peptides

2.2.1 Biotin-ABP C1

Observed: 2041.06 Da; Calculated: 2041.11 Da

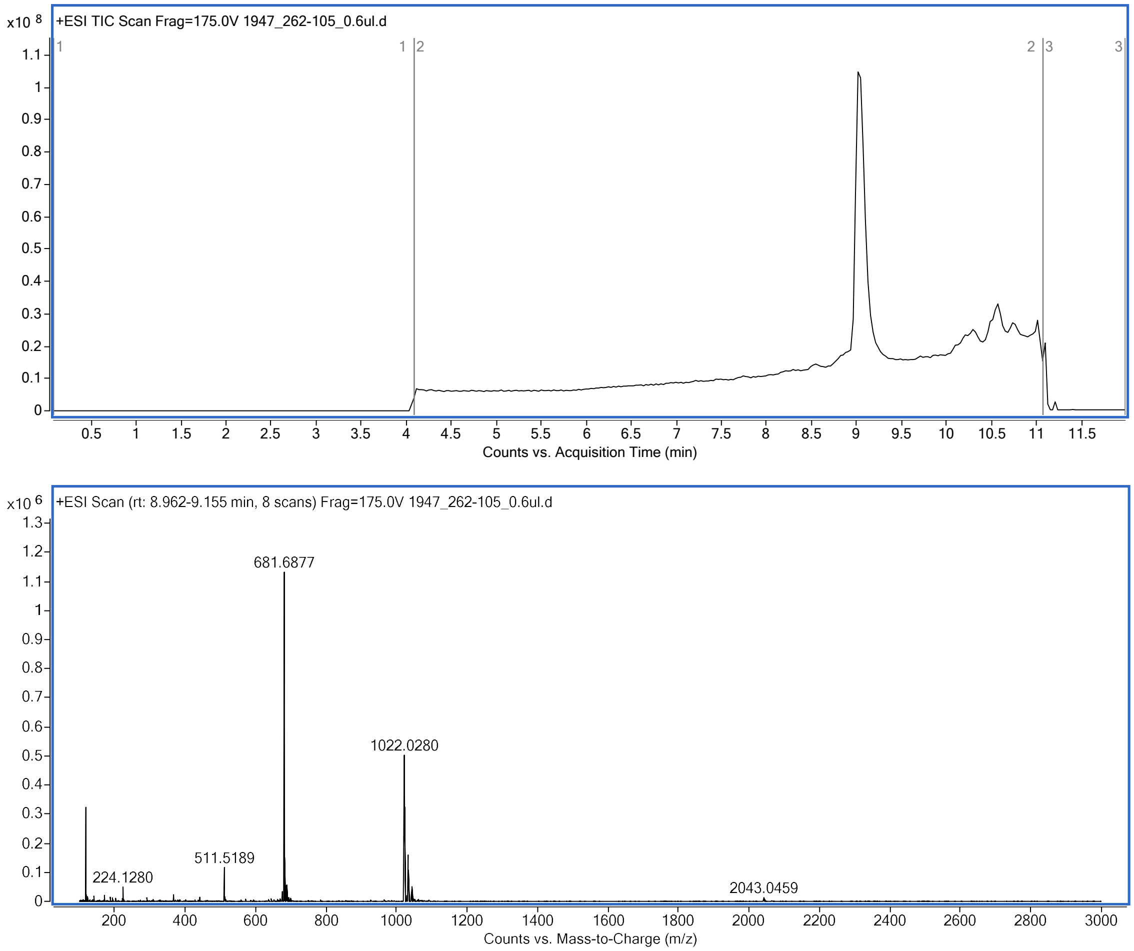

2.2.2 Biotin-ABP C2

Observed: 2235.15 Da; Calculated: 2235.19 Da

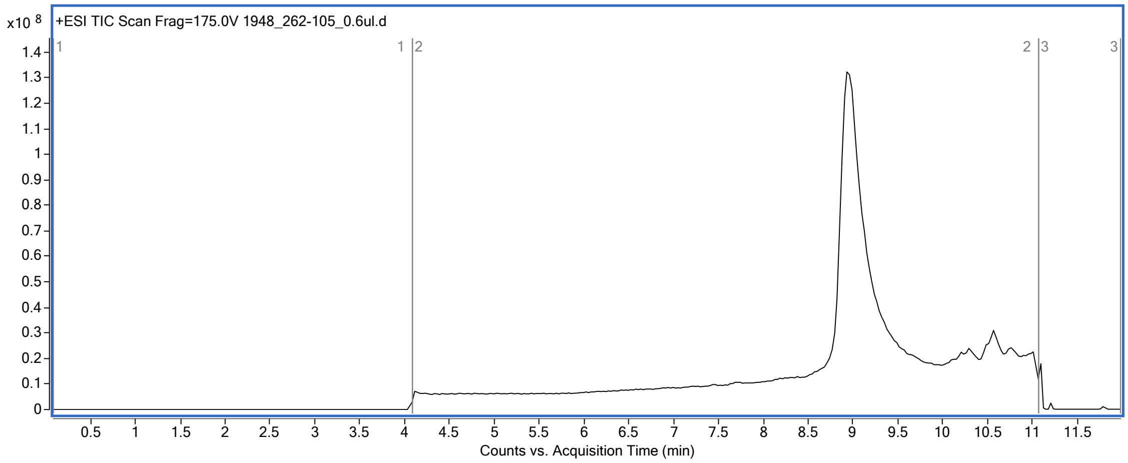

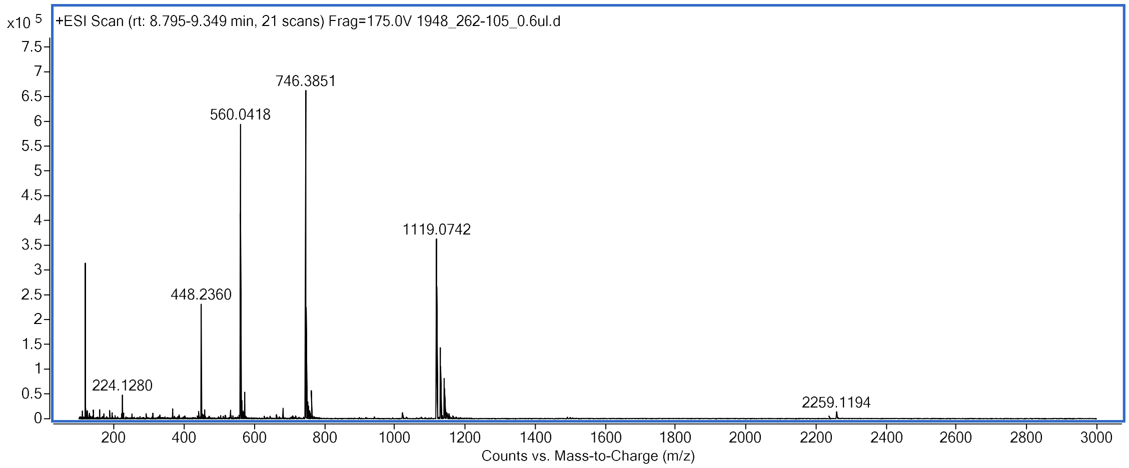

2.2.3 Biotin-ABP C3

Observed: 2214.14 Da; Calculated: 2214.15 Da

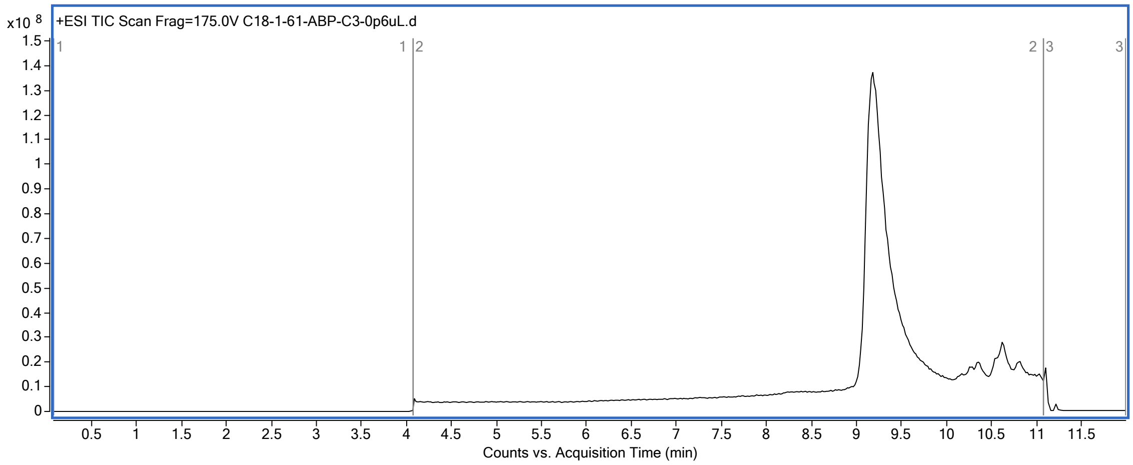

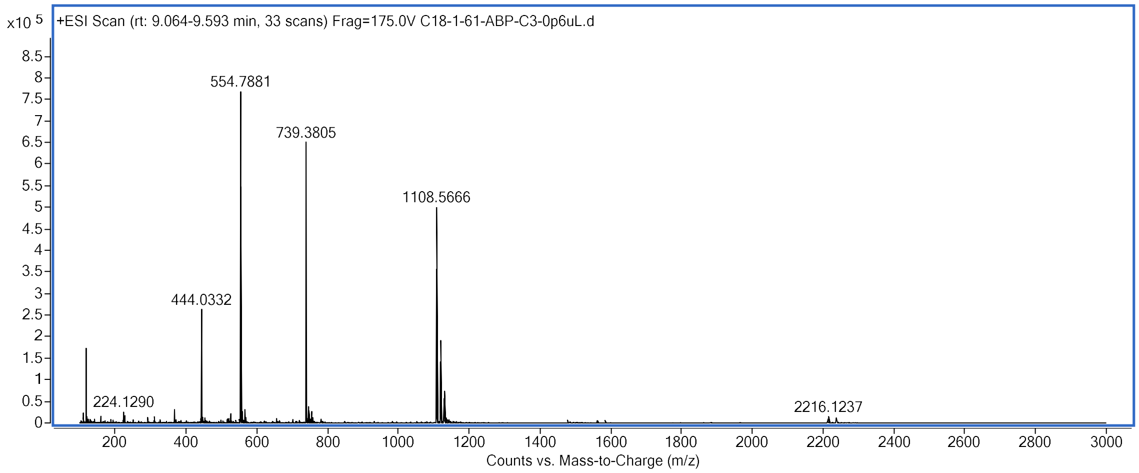

2.2.4 Biotin-ABP C4

Observed: 2187.15 Da; Calculated: 2187.17 Da

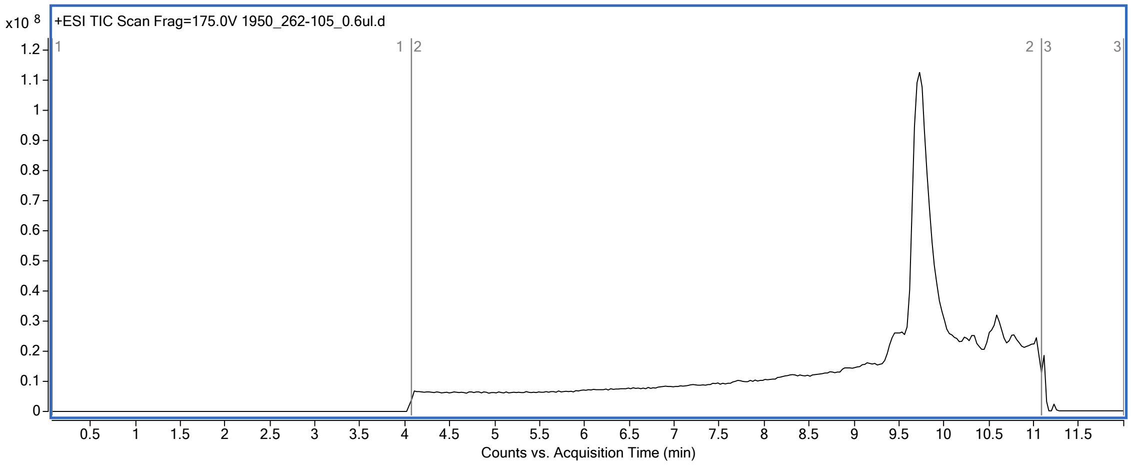

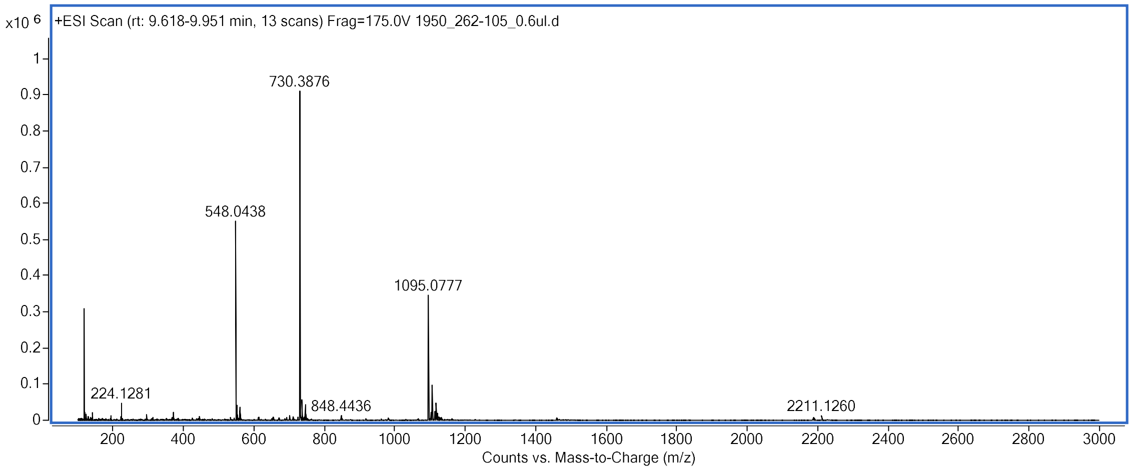

2.2.5 Biotin-ABP C5

Observed: 2082.16 Da; Calculated: 2082.18 Da

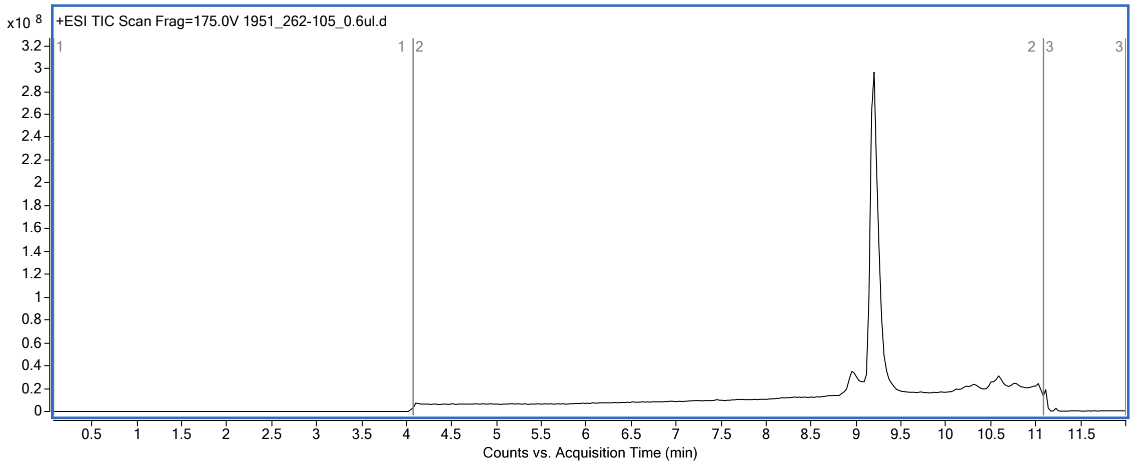

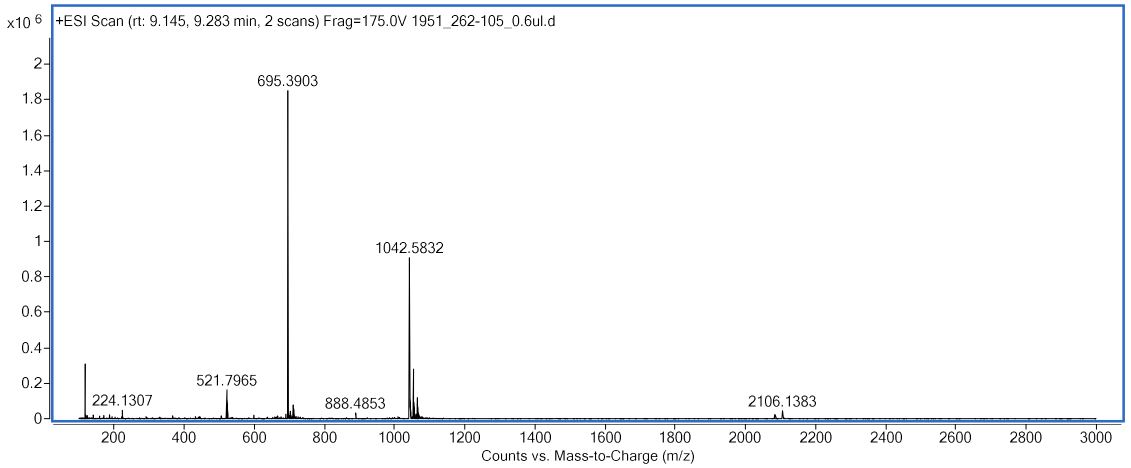

2.2.6 Biotin-ABP C6

Observed: 2102.14 Da; Calculated: 2102.16 Da

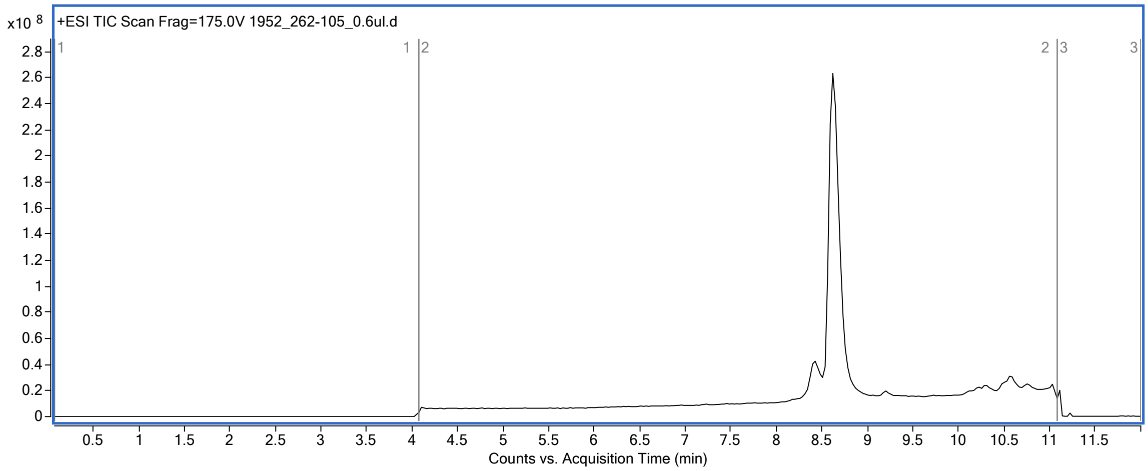

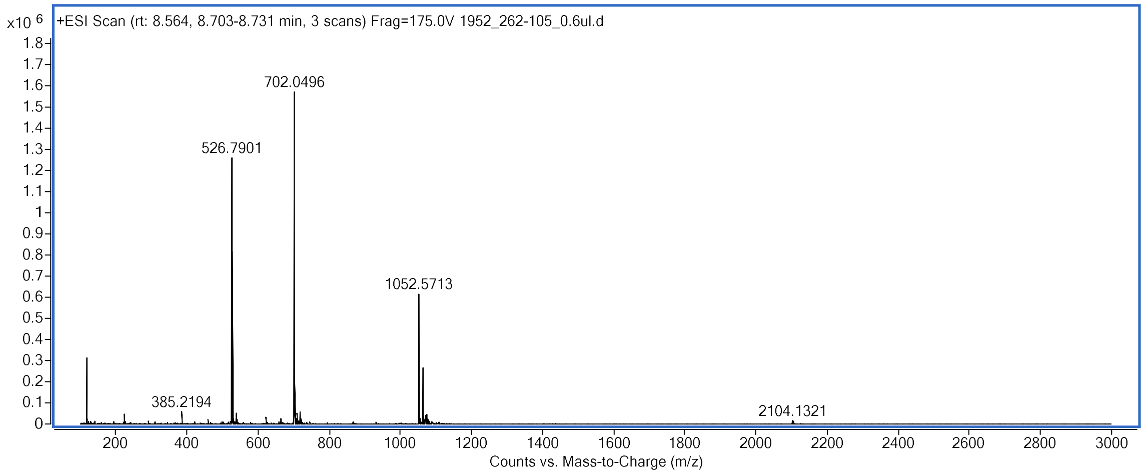

2.2.7 Biotin-ABP C7

Observed: 2063.14 Da; Calculated: 2063.16 Da

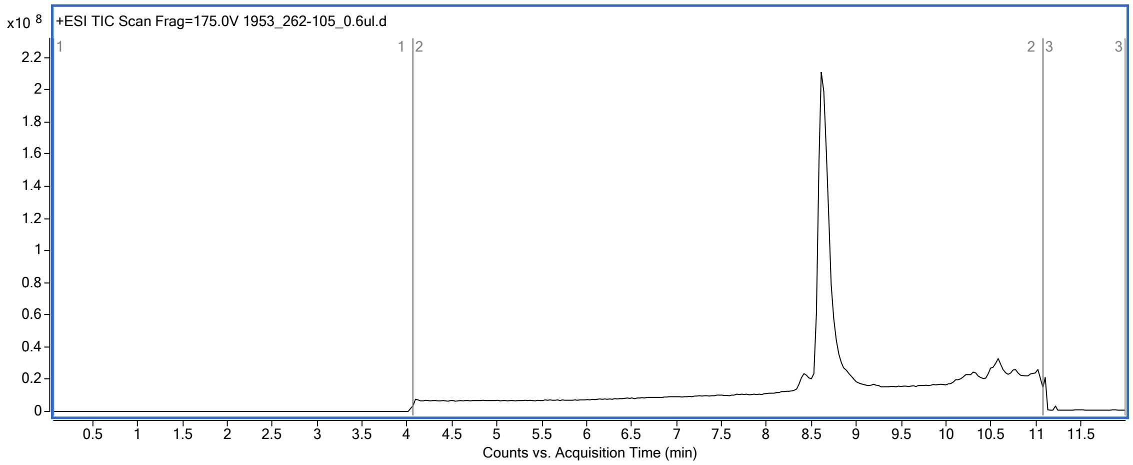

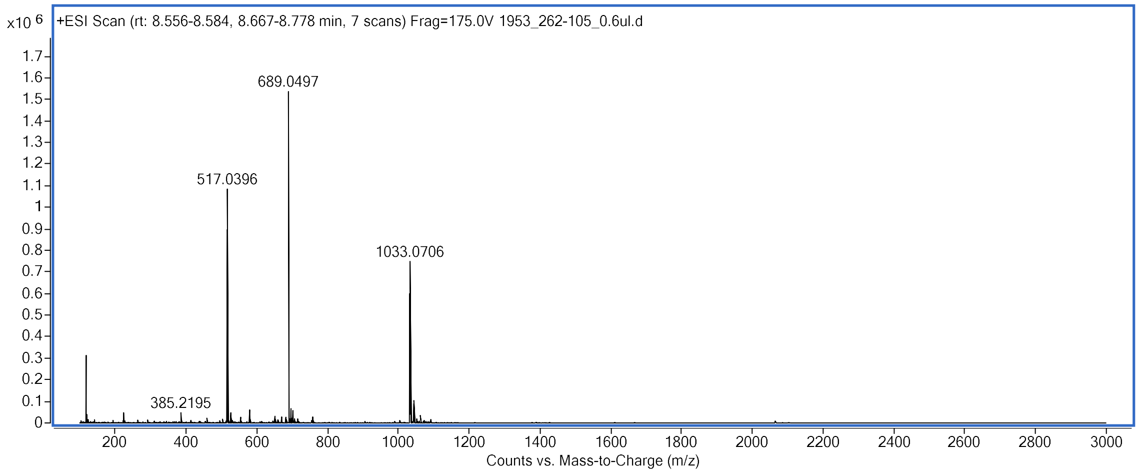

2.2.8 Biotin-ABP C8

Observed: 2230.11 Da; Calculated: 2230.13 Da

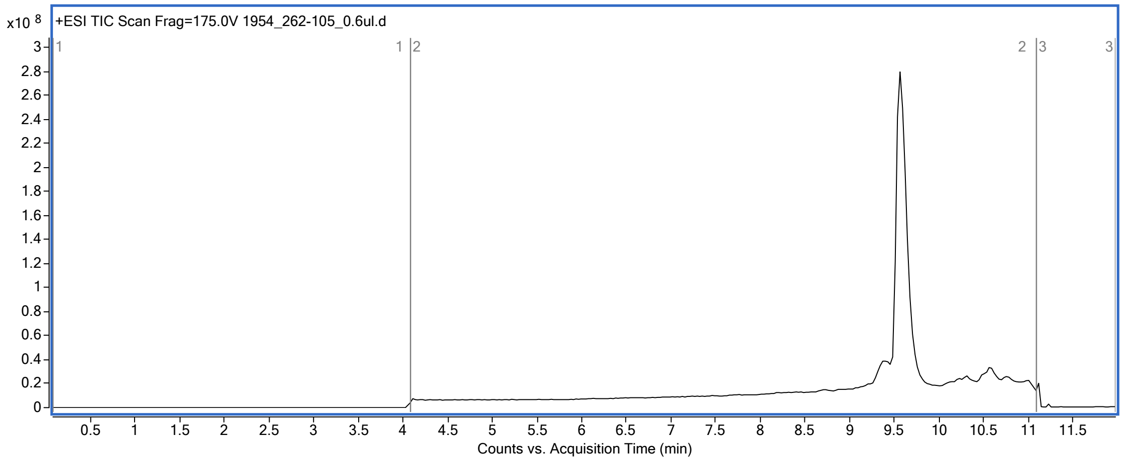

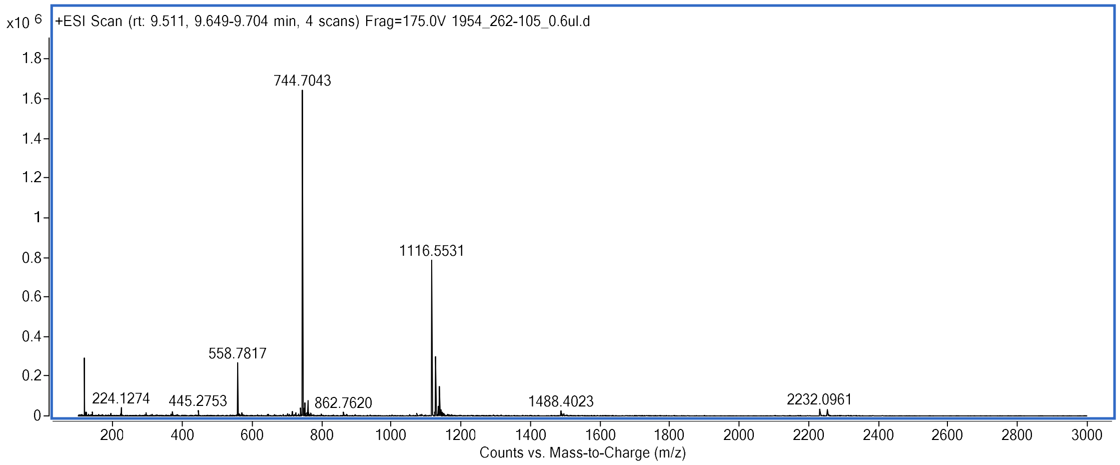

2.2.9 Biotin-ABP C9

Observed: 2155.09 Da; Calculated: 2155.12 Da

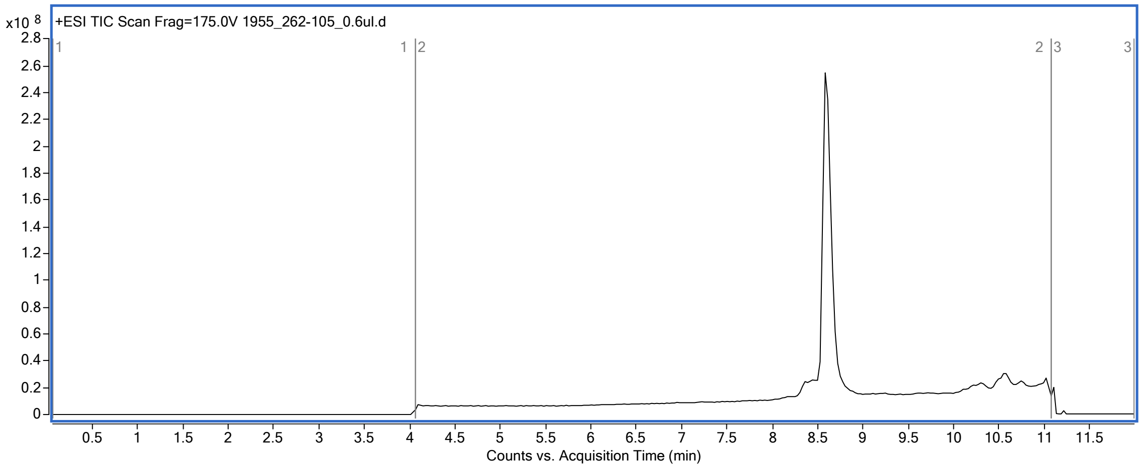

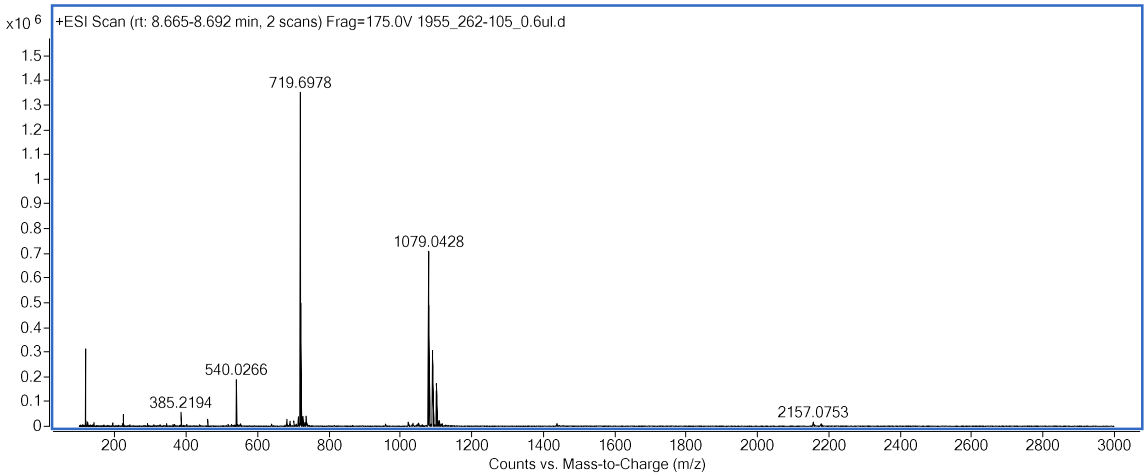

2.2.10 ABP C1

Observed: 1543.80 Calculated [M+H]: 1543.82

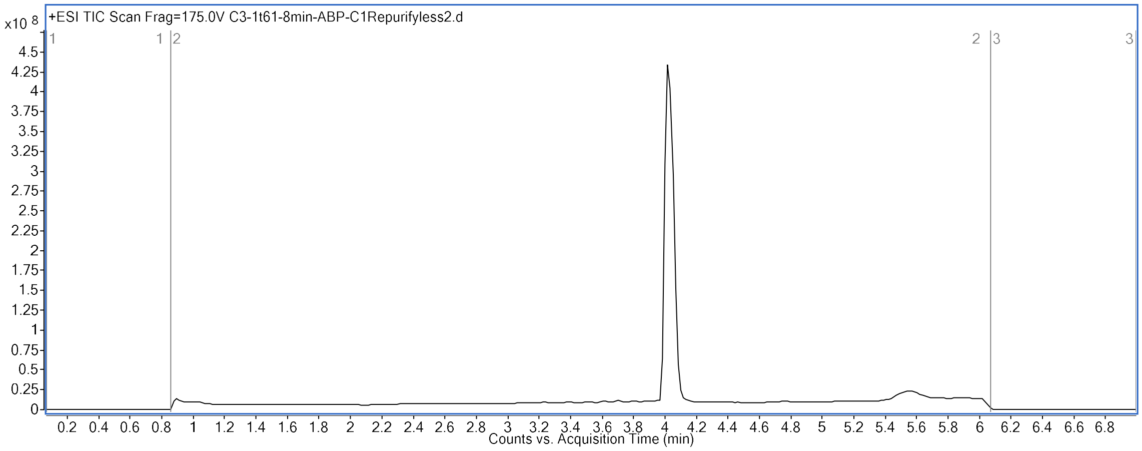

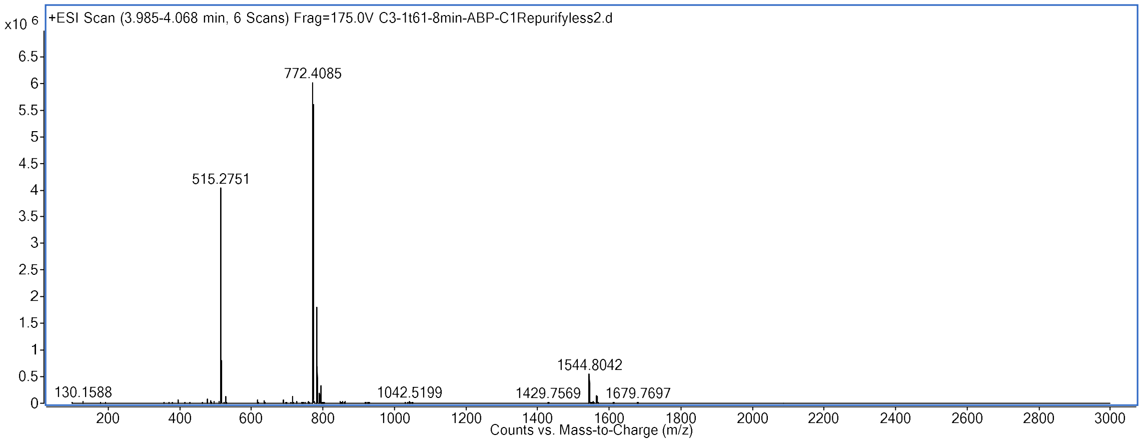

2.2.11 ABP C2

Observed: 1737.89 Calculated [M+H]: 1737.91

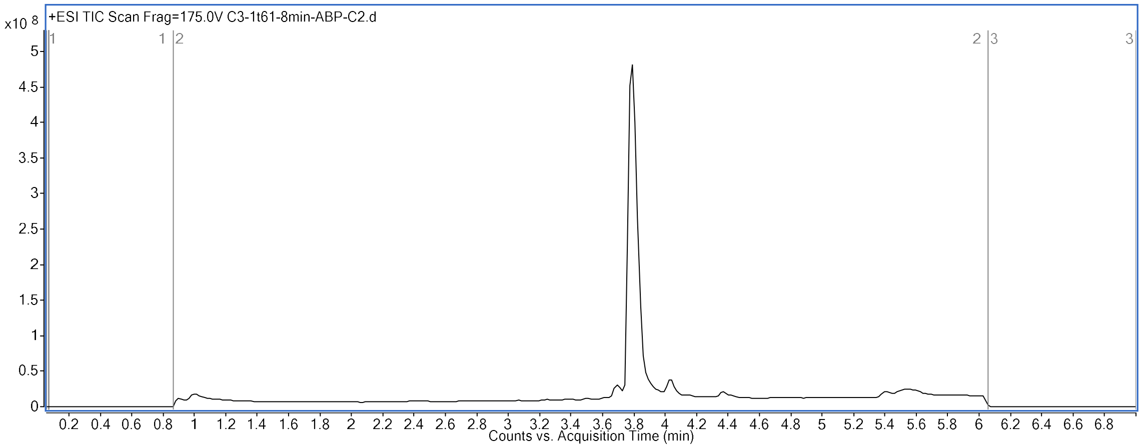

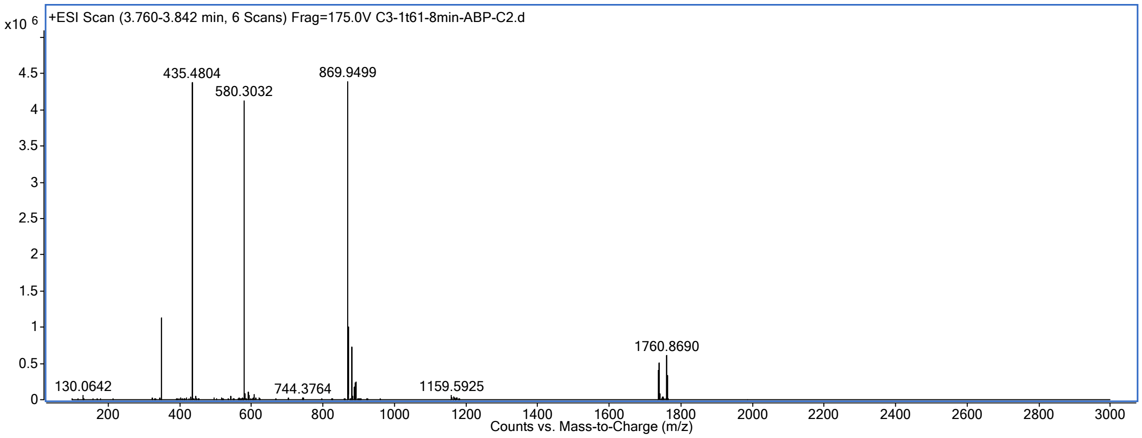

2.2.12 ABP C3

Observed: 1716.87 Calculated [M+H]: 1716.89

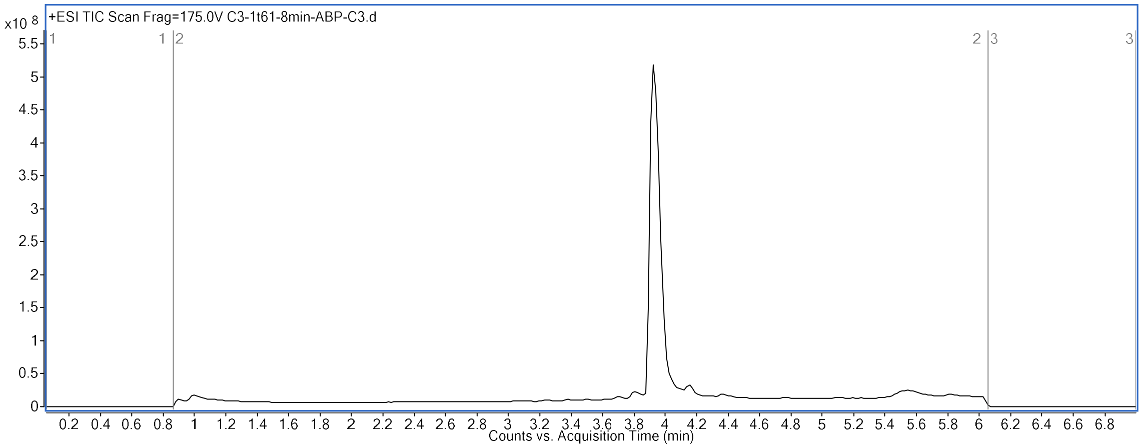

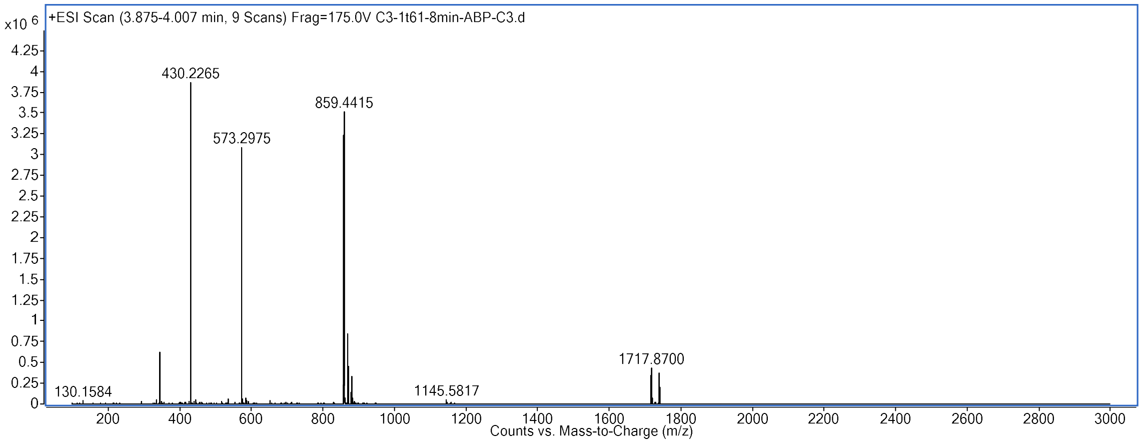

2.2.13 ABP C4

Observed: 1689.92 Calculated [M+H]: 1689.92

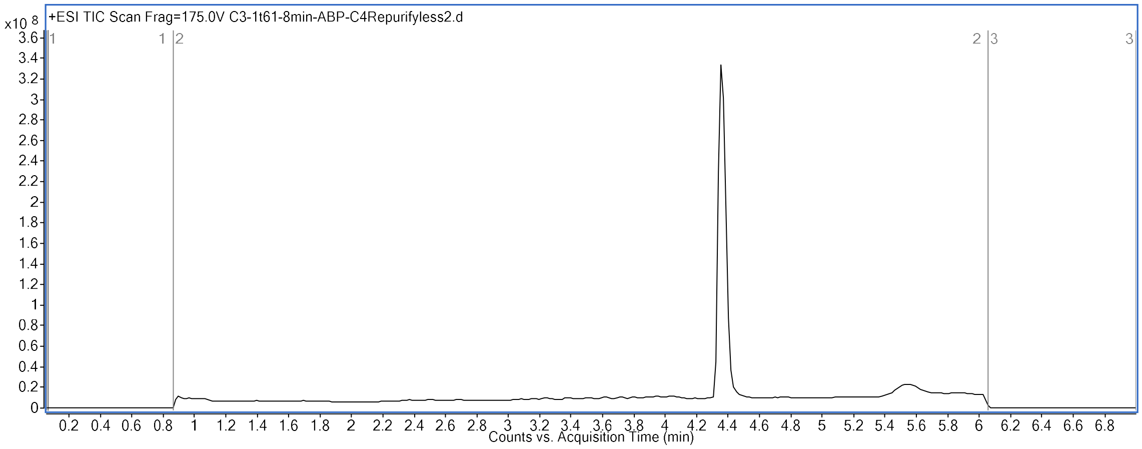

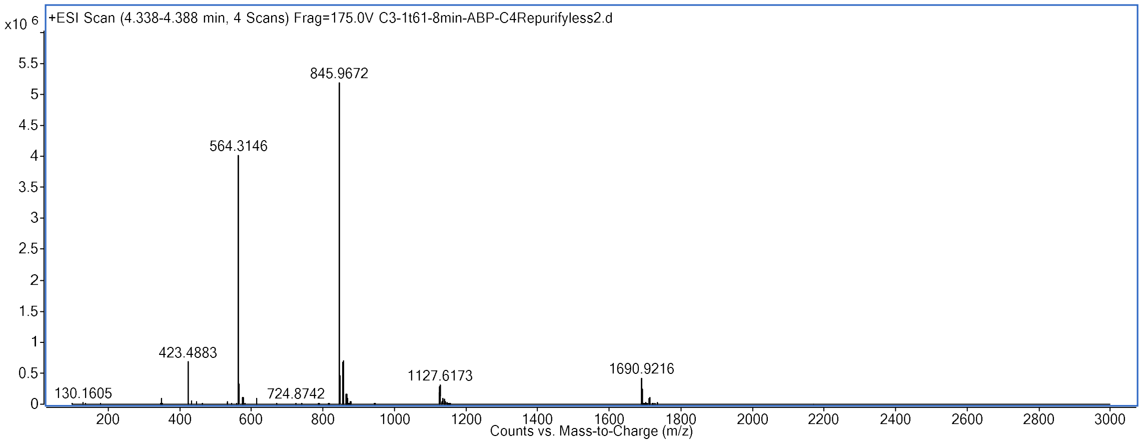

2.2.14 ABP C7

Observed: 1565.90 Calculated [M+H]: 1565.91

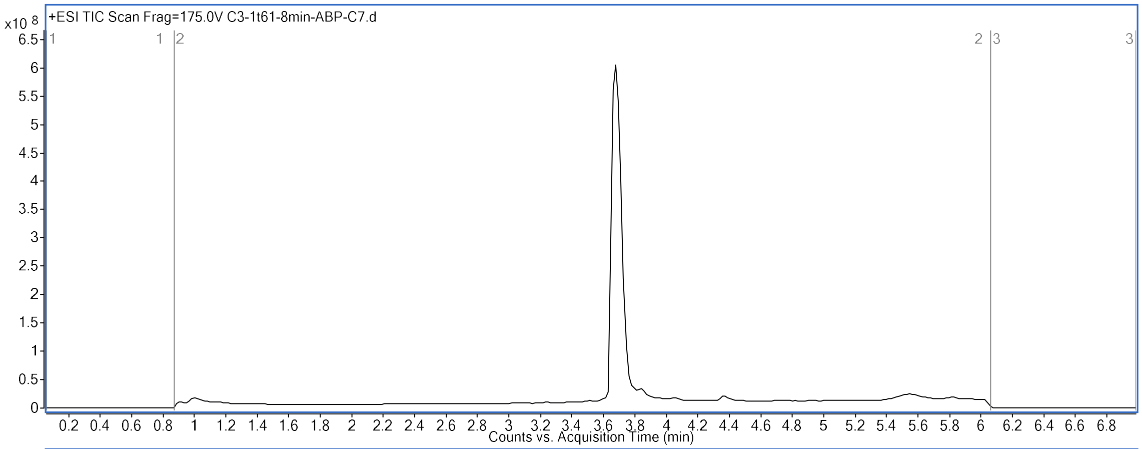

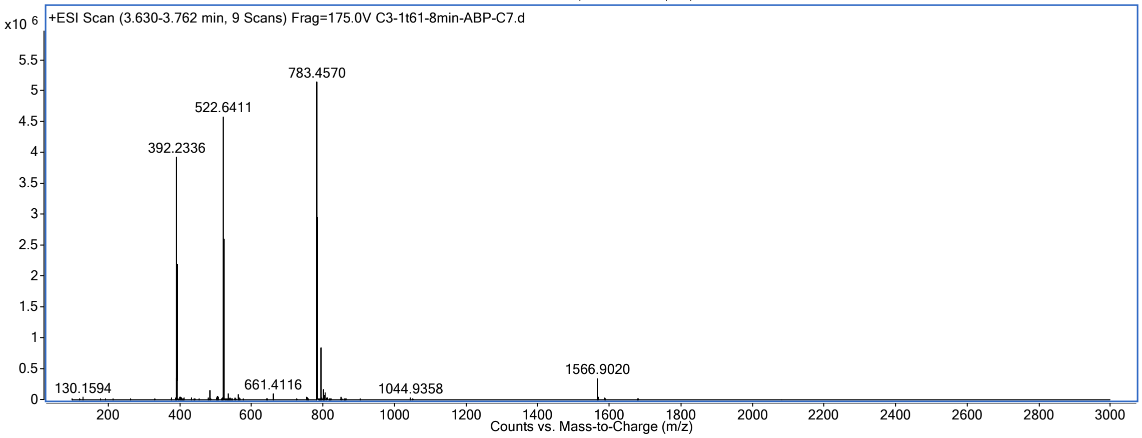

2.2.15 ABP C8

Observed: 1732.87 Calculated [M+H]: 1732.88

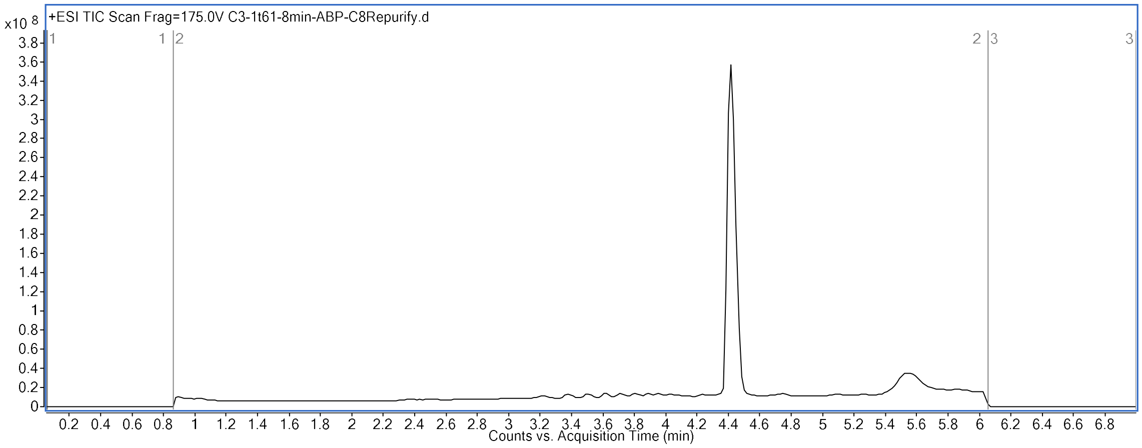

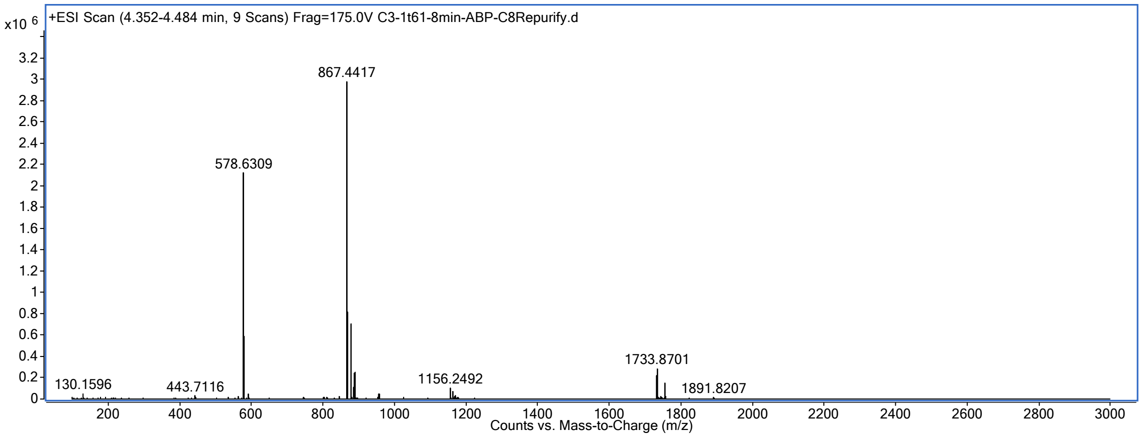

2.2.16 AngII

Observed: 1044.57 Da; Calculated: 1044.54 Da

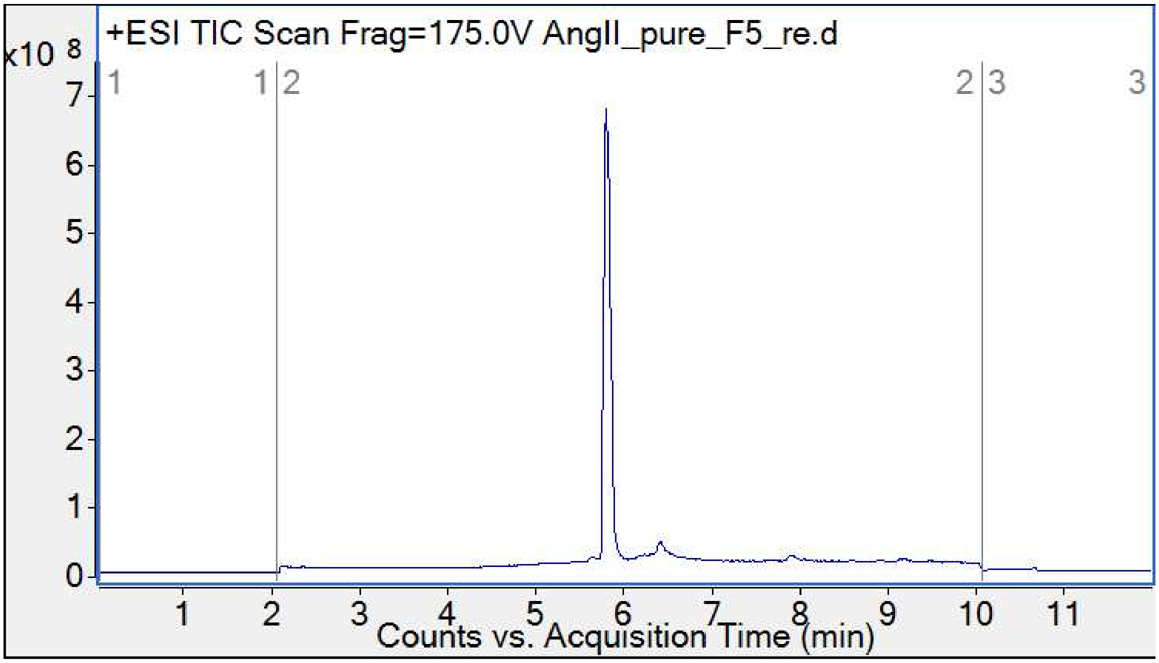

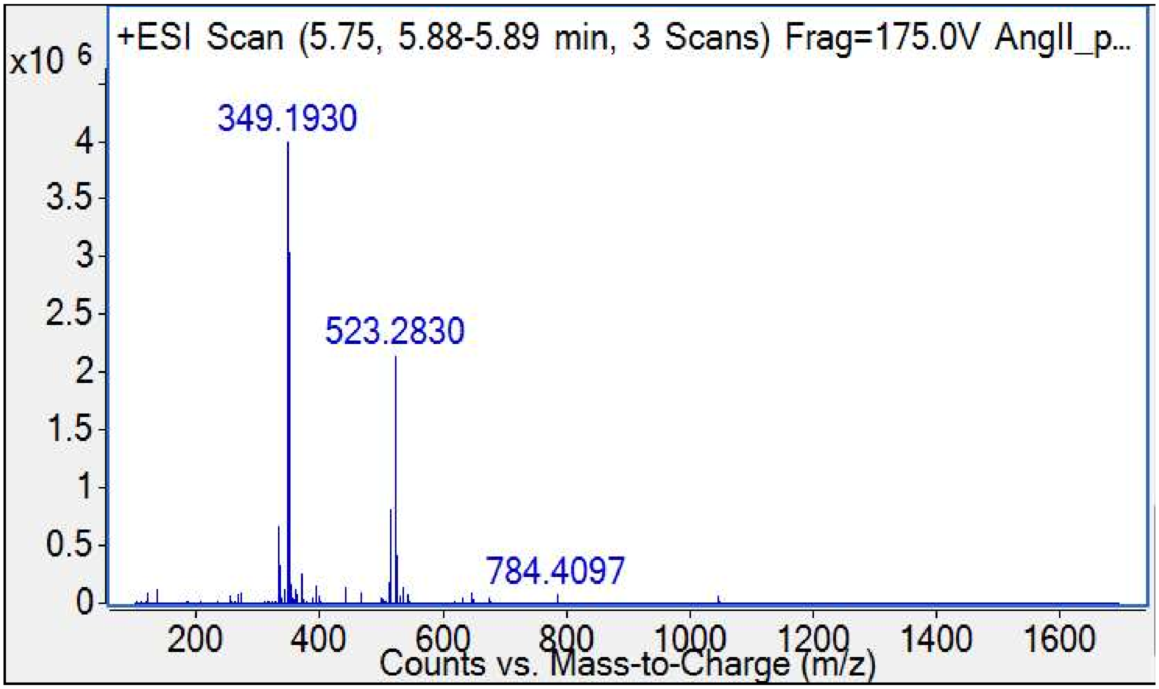

Supplementary Note 3: Analytical HPLC Data

3.1 HPLC trace of noncanonical peptides (biotinylated)

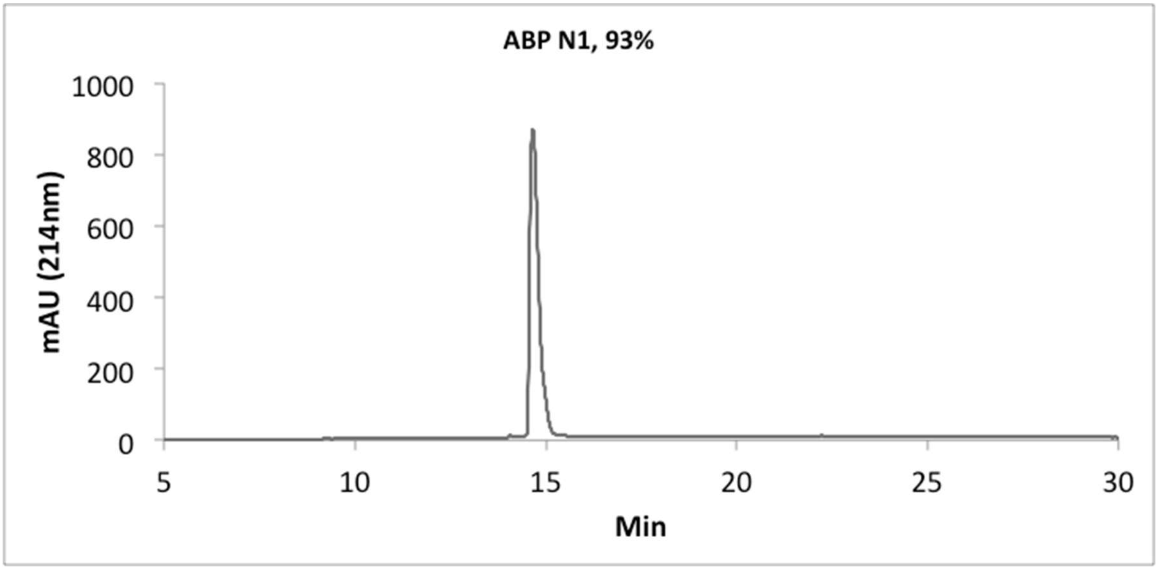

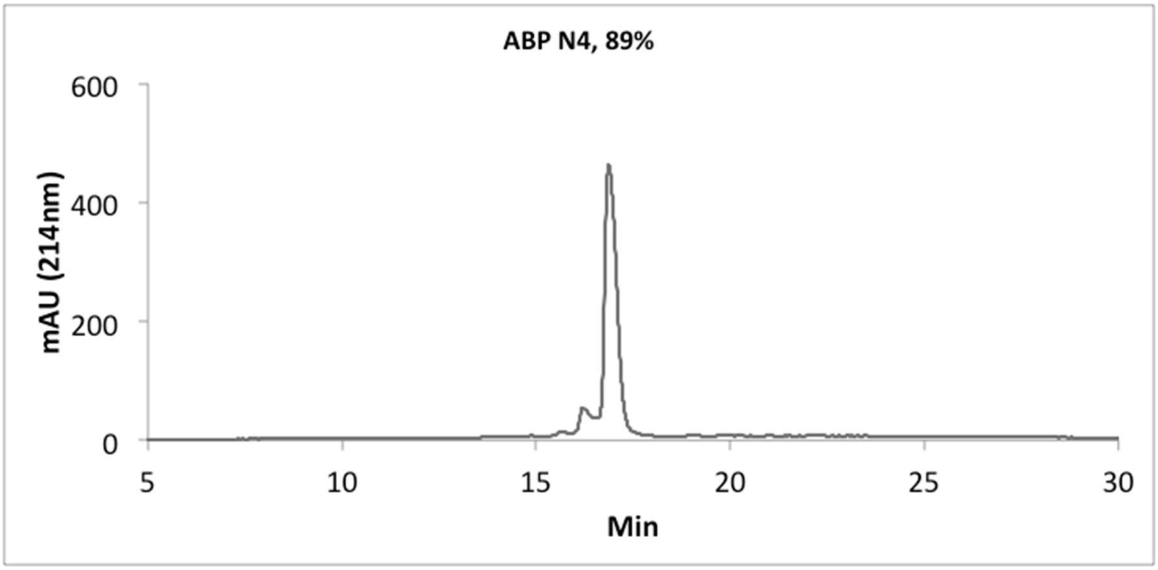

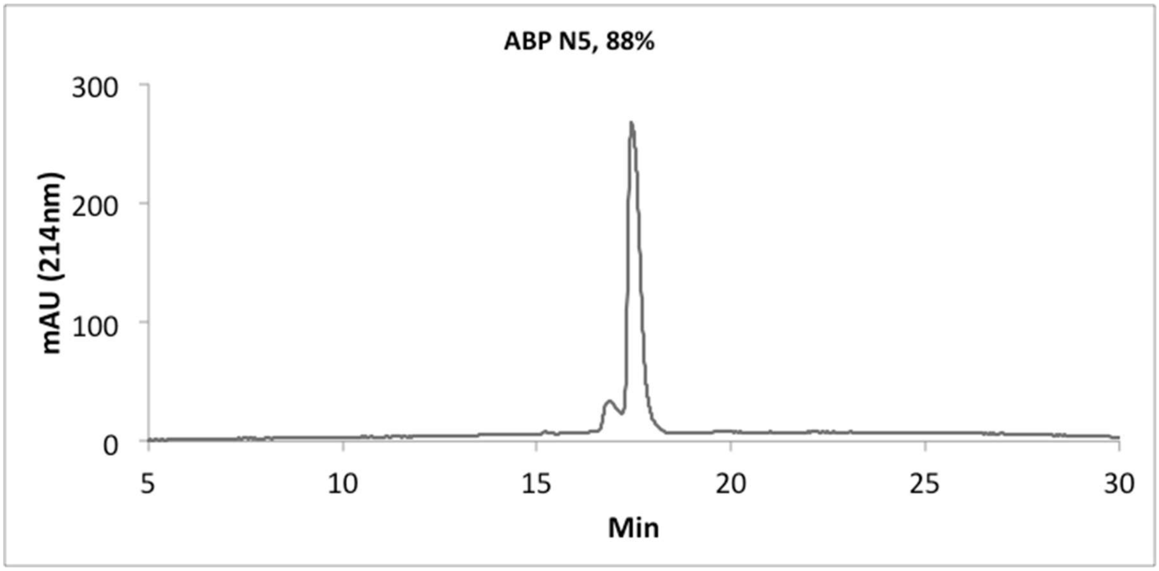
ABP N5, 88%
mAU (214nm)
300
200
100
0
5
10
15
20
25
30
Min

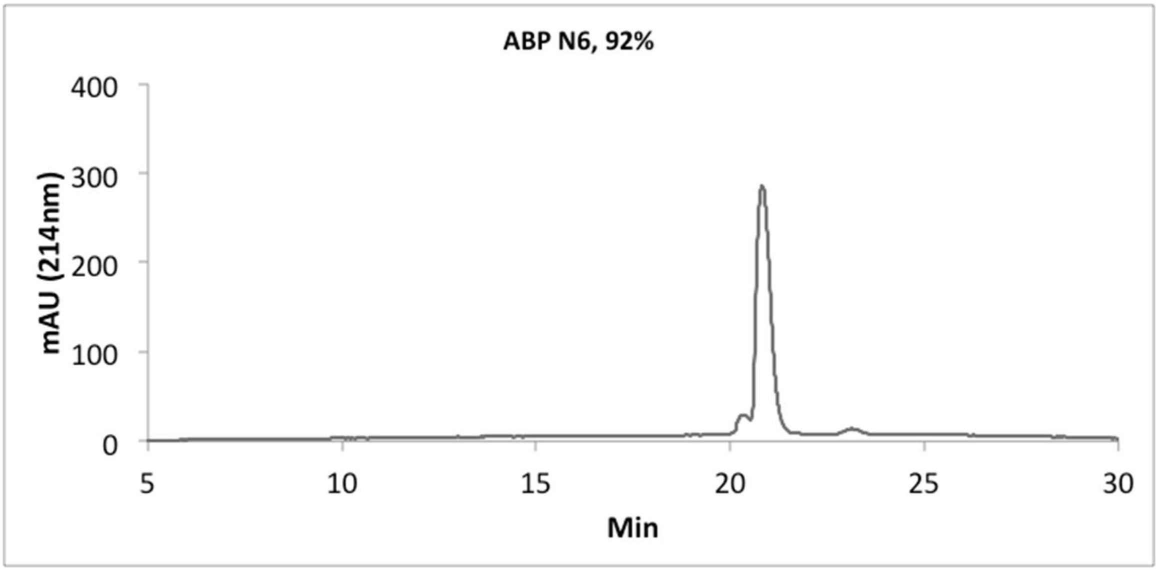
ABP N6, 92%
mAU (214nm)
400
300
200
100
0
5
10
15
20
25
30
Min

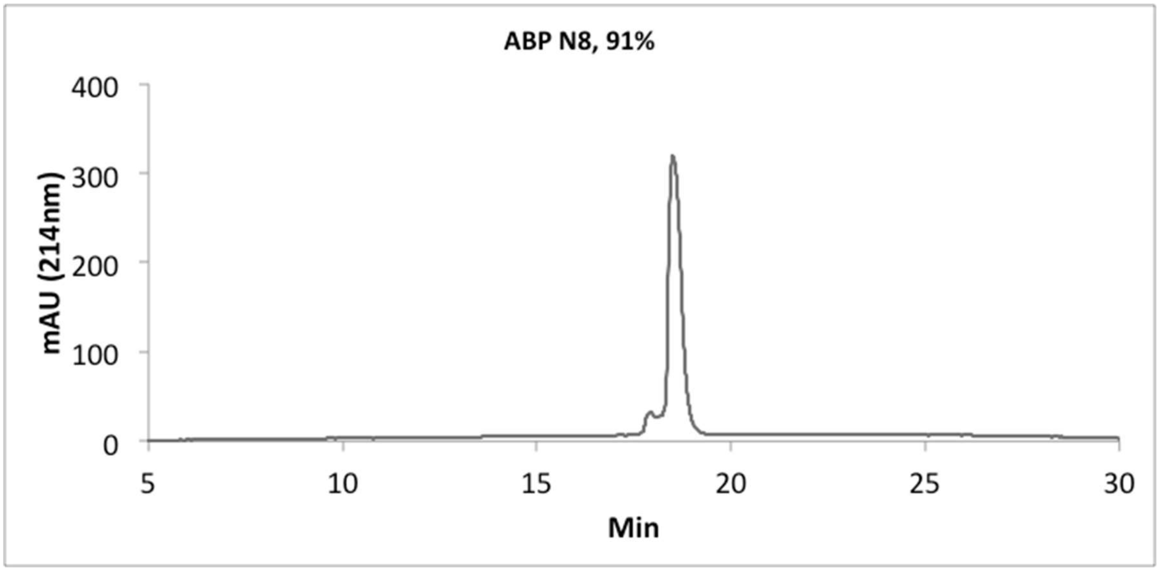

3.2 HPLC trace of noncanonical peptides (nonbiotinylated)

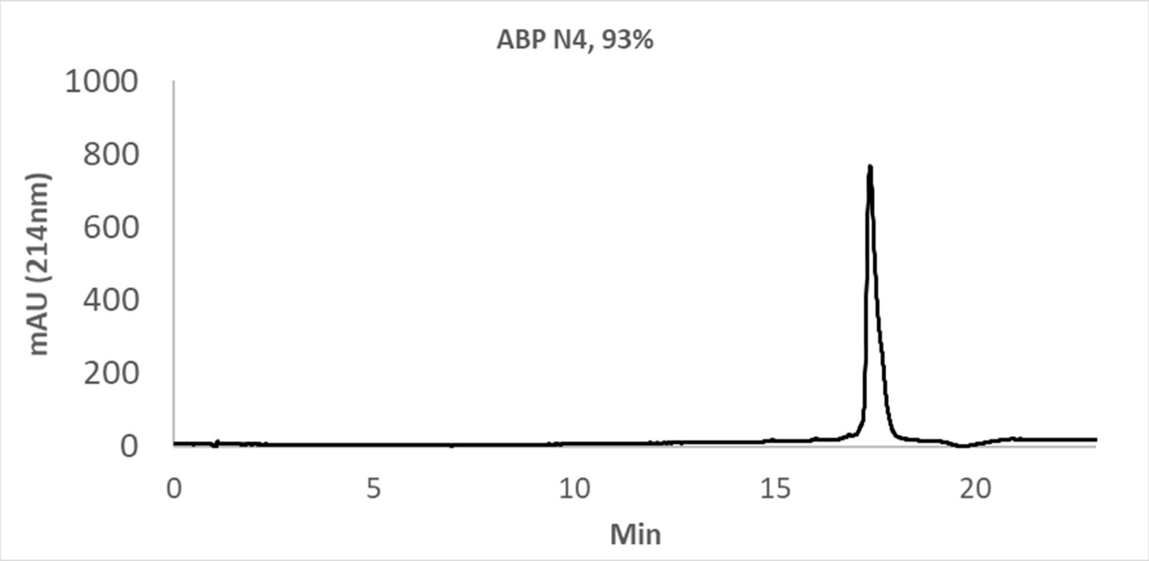

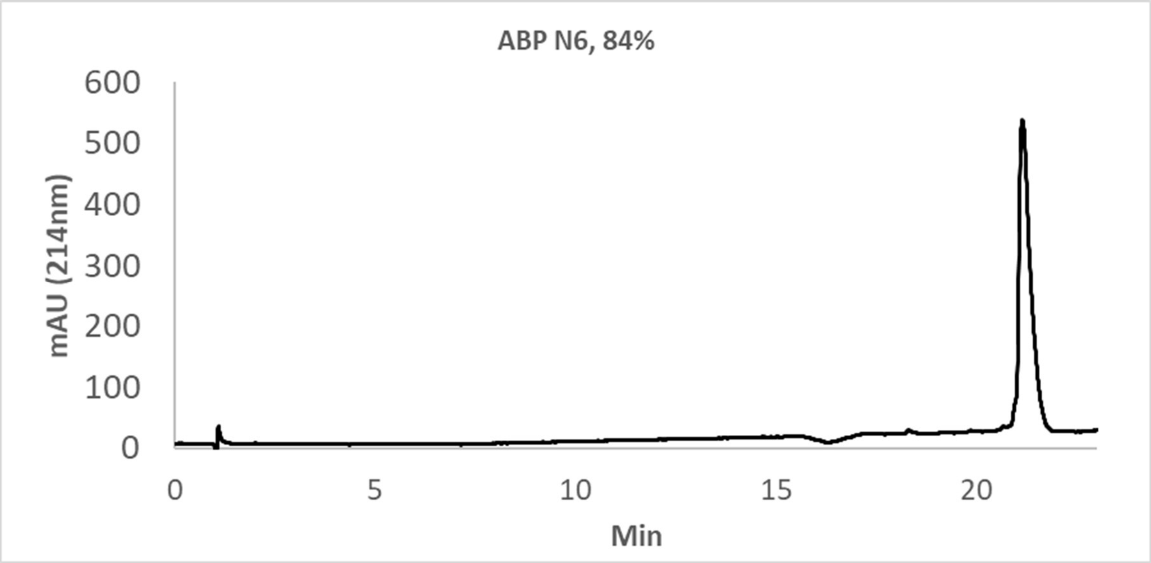

3.3 HPLC trace of canonical peptides (biotinylated)

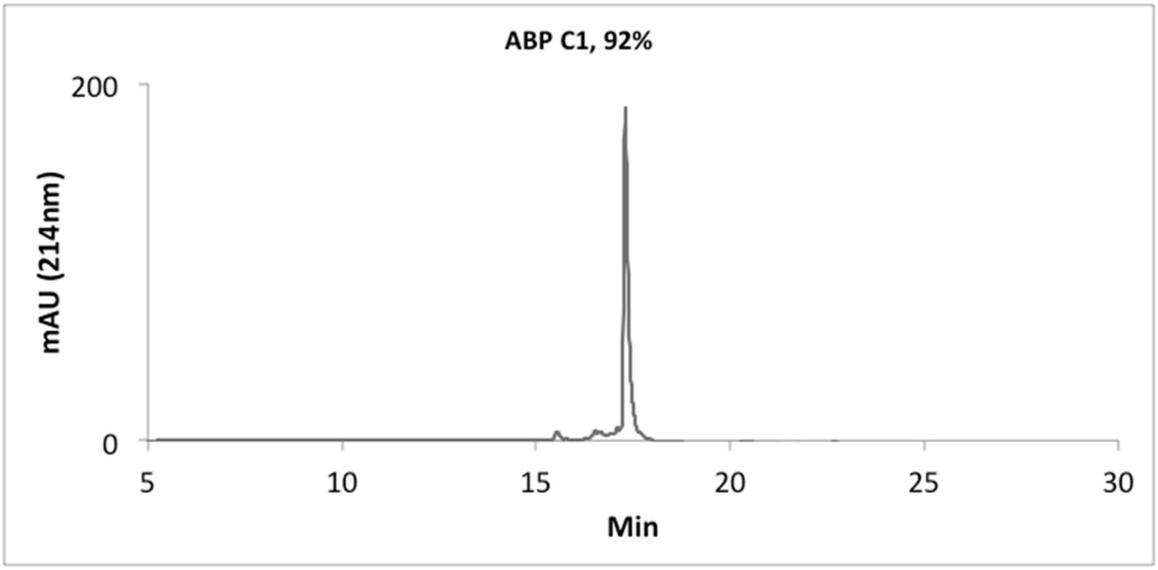

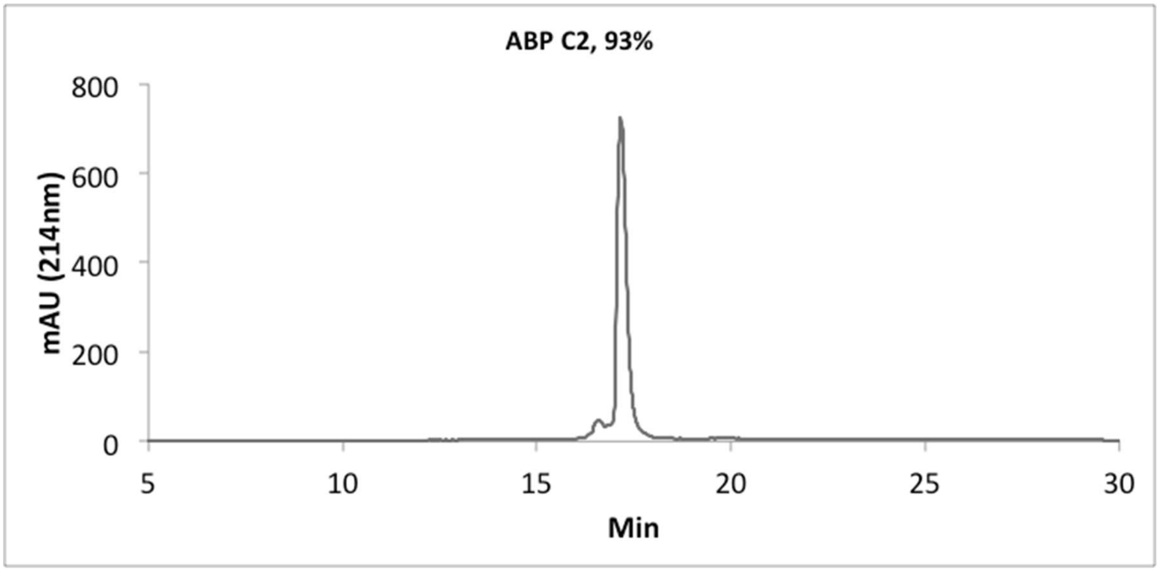
ABP C2, 93%
800
600
400
200
0
mAU (214nm)
5
10
15
20
25
30
Min

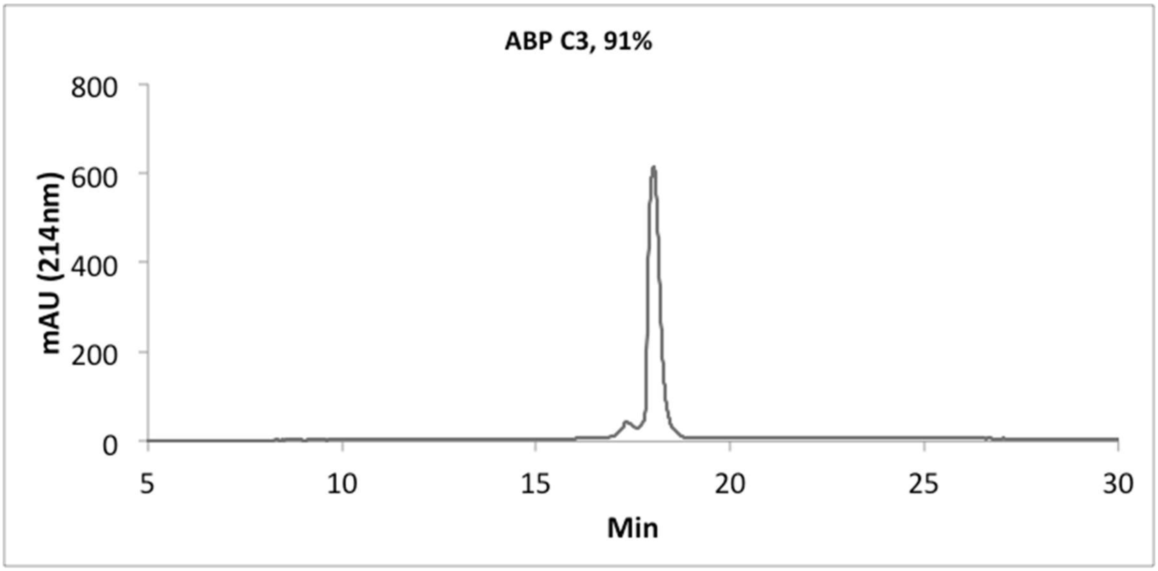
ABP C3, 91%
800
600
400
200
0
mAU (214nm)
5
10
15
20
25
30
Min

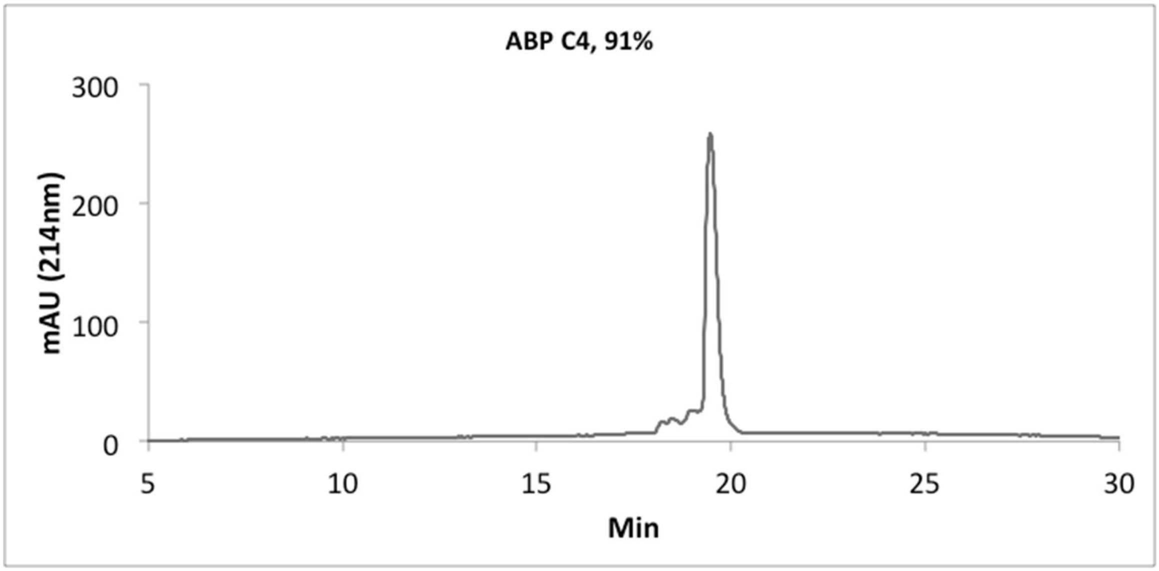
ABP C4, 91%
300
200
100
0
mAU (214nm)
5
10
15
20
25
30
Min

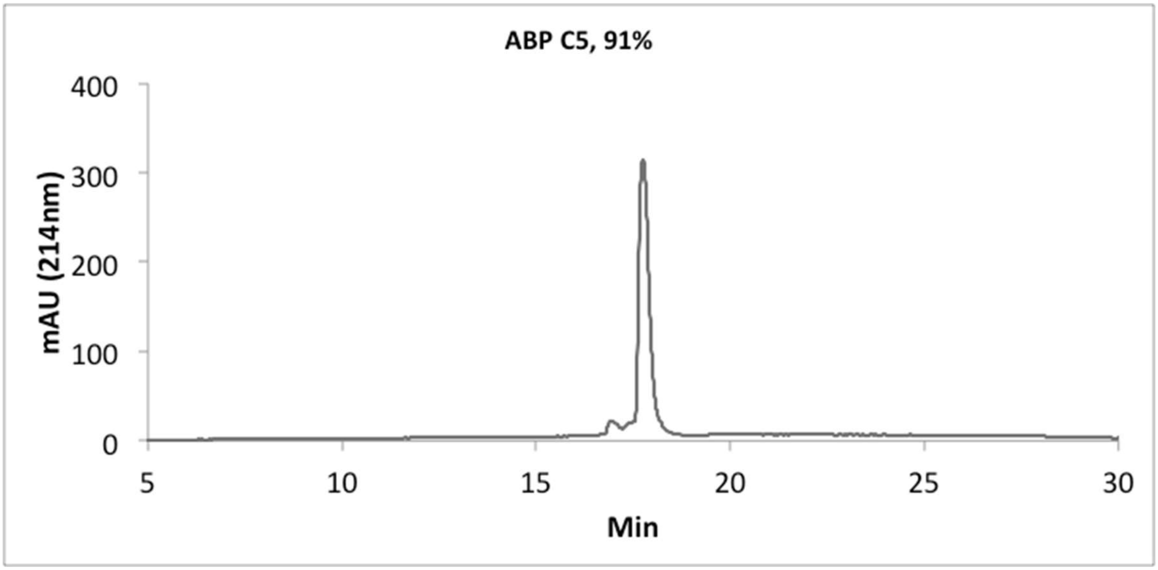
ABP C5, 91%
400
300
200
100
0
mAU (214nm)
5
10
15
20
25
30
Min

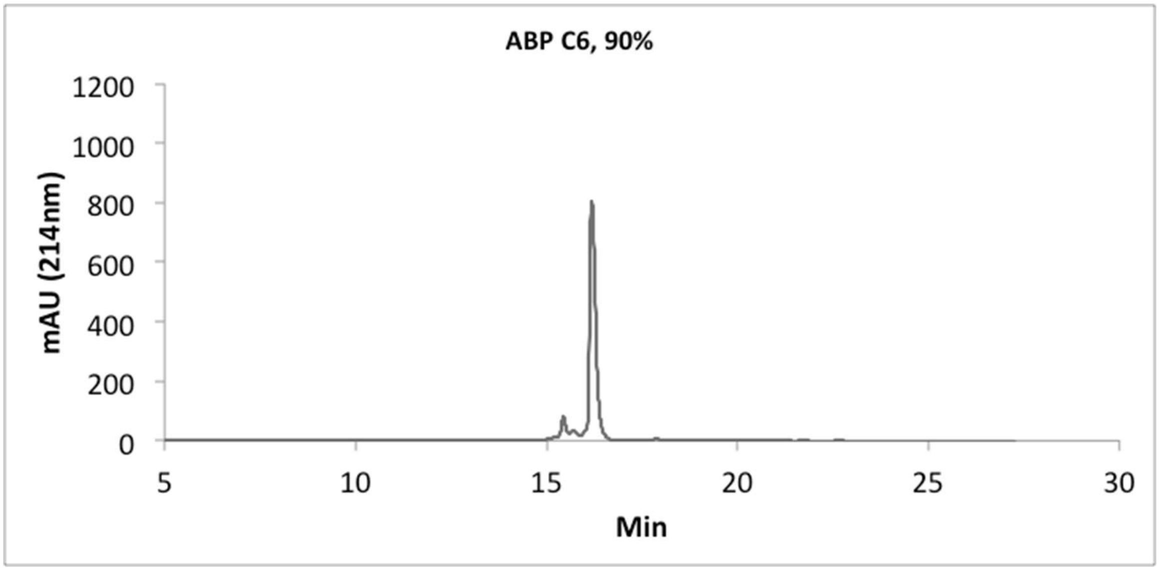
ABP C6, 90%
mAU (214nm)
1200
1000
800
600
400
200
0
5
10
15
20
25
30
Min

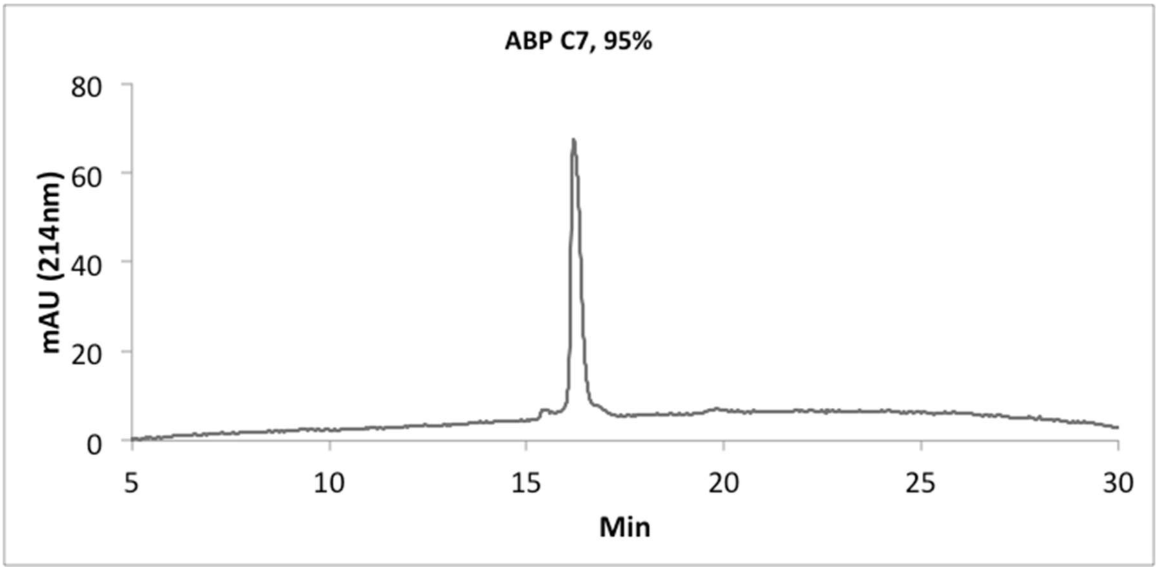
ABP C7, 95%
mAU (214nm)
80
60
40
20
0
5
10
15
20
25
30
Min

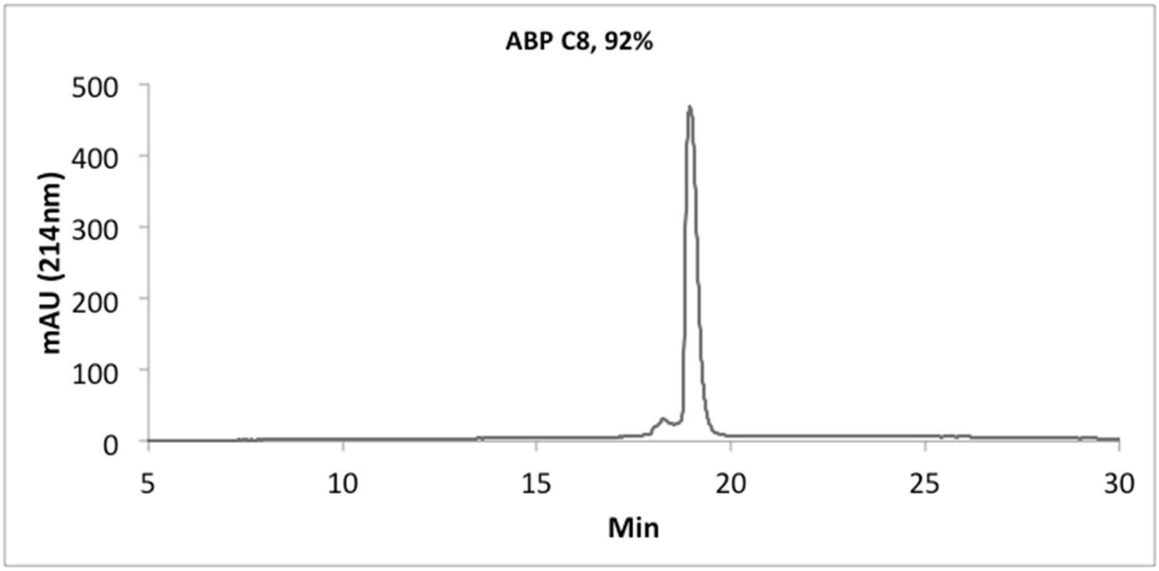
ABP C8, 92%
mAU (214nm)
500
400
300
200
100
0
5
10
15
20
25
30
Min

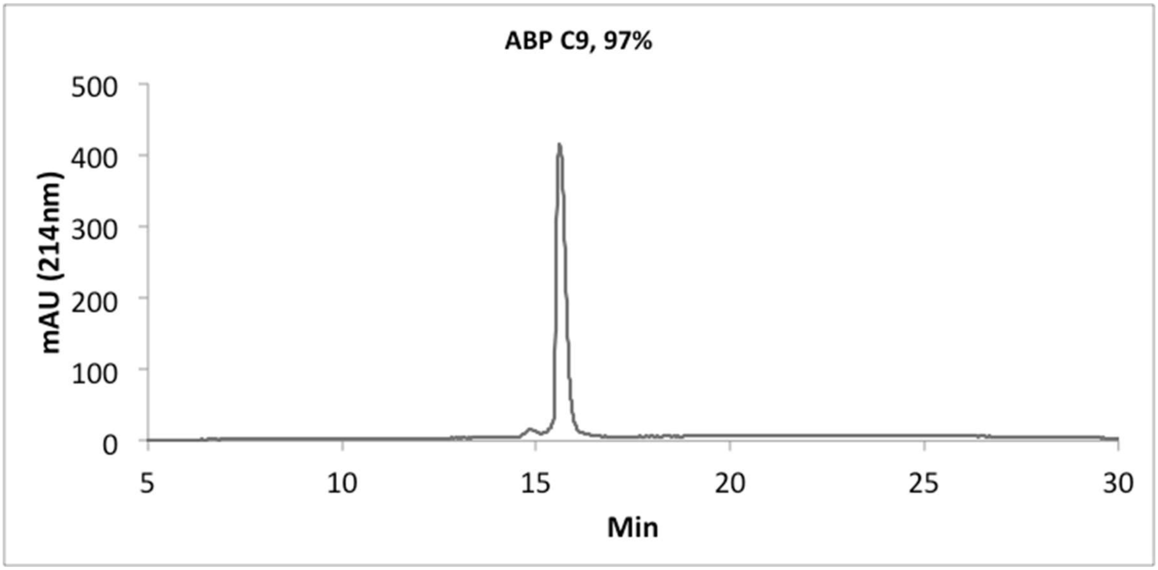
ABP C9, 97%
mAU (214nm)
500
400
300
200
100
0
5
10
15
20
25
30
Min

3.4 HPLC trace of canonical peptides (non-biotinylated)

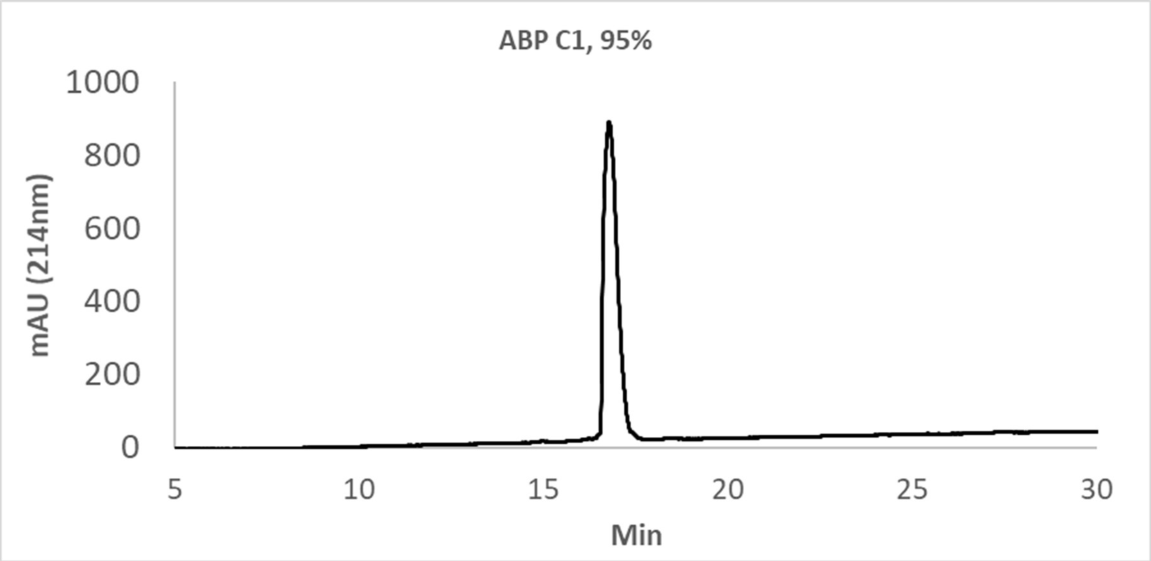

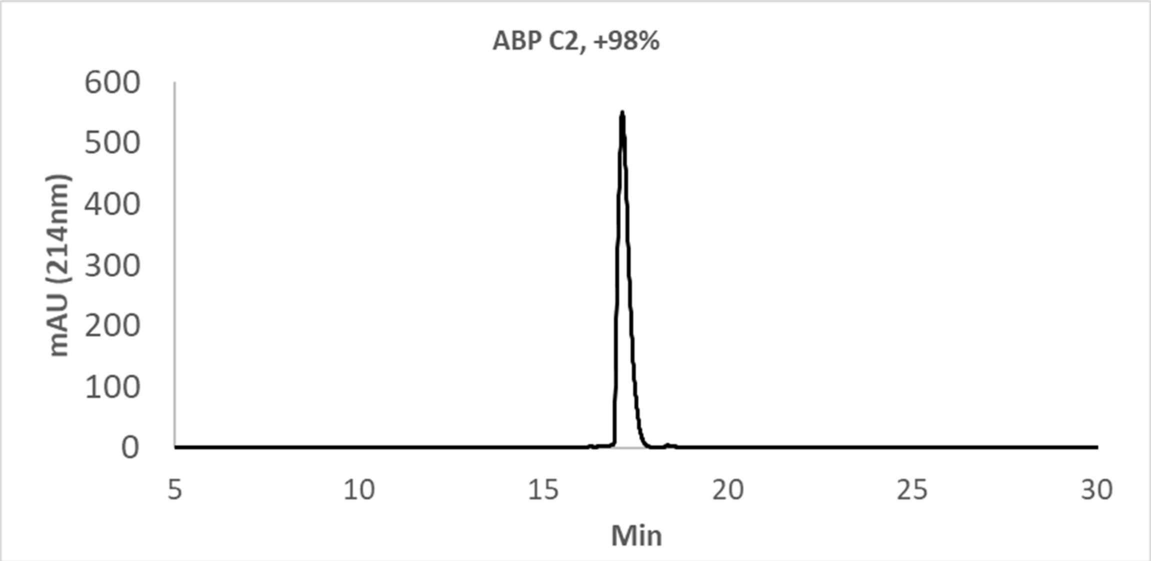

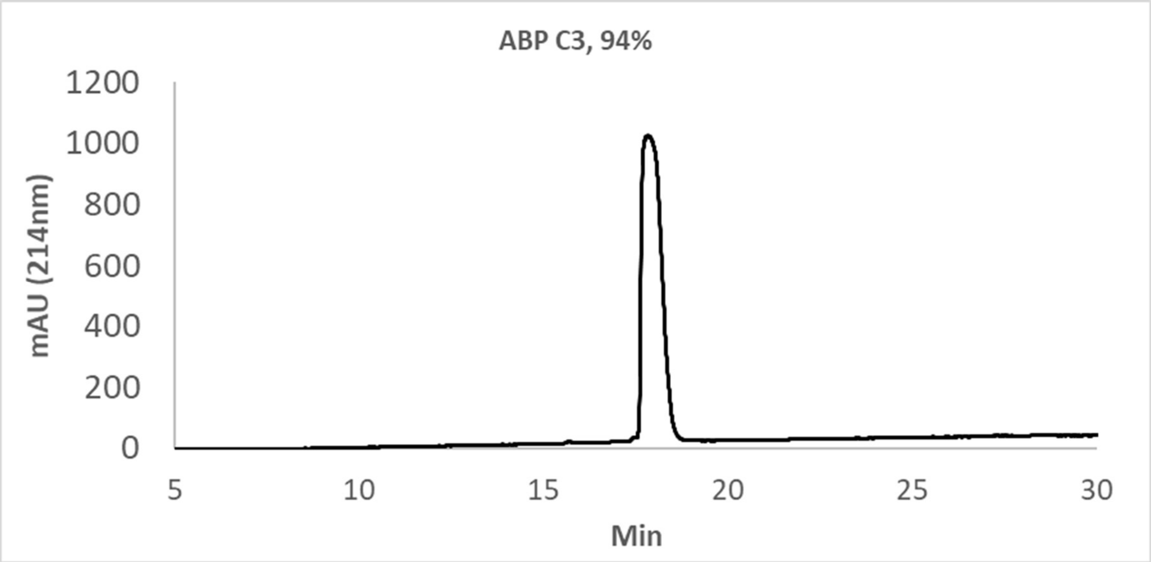
ABP C3, 94%
mAU (214nm)
1200
1000
800
600
400
200
0
5
10
15
20
25
30
Min

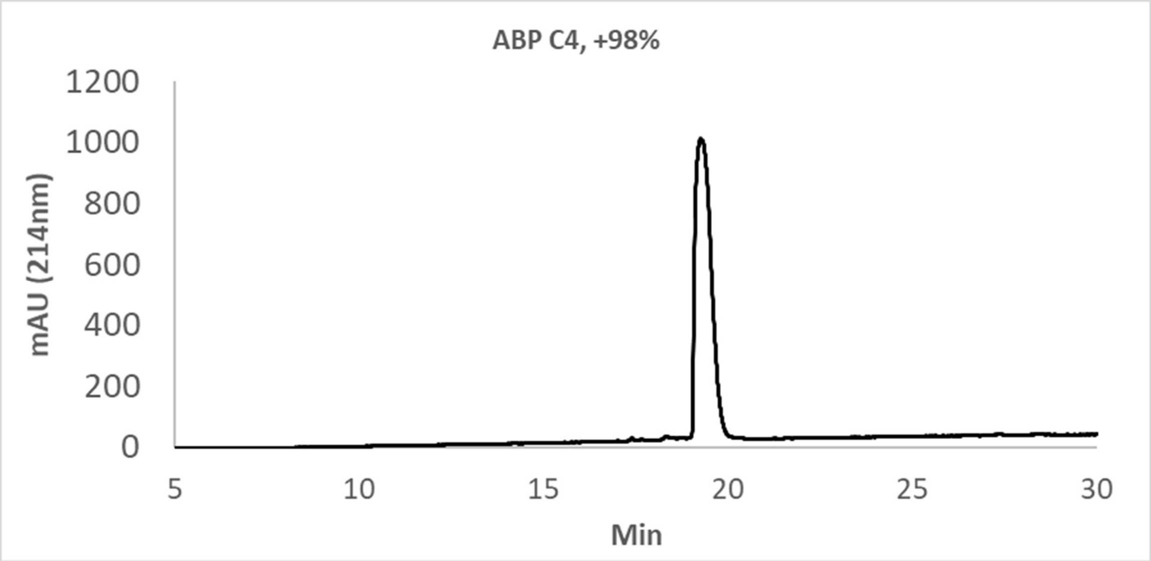
ABP C4, +98%
mAU (214nm)
1200
1000
800
600
400
200
0
5
10
15
20
25
30
Min

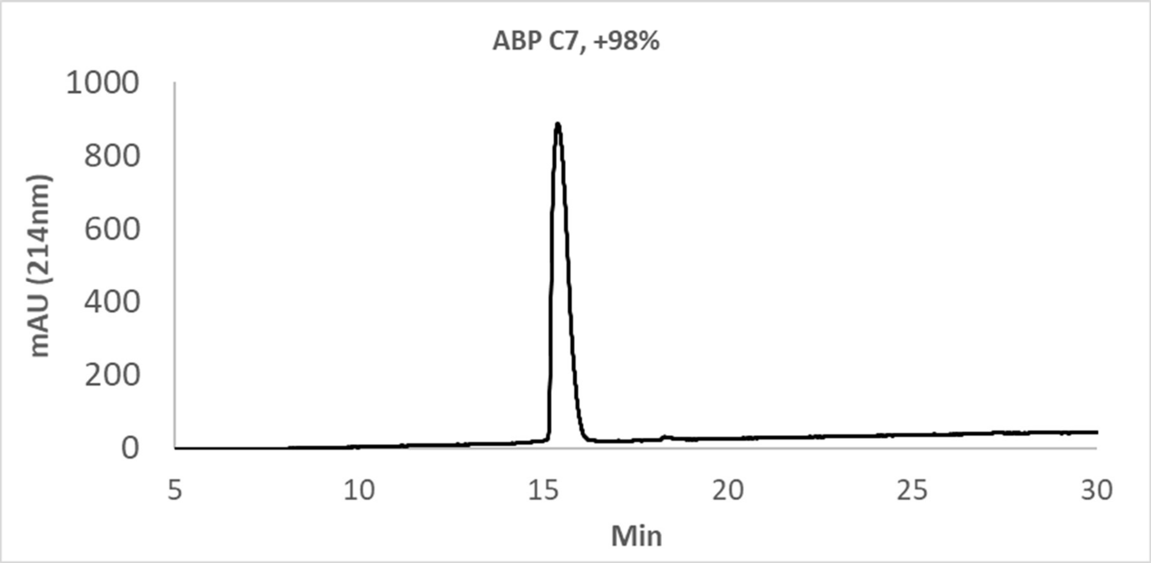
ABP C7, +98%
1000
800
600
400
200
0
mAU (214nm)
5
10
15
20
25
30
Min

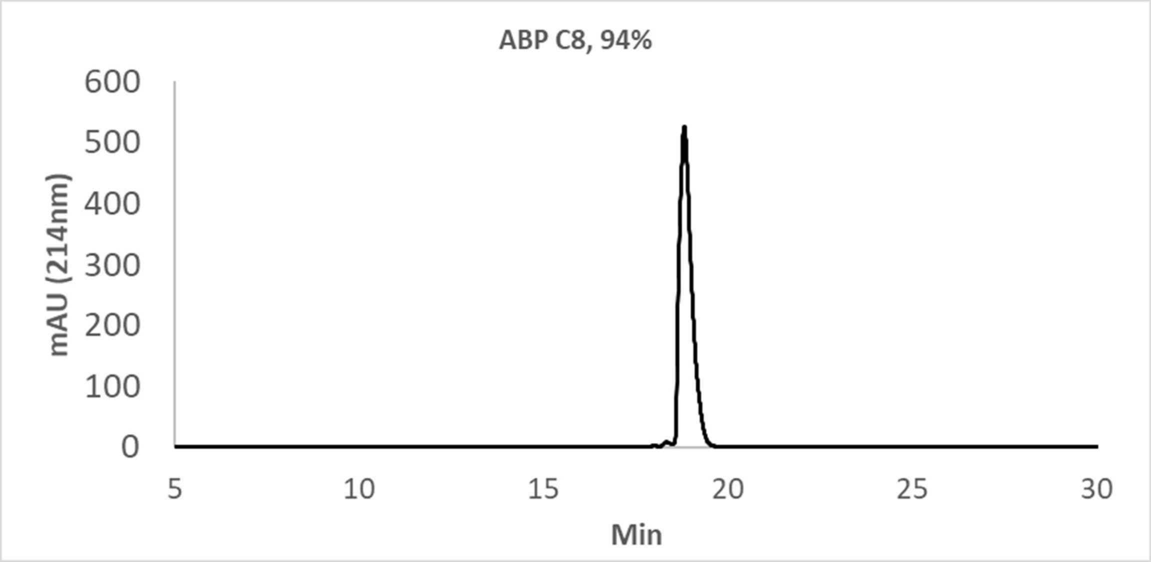
ABP C8, 94%
600
500
400
300
200
100
0
mAU (214nm)
5
10
15
20
25
30
Min

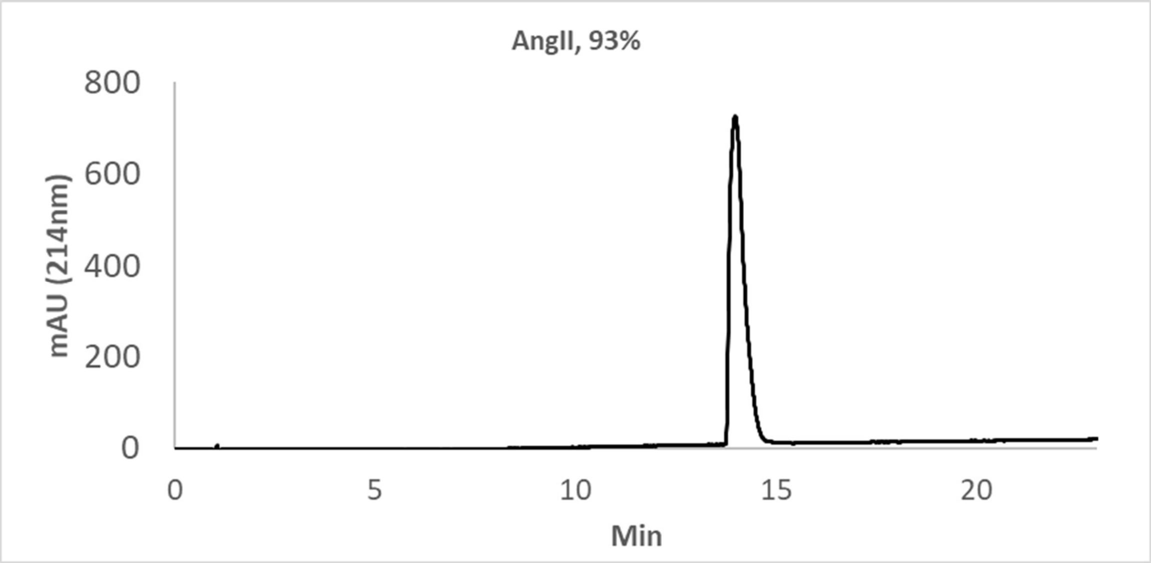
AngII, 93%
800
600
400
200
0
mAU (214nm)
0
5
10
15
20
Min

Supplementary Note 4: Binding affinity measurements

4.1 BLI raw binding data of noncanonical peptides

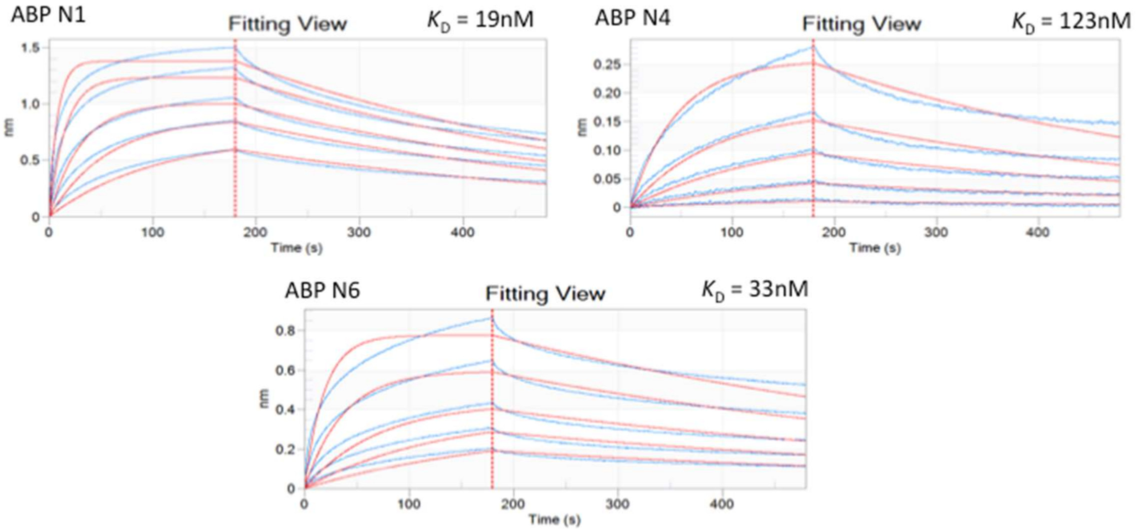

Supplementary Fig. 19. The BLI binding curve fitting (red lines) of ABP N1, ABP N4 and ABP N6 from **Library 2** AS-MS against ACE2. The apparent binding affinity was reported using the kinetic fitting results (K_D is calculated by divide the off rate by on rate). ABP N5 and ABP N8 showed no binding to ACE2. The ACE2 concentration titration (n = 5) is: 1000, 500, 250, 125, and 62.5 nM from top to bottom lines.

4.2 BLI raw binding data of canonical peptides

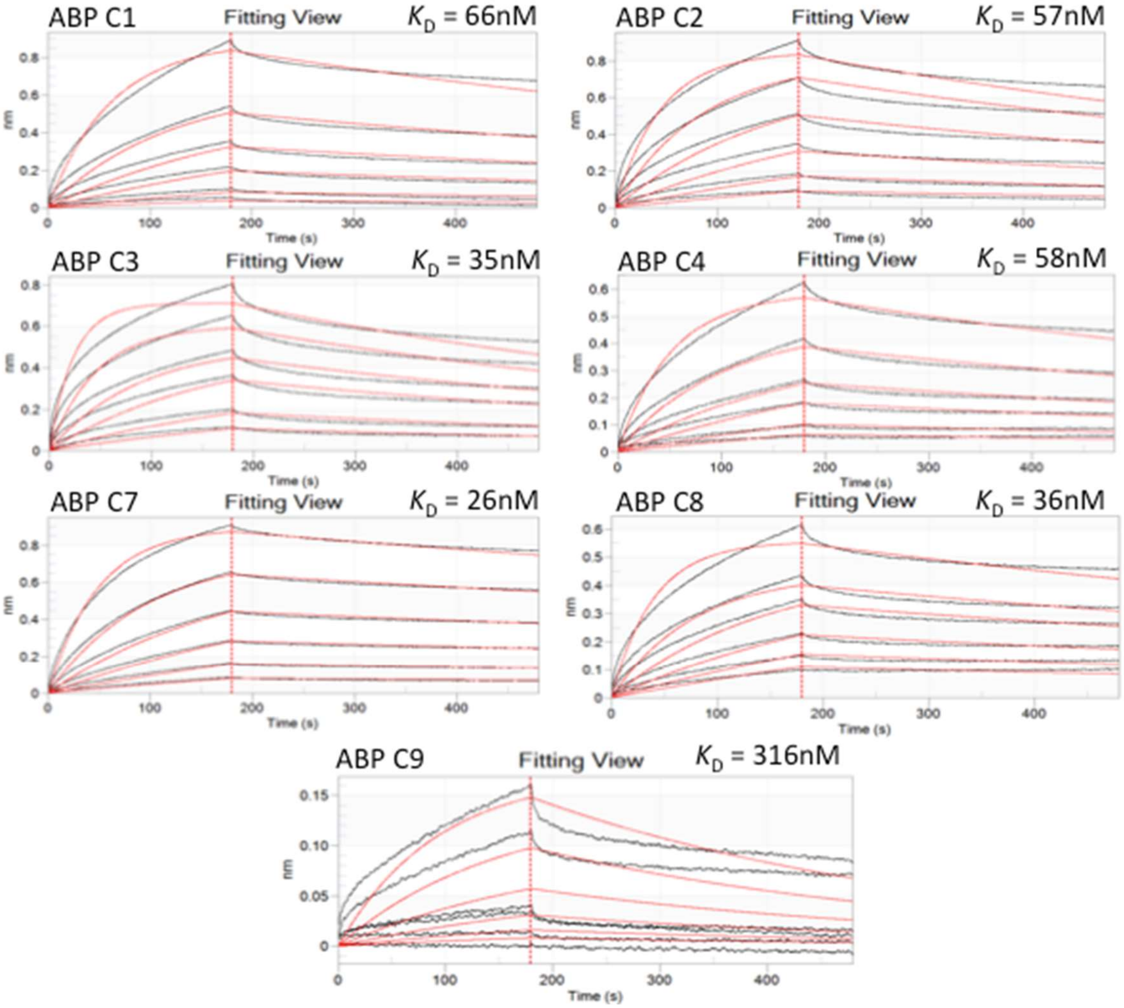

Supplementary Fig. 20. The BLI binding curve fitting (red lines) of ABP C1-C4 and ABP C7-C9 from **Library 1** AS-MS against ACE2. The apparent binding affinity was reported using the kinetic fitting results (K_D is calculated by divide the off rate by on rate). ABP C5 and ABP C6 showed no binding to ACE2. The ACE2 concentration titration (n = 6) is: 1000, 500, 250, 125, 62.5, and 31.3 nM from top to bottom lines.

4.3 The binding of scrambled ABP N1 peptides to ACE2

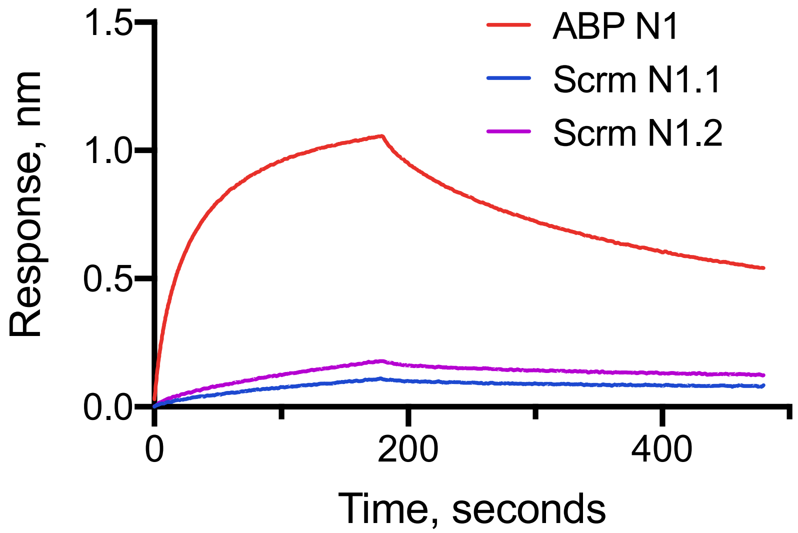

Supplementary Fig. 21. A binding curve comparison of ABP N1 and its scrambled sequences, Scrm N1.1 and Scrm N1.2, measured by BLI. The tested ACE2 concentration was 250 nM. No apparent binding was observed from neither scrambled peptides.

4.4 The binding of ABP noncanonical peptides to an unrelated protein

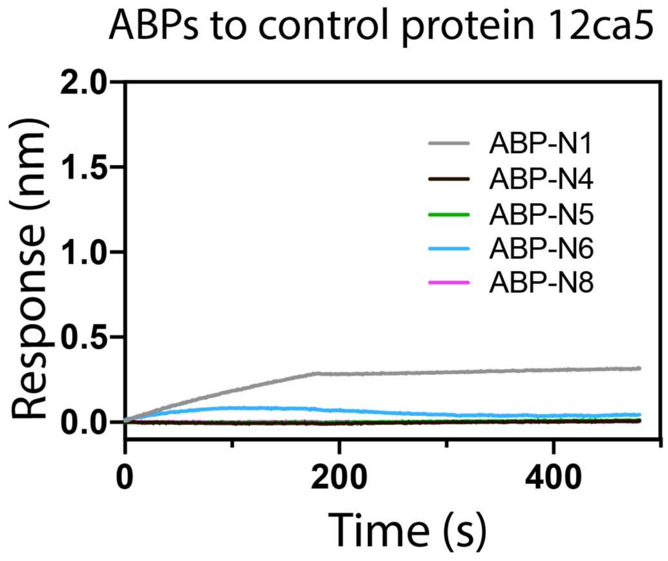

Supplementary Fig. 22. No apparent binding was observed for ABP N1, N4, N5, N6, and N8 to an unrelated protein, 12ca5, measured by BLI at concentration 500 nM.

Supplementary Note 5: Binding competition of SARS-CoV-2 RBD-ACE2 interaction

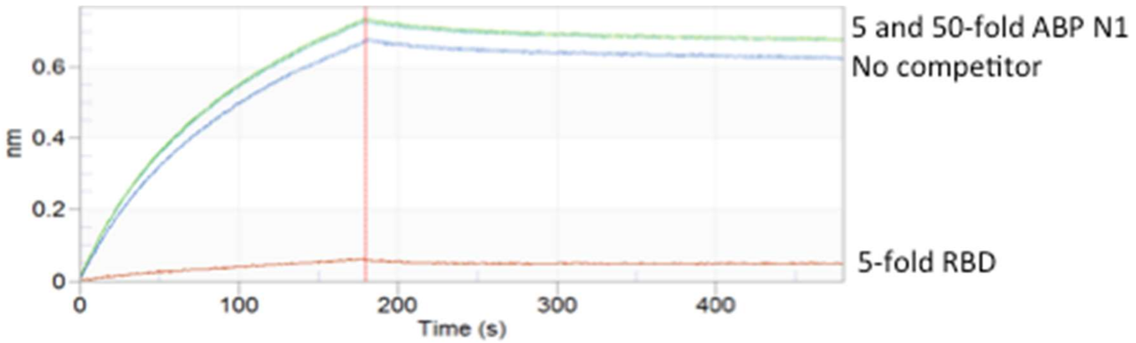

Supplementary Fig. 23. ABP N1 does not interfere the binding of ACE2 and SARS-CoV-2 RBD. A binding competition assay was performed using BLI. RBD protein was immobilized and dipped into either ACE2 or ACE2 mixed with RBD (the positive control), or ACE2 mixed with ABP N1 peptide. As shown from above binding curves, robust inhibition was observed by 5-fold (over ACE2) soluble RBD, but not by ABP N1 peptides at either 5-fold or 50-fold excess, indicating no direct inhibition from ABP N1 on the ACE2 and SARS-CoV-2 RBD interaction.

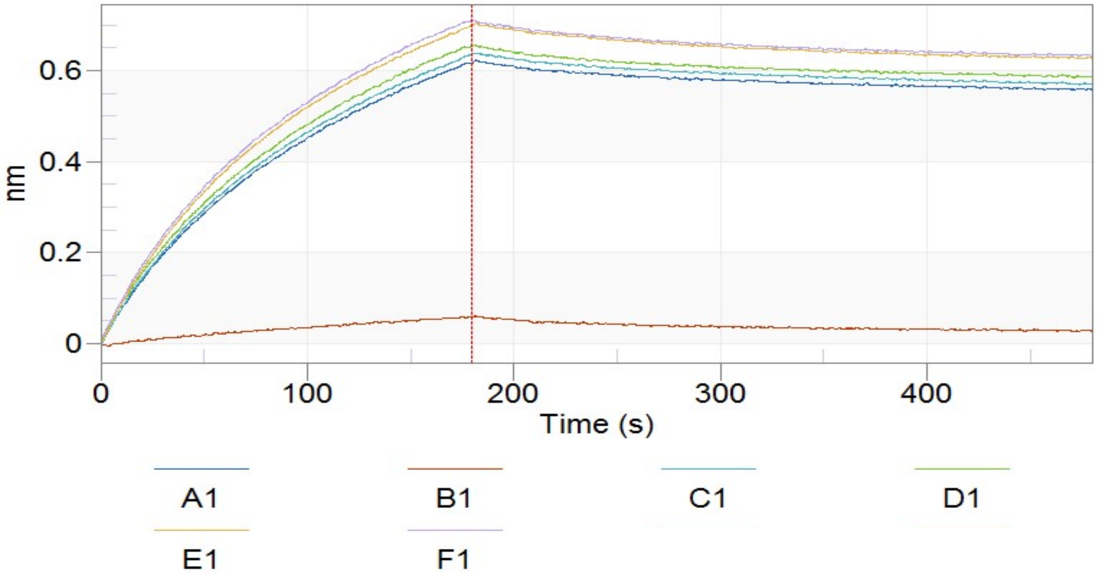

Supplementary Fig. 24. ABP N4 and N6 do not interfere the binding of ACE2 and SARS-CoV-2 RBD. A binding competition assay was performed using BLI.

RBD protein was immobilized and dipped into either ACE2 (100 nM) or ACE2 mixed with RBD (the positive control, at either 0 or 500 nM), or ACE2 mixed with ABP N4 or N6 peptide (500 or 5000 nM). As shown from above binding curves, robust inhibition was observed by 5-fold (over ACE2) soluble RBD, but not by ABP N4 or N6 peptides at either 5-fold or 50-fold excess, indicating no direct inhibition from ABP N4 or N6 on the ACE2 and SARS-CoV-2 RBD interaction. A1: 0 nM RBD; B1: 500 nM RBD; C1: 500 nM ABP N4; D1: 5000 nM ABP N4; E1: 500 nM ABP N6; F1: 5000 nM ABP N6.

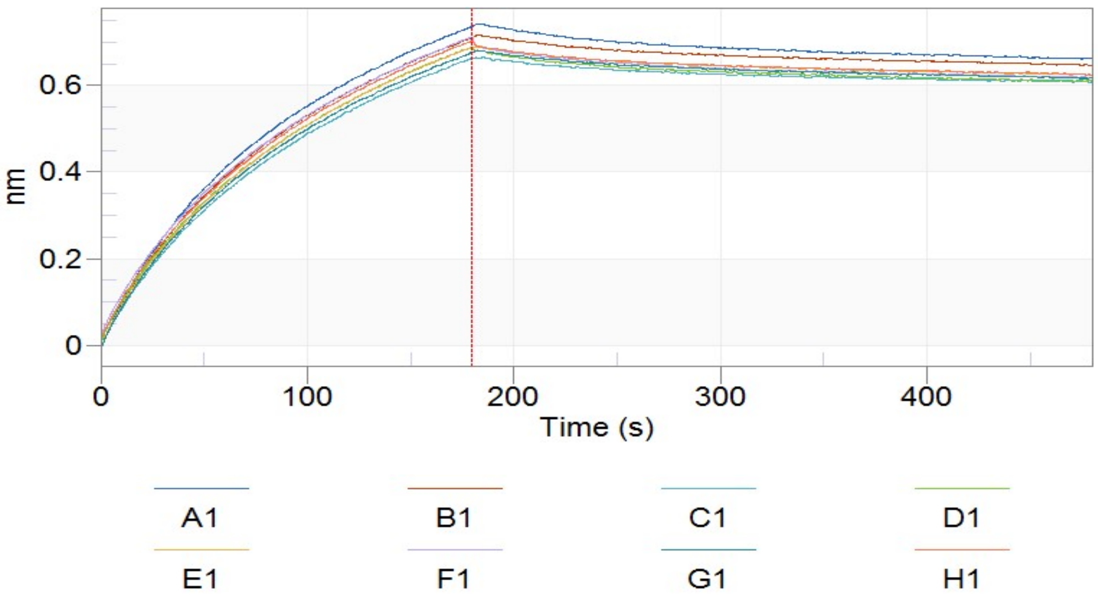

Supplementary Fig. 25. ABP C1, C2, C3, and C4 do not interfere the binding of ACE2 and SARS-CoV-2 RBD. A binding competition assay was performed using BLI. RBD protein was immobilized and dipped into either ACE2 (100 nM) or ACE2 mixed with ABP C1, C2, C3, or C4 peptides (at 500 or 5000 nM). As shown from above binding curves, no obvious inhibition was observed by ABP C1, C2, C3, or C4 peptides at either 5-fold or 50-fold excess, indicating no direct inhibition on the ACE2 and SARS-CoV-2 RBD interaction. A1: 500 nM ABP C1; B1: 5000 nM ABP C1; C1: 500 nM ABP C2; D1: 5000 nM ABP C2; E1: 500 nM ABP C3; F1: 5000 nM ABP C3; G1: 500 nM ABP C4; H1: 5000 nM ABP C4.

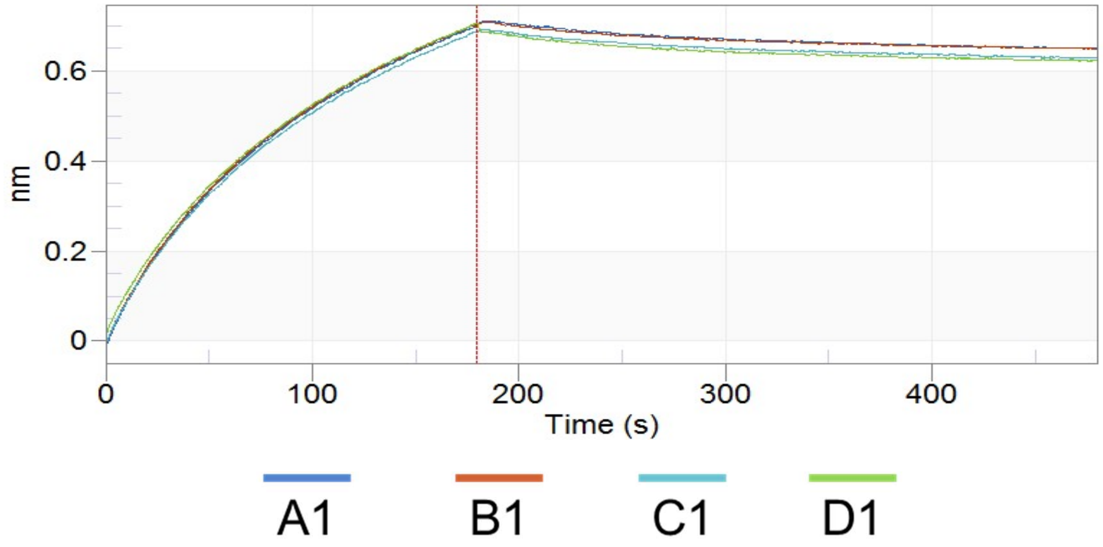

Supplementary Fig. 26. ABP C7 and C8 do not interfere the binding of ACE2 and SARS-CoV-2 RBD. A binding competition assay was performed using BLI. RBD protein was immobilized and dipped into either ACE2 (100 nM) or ACE2 mixed with ABP C7 or C8 peptides (at 500 or 5000 nM). As shown from above binding curves, no obvious inhibition was observed by ABP C7 or C8 peptides at either 5-fold or 50-fold excess, indicating no direct inhibition on the ACE2 and SARS-CoV-2 RBD interaction. A1: 500 nM ABP C7; B1: 5000 nM ABP C7; C1: 500 nM ABP C8; D1: 5000 nM ABP C8.

Supplementary Note 6: Binding competition of known ACE2 inhibitors to ABPs-ACE2 interaction

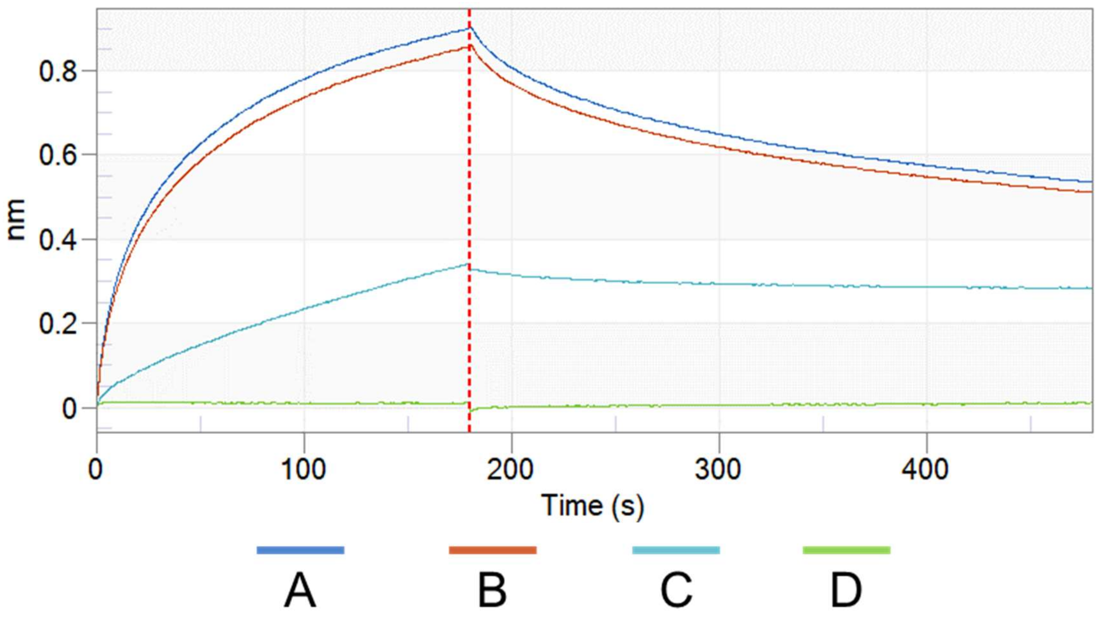

Supplementary Fig. 27. The binding of ABP N1 is partially inhibited by ACE2 inhibitor MLN-4760 and not inhibited by AngII. A binding competition assay was performed using BLI. Biotinylated peptide was immobilized dipped into either ACE2 (500 nM, "A" above in blue) or ACE2 mixed with AngII in 10-fold excess (5 uM, "B" in red) or ACE2 mixed with MLN-4760 in 10-fold excess (5 uM, "C" in cyan). A no protein control (0 nM ACE) is shown in "D" in green to observed any nonspecific BSA binding to the immobilized peptide. As shown from above binding curves, no obvious inhibition was observed by the AngII peptide at 10-fold excess. However, MLN-4760 decreased the binding of ACE2 by more than half, indicating that ABP N1 binding is inhibited. Immobilized: ABP N1; A: 500 nM ACE2; B: 500 nM ACE2 + 5 uM AngII; C: 500 nM ACE2 + 5 uM MLN-4760; D: 0 nM ACE2.

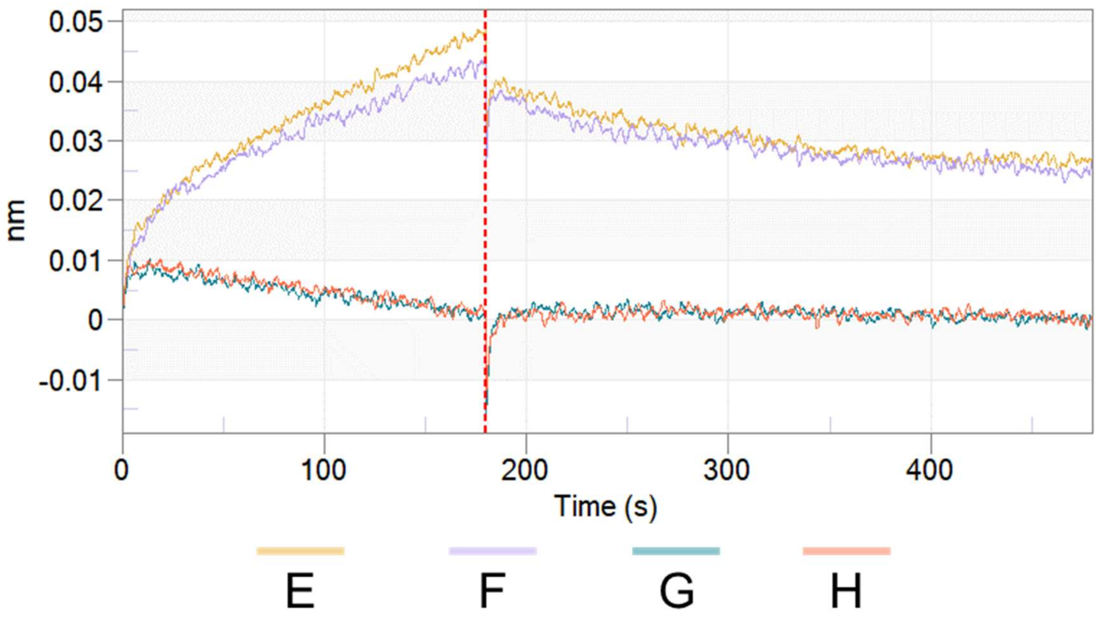

Supplementary Fig. 28. The binding of ABP N4 is completely inhibited by ACE2 inhibitor MLN-4760 and not inhibited by AngII. A binding competition assay was performed using BLI. Biotinylated peptide was immobilized dipped into either ACE2 (500 nM, "E" above in gold) or ACE2 mixed with AngII in 10-fold excess (5 uM, "F" in lavender) or ACE2 mixed with MLN-4760 in 10-fold excess (5 uM, "G" in teal). A no protein control (0 nM ACE) is shown in "H" in orange to observed any nonspecific BSA binding to the immobilized peptide. As shown from above binding curves, no obvious inhibition was observed by the AngII peptide at 10-fold excess. However, MLN-4760 decreased the binding of ACE2 completely, indicating that ABP N4 binding is inhibited. Immobilized: ABP N4; E: 500 nM ACE2; F: 500 nM ACE2 + 5 uM AngII; G: 500 nM ACE2 + 5 uM MLN-4760; H: 0 nM ACE2.

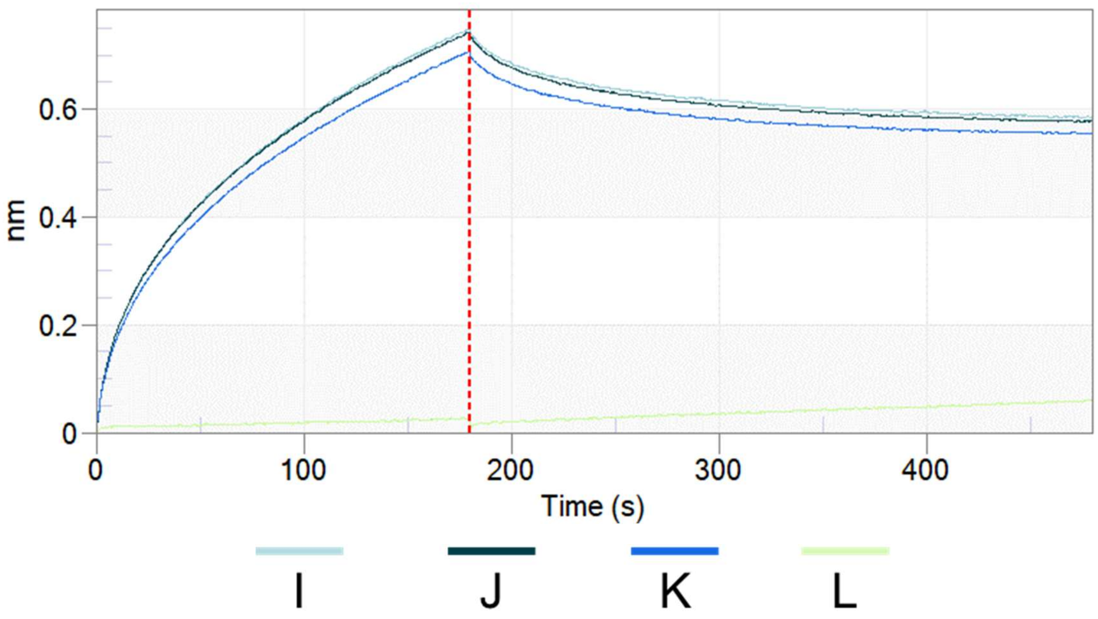

Supplementary Fig. 29. The binding of ABP N6 is not inhibited by ACE2 inhibitor MLN-4760 or AngII. A binding competition assay was performed using BLI. Biotinylated peptide was immobilized dipped into either ACE2 (500 nM, “I” above in aqua) or ACE2 mixed with AngII in 10-fold excess (5 uM, “J” in dark green) or ACE2 mixed with MLN-4760 in 10-fold excess (5 uM, “K” in royal blue). A no protein control (0 nM ACE) is shown in “L” in light green to observed any nonspecific BSA binding to the immobilized peptide. As shown from above binding curves, no obvious inhibition was observed by the AngII peptide or MLN-4760 at 10-fold excess. Immobilized: ABP N6; I: 500 nM ACE2; J: 500 nM ACE2 + 5 uM AngII; K: 500 nM ACE2 + 5 uM MLN-4760; L: 0 nM ACE2.

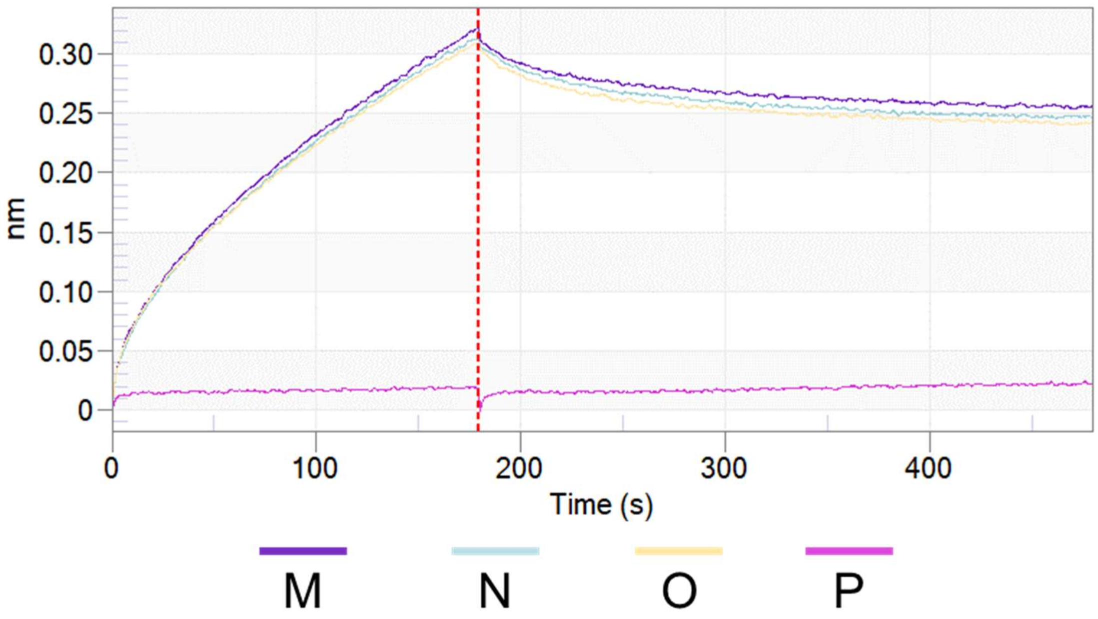

Supplementary Fig. 30. The binding of ABP C1 is not inhibited by ACE2 inhibitor MLN-4760 or AngII. A binding competition assay was performed using BLI. Biotinylated peptide was immobilized dipped into either ACE2 (500 nM, "M" above in purple) or ACE2 mixed with AngII in 10-fold excess (5 uM, "N" in aqua) or ACE2 mixed with MLN-4760 in 10-fold excess (5 uM, "O" in light yellow). A no protein control (0 nM ACE) is shown in "P" in magenta to observed any nonspecific BSA binding to the immobilized peptide. As shown from above binding curves, no obvious inhibition was observed by the AngII peptide or MLN-4760 at 10-fold excess. Immobilized: ABP C1; M: 500 nM ACE2; N: 500 nM ACE2 + 5 uM AngII; O: 500 nM ACE2 + 5 uM MLN-4760; P: 0 nM ACE2.

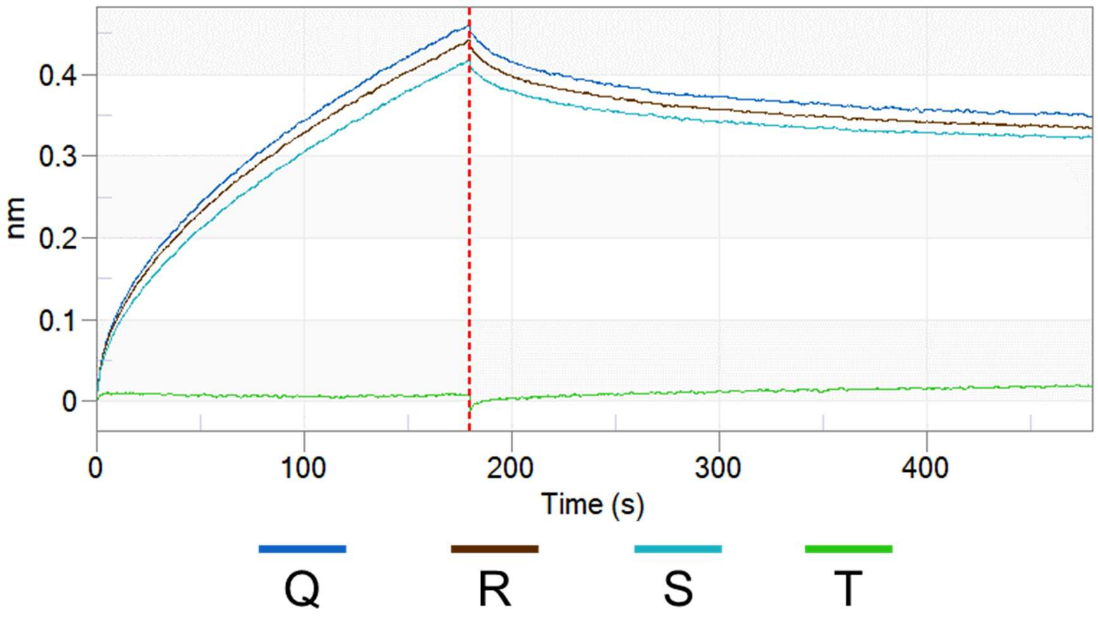

Supplementary Fig. 31. The binding of ABP C2 is not inhibited by ACE2 inhibitor MLN-4760 or AngII. A binding competition assay was performed using BLI. Biotinylated peptide was immobilized dipped into either ACE2 (500 nM, “Q” above in blue) or ACE2 mixed with AngII in 10-fold excess (5 uM, “R” in brown) or ACE2 mixed with MLN-4760 in 10-fold excess (5 uM, “S” in aqua). A no protein control (0 nM ACE) is shown in “T” in green to observed any nonspecific BSA binding to the immobilized peptide. As shown from above binding curves, no obvious inhibition was observed by the AngII peptide or MLN-4760 at 10-fold excess. Immobilized: ABP C2; Q: 500 nM ACE2; R: 500 nM ACE2 + 5 uM AngII; S: 500 nM ACE2 + 5 uM MLN-4760; T: 0 nM ACE2.

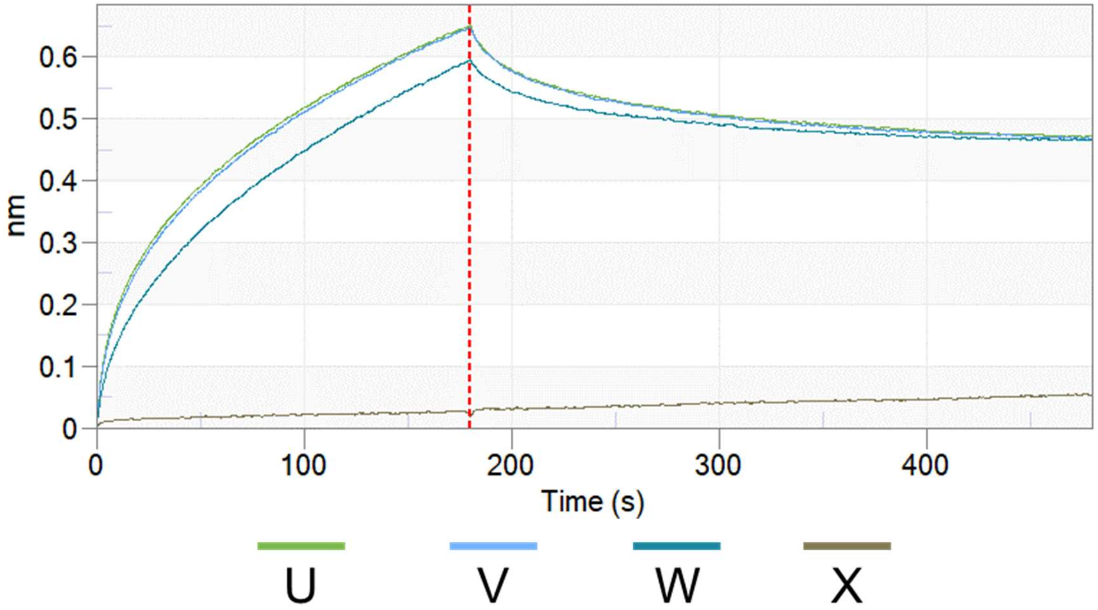

Supplementary Fig. 32. The binding of ABP C3 is not inhibited by ACE2 inhibitor MLN-4760 or AngII. A binding competition assay was performed using BLI. Biotinylated peptide was immobilized dipped into either ACE2 (500 nM, “U” above in green) or ACE2 mixed with AngII in 10-fold excess (5 uM, “V” in light blue) or ACE2 mixed with MLN-4760 in 10-fold excess (5 uM, “W” in teal). A no protein control (0 nM ACE) is shown in “X” in brown to observed any nonspecific BSA binding to the immobilized peptide. As shown from above binding curves, no obvious inhibition was observed by the AngII peptide or MLN-4760 at 10-fold excess. Immobilized: ABP C3; U: 500 nM ACE2; V: 500 nM ACE2 + 5 uM AngII; W: 500 nM ACE2 + 5 uM MLN-4760; X: 0 nM ACE2.

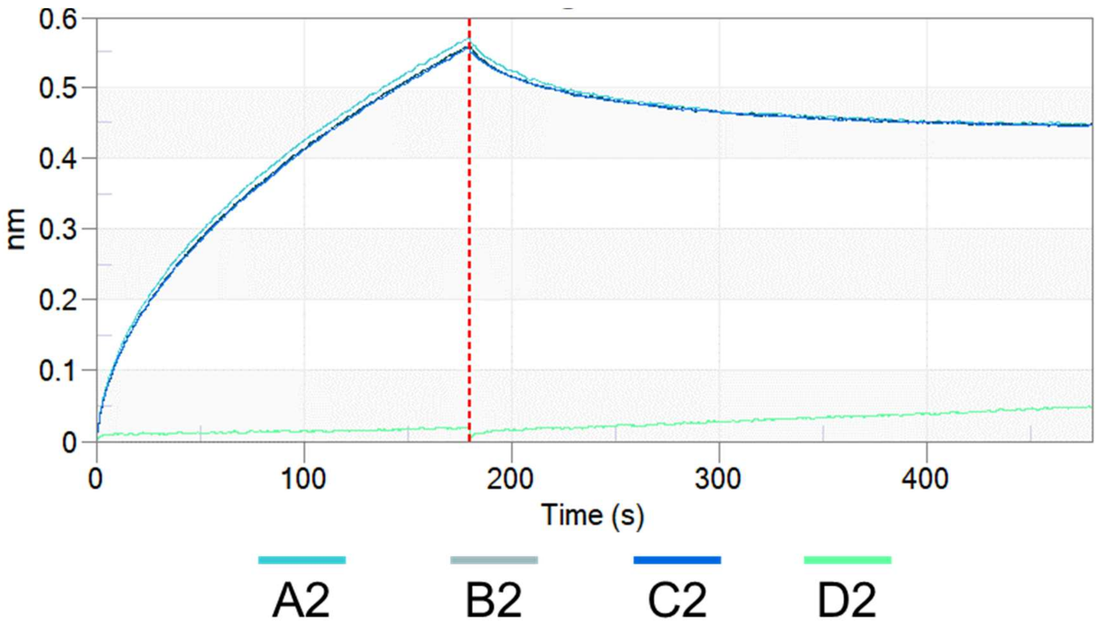

Supplementary Fig. 33. The binding of ABP C4 is not inhibited by ACE2 inhibitor MLN-4760 or AngII. A binding competition assay was performed using BLI. Biotinylated peptide was immobilized dipped into either ACE2 (500 nM, “A2” above in cyan) or ACE2 mixed with AngII in 10-fold excess (5 uM, “B2” in gray) or ACE2 mixed with MLN-4760 in 10-fold excess (5 uM, “C2” in blue). A no protein control (0 nM ACE) is shown in “D2” in lime green to observed any nonspecific BSA binding to the immobilized peptide. As shown from above binding curves, no obvious inhibition was observed by the AngII peptide or MLN-4760 at 10-fold excess. Immobilized: ABP C4; A2: 500 nM ACE2; B2: 500 nM ACE2 + 5 uM AngII; C2: 500 nM ACE2 + 5 uM MLN-4760; D2: 0 nM ACE2.

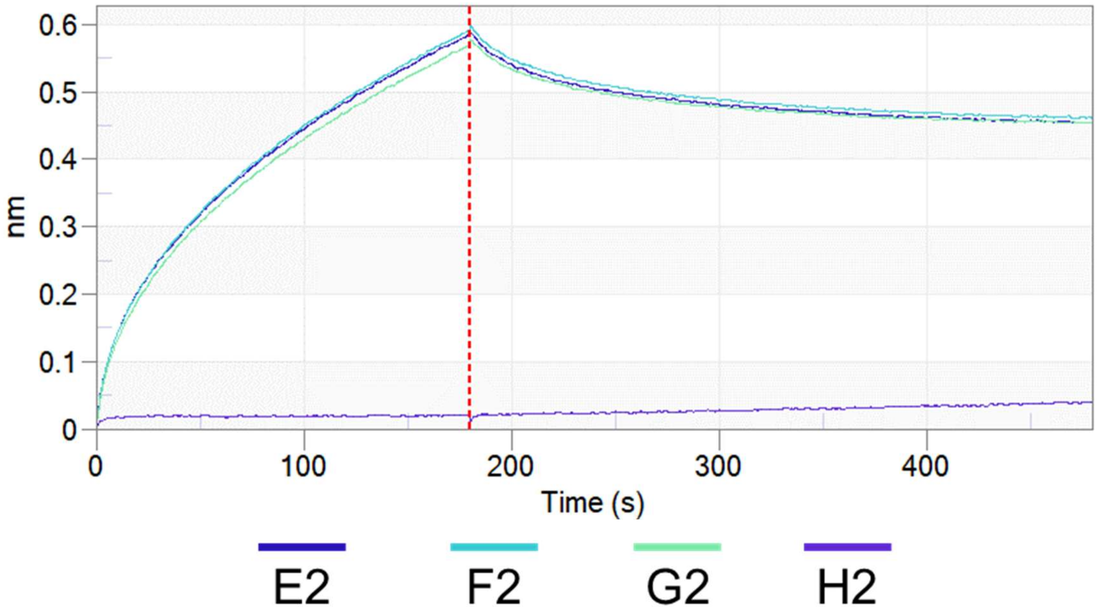

Supplementary Fig. 34. The binding of ABP C7 is not inhibited by ACE2 inhibitor MLN-4760 or AngII. A binding competition assay was performed using BLI. Biotinylated peptide was immobilized dipped into either ACE2 (500 nM, "E2" above in dark purple) or ACE2 mixed with AngII in 10-fold excess (5 uM, "F2" in cyan) or ACE2 mixed with MLN-4760 in 10-fold excess (5 uM, "G2" in lime green). A no protein control (0 nM ACE) is shown in "H2" in purple to observed any nonspecific BSA binding to the immobilized peptide. As shown from above binding curves, no obvious inhibition was observed by the AngII peptide or MLN-4760 at 10-fold excess. Immobilized: ABP C7; E2: 500 nM ACE2; F2: 500 nM ACE2 + 5 uM AngII; G2: 500 nM ACE2 + 5 uM MLN-4760; H2: 0 nM ACE2.

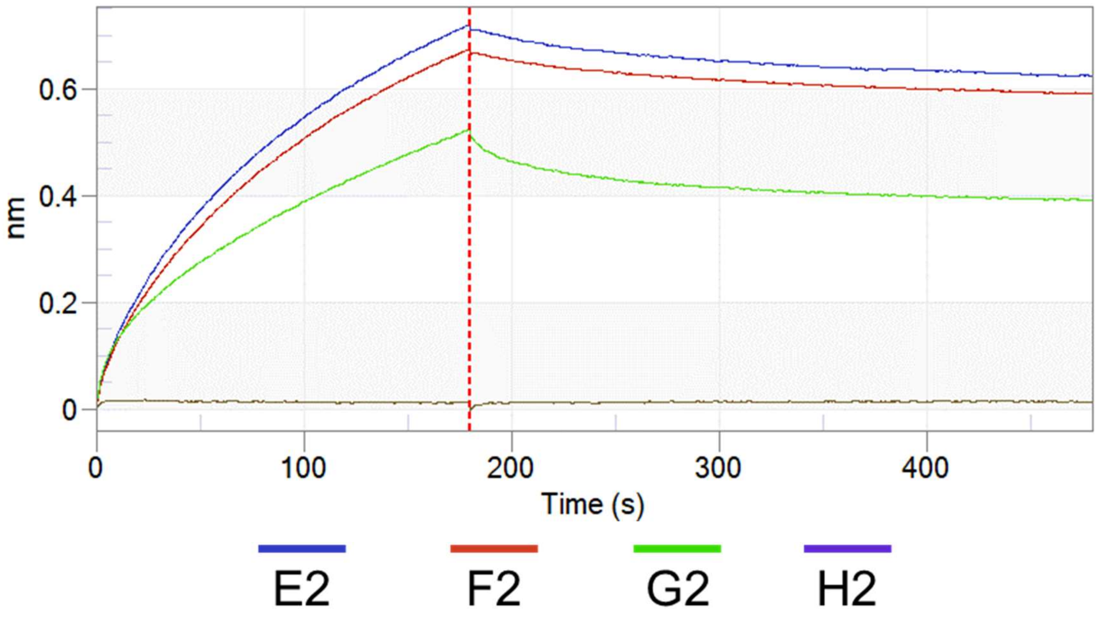

Supplementary Fig. 35. The binding of ABP C8 is partially inhibited by ACE2 inhibitor MLN-4760 and not inhibited by AngII. A binding competition assay was performed using BLI. Biotinylated peptide was immobilized dipped into either ACE2 (500 nM, “E2” above in dark blue) or ACE2 mixed with AngII in 10-fold excess (5 uM, “F2” in red) or ACE2 mixed with MLN-4760 in 10-fold excess (5 uM, “G2” in green). A no protein control (0 nM ACE) is shown in “H2” in purple to observed any nonspecific BSA binding to the immobilized peptide. As shown from above binding curves, no obvious inhibition was observed by the AngII peptide at 10-fold excess. However, MLN-4760 decreased the binding of ACE2 by less than half, indicating that ABP C8 binding is inhibited. Immobilized: ABP C8; E2: 500 nM ACE2; F2: 500 nM ACE2 + 5 uM AngII; G2: 500 nM ACE2 + 5 uM MLN-4760; H2: 0 nM ACE2.
