# Supplementary material for: Rapid de novo discovery of peptidomimetic affinity reagents for human angiotensin converting enzyme 2
Source: Commun Chem. 2022 Jan 19;5:8. doi: 10.1038/s42004-022-00625-3 (PMC9814530; doi:10.1038/s42004-022-00625-3)
Supplement: Supplementary file 2 — Reporting Summary [file 42004_2022_625_MOESM2_ESM.pdf]

nature research

Corresponding author(s): Bradley L Pentelute

Last updated by author(s): 12/15/2021

# Reporting Summary

Nature Research wishes to improve the reproducibility of the work that we publish. This form provides structure for consistency and transparency in reporting. For further information on Nature Research policies, see our Editorial Policies and the Editorial Policy Checklist.

## Statistics

For all statistical analyses, confirm that the following items are present in the figure legend, table legend, main text, or Methods section.

| n/a | Confirmed | |
|---|---|---|
| ☐ | ☒ | The exact sample size (*n*) for each experimental group/condition, given as a discrete number and unit of measurement |
| ☐ | ☒ | A statement on whether measurements were taken from distinct samples or whether the same sample was measured repeatedly |
| ☐ | ☒ | The statistical test(s) used AND whether they are one- or two-sided<br>*Only common tests should be described solely by name; describe more complex techniques in the Methods section.* |
| ☒ | ☐ | A description of all covariates tested |
| ☒ | ☐ | A description of any assumptions or corrections, such as tests of normality and adjustment for multiple comparisons |
| ☐ | ☒ | A full description of the statistical parameters including central tendency (e.g. means) or other basic estimates (e.g. regression coefficient) AND variation (e.g. standard deviation) or associated estimates of uncertainty (e.g. confidence intervals) |
| ☐ | ☒ | For null hypothesis testing, the test statistic (e.g. *F*, *t*, *r*) with confidence intervals, effect sizes, degrees of freedom and *P* value noted<br>*Give P values as exact values whenever suitable.* |
| ☒ | ☐ | For Bayesian analysis, information on the choice of priors and Markov chain Monte Carlo settings |
| ☒ | ☐ | For hierarchical and complex designs, identification of the appropriate level for tests and full reporting of outcomes |
| ☒ | ☐ | Estimates of effect sizes (e.g. Cohen's *d*, Pearson's *r*), indicating how they were calculated |

*Our web collection on statistics for biologists contains articles on many of the points above.*

## Software and code

Policy information about availability of computer code

| | |
|---|---|
| Data collection | Agilent MassHunter, Thermo Fisher Orbitrap Fusion Lumos Tribrid Mass Spectrometer Instrument Control Software, Sartorius Octet Systems Software. |
| Data analysis | Agilent MassHunter B.06.00, PEAKS 8.5, Octet Software for Data Acquisition and Data Analysis 8.2.0.7 |

For manuscripts utilizing custom algorithms or software that are central to the research but not yet described in published literature, software must be made available to editors and reviewers. We strongly encourage code deposition in a community repository (e.g. GitHub). See the Nature Research guidelines for submitting code & software for further information.

## Data

Policy information about availability of data

All manuscripts must include a data availability statement. This statement should provide the following information, where applicable:

- Accession codes, unique identifiers, or web links for publicly available datasets
- A list of figures that have associated raw data
- A description of any restrictions on data availability

All the data generated or analyzed during this study are included in this published article (and in the Supplementary Material). Further details are available from the corresponding authors upon request.

# Field-specific reporting

Please select the one below that is the best fit for your research. If you are not sure, read the appropriate sections before making your selection.

☒ Life sciences ☐ Behavioural & social sciences ☐ Ecological, evolutionary & environmental sciences

For a reference copy of the document with all sections, see nature.com/documents/nr-reporting-summary-flat.pdf

# Life sciences study design

All studies must disclose on these points even when the disclosure is negative.

| | |
|---|---|
| Sample size | All experiments of the automated and/or manual synthesis of peptides were carried out with n=1 sample. The binding affinity measurement experiments were carried out with n=1 sample at each indicated concentration point. The serum stability experiments were carried out with n=1 samples. These experiments use robust analytical conditions and simple experimental setups so simplified experimental design with one data point is sufficient to observe data trends. For ELISA assays, samples were run in triplicates for each sample concentration point. |
| Data exclusions | All data are included for each experiment described. |
| Replication | All ELISA assays were performed in three technical replicates for each peptide sample at tested concentrations. |
| Randomization | Both synthetic peptide libraries were prepared with full randomization by solid-phase peptide synthesis (SPPS) (split-pool synthesis). |
| Blinding | Blinding was not relevant to our study, because biased interpretation is not likely due to the quantitative nature of the measurements used in the study. |

# Reporting for specific materials, systems and methods

We require information from authors about some types of materials, experimental systems and methods used in many studies. Here, indicate whether each material, system or method listed is relevant to your study. If you are not sure if a list item applies to your research, read the appropriate section before selecting a response.

## Materials & experimental systems

| n/a | Involved in the study | |
|---|---|---|
| ☒ | ☐ | Antibodies |
| ☒ | ☐ | Eukaryotic cell lines |
| ☒ | ☐ | Palaeontology and archaeology |
| ☒ | ☐ | Animals and other organisms |
| ☒ | ☐ | Human research participants |
| ☒ | ☐ | Clinical data |
| ☒ | ☐ | Dual use research of concern |

## Methods

| n/a | Involved in the study | |
|---|---|---|
| ☒ | ☐ | ChIP-seq |
| ☒ | ☐ | Flow cytometry |
| ☒ | ☐ | MRI-based neuroimaging |
